# Regioconvergent Nucleophilic Substitutions with Morita–Baylis–Hillman Fluorides

**DOI:** 10.1021/acs.joc.4c00660

**Published:** 2024-07-17

**Authors:** Jeffrey S. S. K. Formen, Ciarán C. Lynch, Eryn Nelson, Andi Yuan, Sarah E. Steber, Christian Wolf

**Affiliations:** Department of Chemistry, Georgetown University, 37th and O Streets, Washington, District of Columbia 20057, United States

## Abstract

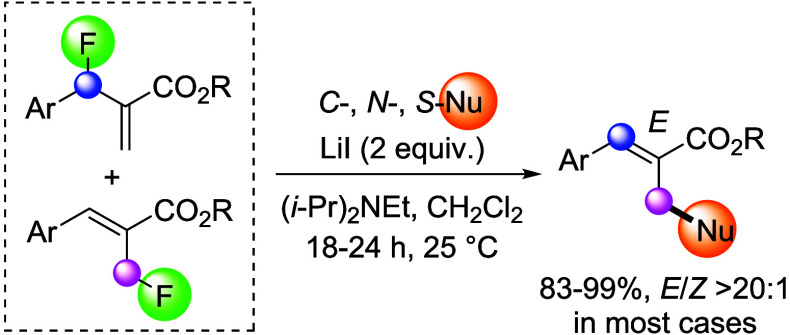

Lithium iodide enables regioconvergent C–F bond functionalization of isomeric Morita–Baylis–Hillman fluorides with carbon, sulfur, and nitrogen nucleophiles. The defluorinative carbon–carbon and carbon–heteroatom bond formations give multifunctional compounds in excellent yields and with good to high diastereoselectivities at room temperature. The possibility of catalytic enantioselective allylation is also discussed.

## Introduction

The general significance of fluorinated organic compounds in the life sciences has stimulated the introduction of many practical methods to make them.^1,2^ Organofluorines have become readily available starting materials that are rapidly growing in popularity among synthetic chemists. The usefulness of aryl fluorides in nucleophilic aromatic substitution and transition metal-catalyzed cross-coupling reactions is well documented. C_sp2_–F functionalization is a frequently employed venue to form carbon–carbon or carbon–heteroatom bonds, while applications of aliphatic substrates are less explored. Activation of a C_sp3_–F bond often requires strong Lewis acids and harsh conditions that may favor competing hydrodefluorination pathways and reduce functional group tolerance, although synthetically attractive protocols for carbon–carbon coupling,^3−8^ carbon–heteroatom bond formation,^9−11^ and halide exchange^12,13^ are known.^14^ Our laboratory has contributed to these efforts and introduced several methods that achieve C–F bond functionalization with a variety of alkyl fluorides under mild conditions.^15−21^

**Scheme 1:**
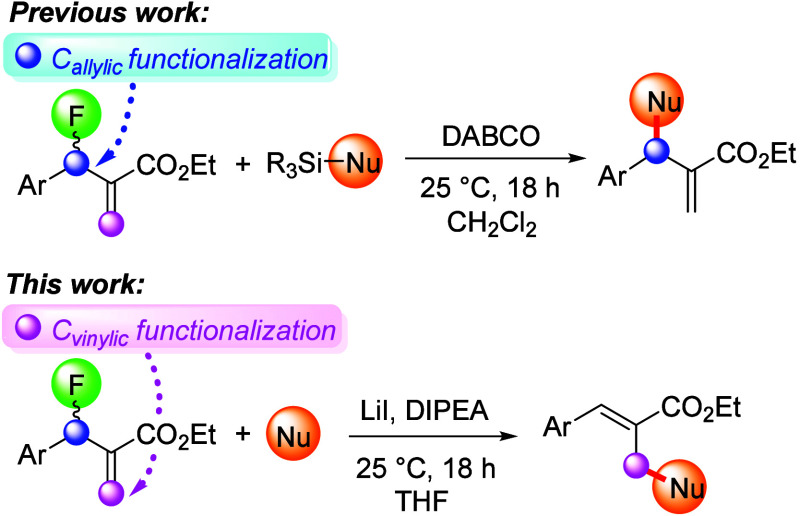
Allylic versus Vinylic Functionalization of MBH Fluorides

We have become increasingly interested in the development of synthetic methodologies that provide unique access to multifunctional compounds and exploit new reactivity patterns, in particular when these complement the outcome of existing reactions. To this end, we noticed that Shibata, Vilotijevic, and co-workers exploited silylated pronucleophiles that typically react at the allylic position in Morita–Baylis–Hillman (MBH) fluorides.^22−31^ By contrast, we envisioned that fluoride displacement might also be possible via attack at the vinylic carbon. Herein, we report that such a pathway by which the fluoride is replaced via formal S_N_2′ reaction can indeed be realized through activation with inexpensive lithium iodide at room temperature (Scheme 1). This protocol affords unprecedented regioselectivity control with carbon, sulfur, and nitrogen nucleophiles producing a variety of compounds in high yields and with good to excellent *E*/*Z* ratios. Moreover, this method allows regioconvergent substitution with isomeric MBH fluorides, which is attributed to the formation of a common (*Z*)-2-(iodomethyl)cinnamate intermediate that is readily consumed in the presence of a nucleophile.

**Scheme 2:**
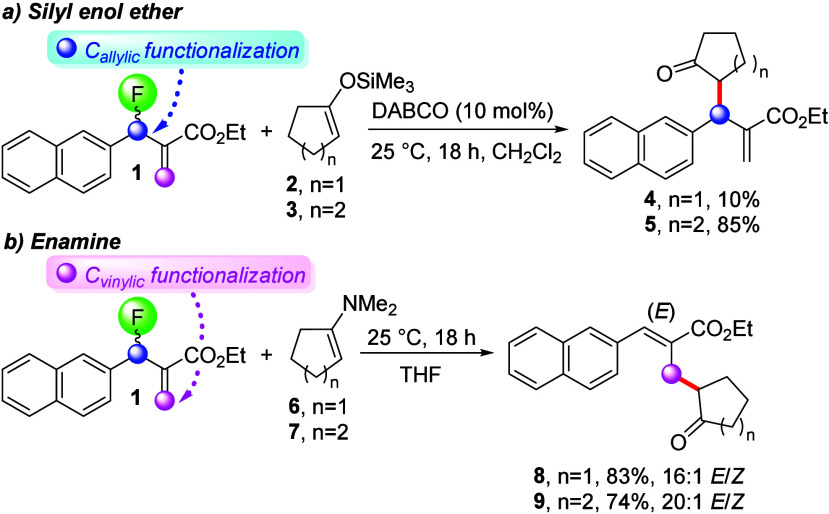
Fluoride Substitution at the Allylic Carbon in the MBH Fluoride **1** with Silyl Enol Ethers versus S_N_2′ Displacement by the Corresponding Enamines

In accordance with previous literature reports, we observed that the MBH fluoride **1** undergoes nucleophilic substitution at the allylic carbon when treated with silyl enol ethers **2** and **3** in the presence of catalytic amounts of DABCO,^26−31^ and we obtained **4** and **5** in 10% and 85% yields, respectively. The low yield of **4** was attributed to the low stability of silyl enol ether **2**, which rapidly decomposed at room temperature. We discovered that employing enamines as nucleophiles switches the regioselectivity to the vinylic carbon resulting in S_N_2′ fluoride displacement (Scheme 2). We were pleased to find that both **6** and **7** afforded **8** and **9** in 74–83% yield and *E*/*Z* ratios of 16:1 and 20:1, respectively.

**Scheme 3:**
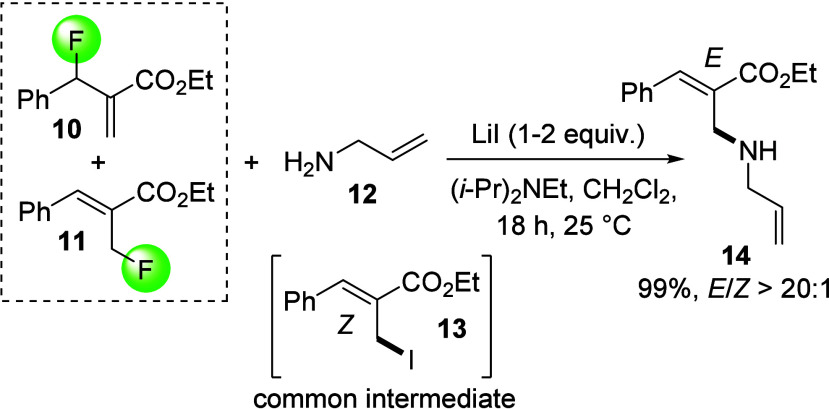
Regioconvergent Addition of Amine **12** to an Isomeric Mixture of the MBH Fluorides **10** and **11** via Iodide Intermediate **13**

When treating a 1:1 isomeric mixture of **10** and **11** with allylamine, **12**, we observed that **10** reacts to the corresponding amine adduct **14** while **11** is not consumed. Further investigation revealed that the addition of LiI facilitates regioconvergent transformation of both **10** and **11** to a single iodide intermediate **13** which reacts quantitatively at room temperature with **12** to **14** exhibiting a high *E*/*Z* ratio of >20:1 (Scheme 3). We were able to isolate **13** to prove its central role in the regioconvergent defluorination pathway (see S1). However, we found that **10** can be directly transformed to **14** via S_N_2′ fluoride displacement in the absence of LiI, but consumption of **11** was not observed unless it was converted in situ to intermediate **13** which undergoes S_N_2 reaction with the amine nucleophile toward the same product. Alternatively, LiI can be replaced with TBAI, YI_3_, or YbI_3_, while TMSI proved less efficient, see SI. This generally points to negligible countercation effects at least when LiI, TBAI, etc., are used. According to Streitwieser,^32^ nucleophilic substitutions at allylic substrates by anionic nucleophiles are generally of the S_N_2 type, or when the S_N_2′ reaction prevails due to steric hindrance it proceeds with anti stereochemistry. The former is observed with the primary fluoride **11**, while **10** is sterically hindered and therefore undergoes anti-S_N_2′ displacement. The diastereoselective conversion of **10** to the *Z* isomer of **13** is in agreement with an S_N_2′ transition state having the phenyl and the ester groups in a coplanar trans conformation according to a study of the reaction between phosphorus nucleophiles and MBH acetates by Georgiadis et al.^33^ This explains the regioconvergent generation of *Z*-**13** from either allylic fluoride. Finally, S_N_2 displacement of the iodide in *Z*-**13** with **12** gives *E*-**14** in high yield and in excellent diastereomeric excess. This method is highly advantageous as it allows the use of both MBH fluoride isomers which are typically obtained as a mixture from their corresponding alcohols and are difficult to separate by column chromatography.

**Scheme 4:**
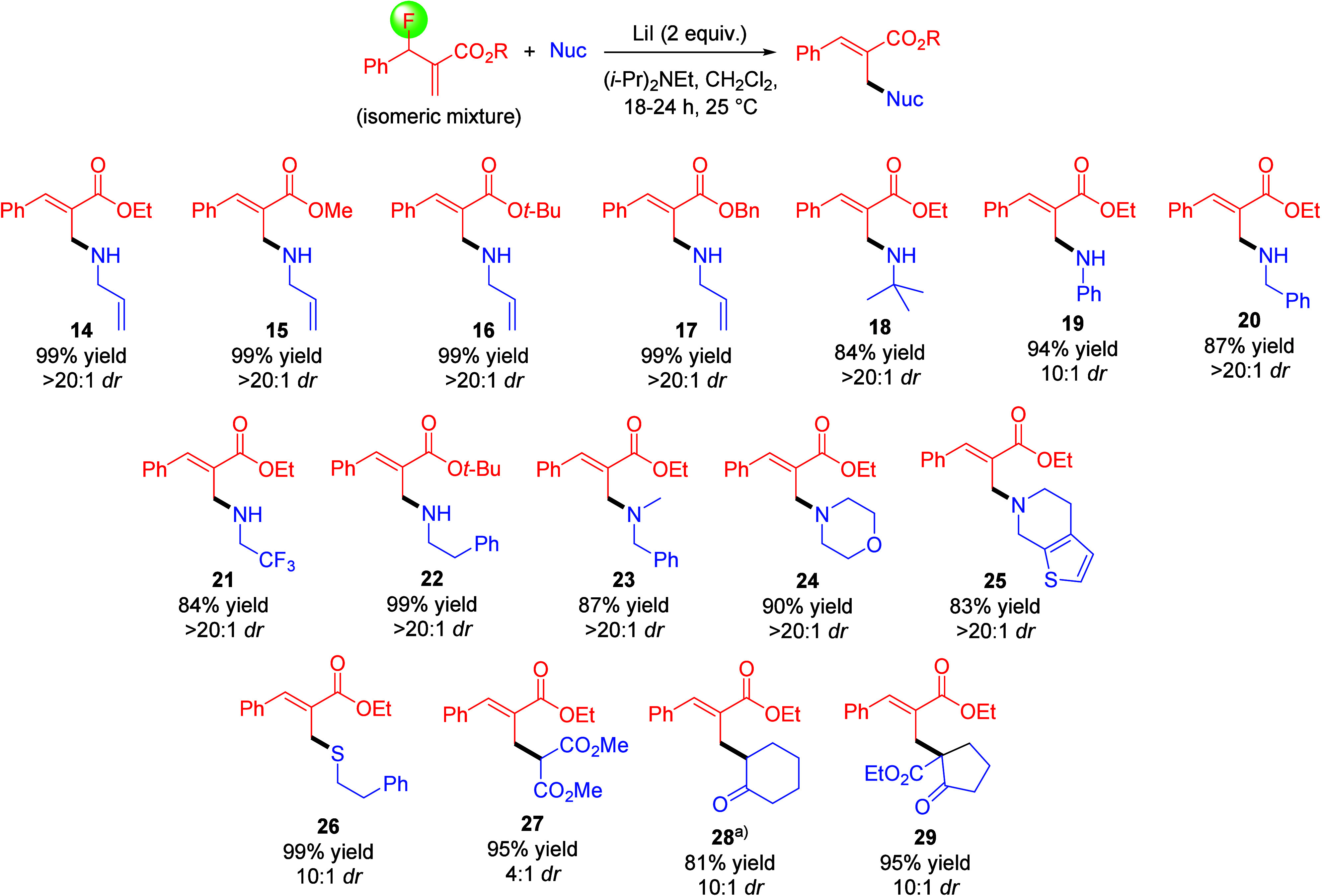
Scope of the Regioconvergent Allylic Substitution Reaction Conditions: MBH fluoride (0.1 mmol), nucleophile (0.2 mmol), LiI (0.2 mmol), DIPEA (0.2 mmol) in anhydrous dichloromethane (0.5 mL). ^a^Prepared from 1-pyrrolidino-1-cyclohexene.

Intrigued by the regioconvergence and high diastereoselectivity of this reaction, we began screening various conditions including base additives, stoichiometry of reactants, and solvents (see SI). We determined that optimal results are obtained with two equivalents of nucleophile, diisopropylethylamine, and LiI in dichloromethane at room temperature. It is noteworthy, however, that only slightly lower yields were obtained with one equivalent of lithium iodide, and the reaction was found to proceed with catalytic amounts, generating **14** in 52% yield as well as 25% of a dialkylation byproduct, see SI. Next, we evaluated the substrate scope under optimized conditions. As shown in Scheme 4, a diverse array of nucleophiles undergoes the desired regioconvergent allylic substitution with isomeric mixtures of MBH fluorides in high yields and good to excellent diastereoselectivity. MBH fluorides with different ester groups gave the corresponding products **14**–**17** in almost quantitative yields and 20:1 dr when treated with allylamine. Overall, amine nucleophiles are well tolerated and give yields ranging from 83% to 99%. The reaction with primary, secondary, and heterocyclic amines all afford the desired products **18** and **20**–**25** in >20:1 dr. A moderate decrease in dr (10:1) was observed when aniline was used in the synthesis of **19**, while yields were not affected. Interestingly, thiols are also tolerated, and we obtained **26** in 99% yield and 10:1 dr. A noticeable drop in the diastereoselectivity was observed with carbon nucleophiles that can, however, be generated in situ with Hünig’s base, thus eliminating the need to prepare enamines. The use of dimethyl malonate, 1-pyrrolidino-1-cyclohexene, and 2-carbethoxycyclopentanone afforded the desired products **27**–**29** with yields ranging from 81% to 95% and dr’s between 4:1 and 10:1. The reaction outcome proved sensitive to the presence of electron-withdrawing and electron-donating groups in the phenyl ring of the MBH fluoride. The 4-cyanophenyl and 4-nitrophenyl derivatives quantitatively converted to the intermediate **13**, but subsequent amination with **12** was not observed even after heating to 50 °C overnight. By contrast, overalkylation to the tertiary amine byproduct could not be controlled with the 3-methoxyphenyl MBH fluoride despite the use of two equivalents of **12**.

**Scheme 5:**
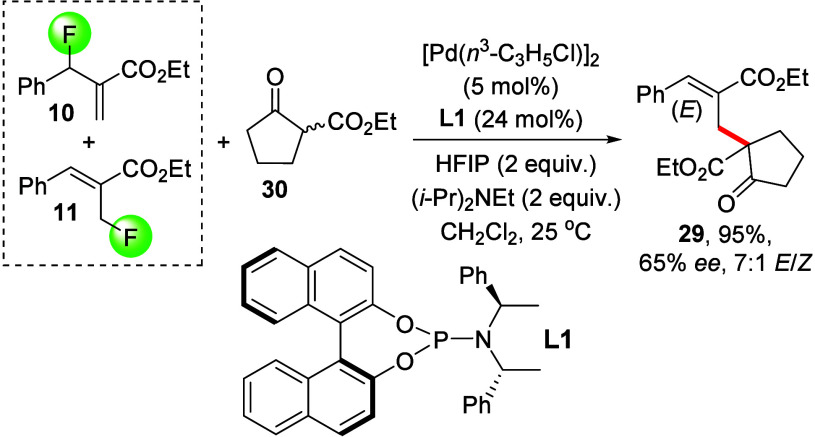
Palladium-Catalyzed Asymmetric Allylic Alkylation Using Ketoester **30** and a Mixture of the Fluorides **10** and **11**

We discovered that HFIP-assisted palladium-catalyzed asymmetric alkylation is also possible and proceeds exclusively at the same carbon atom. Similar to our LiI protocol, we discovered that isomeric mixtures of MBH fluorides **10** and **11** react in a regioconvergent mechanism with the palladium catalyst following fluoride abstraction with HFIP. The comprehensive screening of palladium complexes and reaction conditions revealed that **29** can be obtained in 95% yield, 65% ee, and 10:1 diastereomeric ratio, Scheme 5 and SI.

In conclusion, we have introduced a practical method that allows smooth substitution at the vinylic carbon of MBH fluorides with excellent regioconvergence, yield, and *E/Z* diastereoselectivity with carbon, sulfur, and nitrogen nucleophiles. The use of LiI increases the practicality of this chemistry by enabling regioconvergent substitution of isomeric fluoride mixtures via a common intermediate. This affords multifunctional MBH derivatives in 81–99% yields and high dr’s in most cases. Asymmetric catalytic substitution at the vinylic carbon is also possible, albeit with only moderate enantioselectivity. This method complements previously reported regioselective substitutions of MBH fluorides with silylated pronucleophiles that react at the allylic carbon center. In addition, the necessity to separate *E*/*Z* isomers of the MBH fluoride starting materials is overcome with inexpensive lithium iodide which produces a common allylic iodide intermediate and thus significantly increases overall yields.

## Experimental Section

### General Information

All chemicals and solvents were used as purchased without further purification. The MBH fluorides were synthesized following literature procedures.^22−24,26−29^ NMR spectra were obtained at 400 MHz (^1^H NMR) and 100 MHz (^13^C NMR) in deuterated chloroform or methanol. Chemical shifts are reported in ppm relative to the solvent peak. Reaction products were purified by column chromatography on silica gel (particle size 40–63 μm) as described below.

### General Procedure of the Regioconvergent Substitution of MBH Fluorides with *C*-, *N*-, and *S*-Nucleophiles

A vial was charged with LiI (0.2 mmol), MBH fluoride (0.1 mmol), nucleophile (0.2 mmol), diisopropylethylamine (0.2 mmol), and anhydrous dichloromethane (0.5 mL). The mixture was stirred at room temperature under N_2_ atmosphere for 24 h. The residue of the crude reaction mixture was directly dry loaded onto silica gel and purified by flash chromatography using hexanes–ethyl acetate mixtures as mobile phase as described below.

### General Procedure of the Regioconvergent Substitution of MBH Fluorides with Enamines

A vial was charged with the MBH fluoride (0.1 mmol), enamine (0.1 mmol), and THF (0.5 mL) under nitrogen. The reaction was stirred at room temperature for 18 h. The mixture was quenched with saturated ammonium chloride and extracted with CH_2_Cl_2_, followed by purification of the residue by flash chromatography as described below.

### General Procedure of the Regioconvergent Substitution of MBH Fluorides with Silyl Enol Ethers

A vial was charged with the silyl enol ether (0.1 mmol), DABCO (0.01 mmol), MBH fluoride (0.1 mmol), and anhydrous CH_2_Cl_2_ (0.5 mL) under nitrogen. The reaction was stirred for 18 h. The mixture was quenched with saturated ammonium chloride and extracted with CH_2_Cl_2_, followed by purification of the residue by flash chromatography as described below.

### Asymmetric Allylic Alkylation Procedure

A vial was charged with (*S*)-(+)-(3,5-dioxa-4-phospha-cyclohepta[2,1-a;3,4-a′]dinaphthalen-4-yl)bis[(1*R*)-1-phenylethyl]amine (0.024 mmol, 24 mol %) and [η^3^-C_3_H_5_ClPd]_2_ (0.01 mmol, 5.0 mol %) in anhydrous dichloromethane (0.5 mL). The mixture was stirred at room temperature under N_2_ atmosphere for 1 h. HFIP (0.2 mmol) was added followed by diisopropylethylamine (0.2 mmol), ketoester **30** (0.2 mmol), and the MBH fluoride **10** (0.1 mmol). The resulting mixture was stirred at room temperature for 2 days. The residue of the crude reaction mixture was directly dry loaded onto silica gel and purified by flash chromatography as described below.

### Representative Examples

#### Ethyl 2-(Naphthalen-2-yl(2-oxocyclohexyl)methyl)acrylate (**5**)

Structure **5** was produced as a colorless oil in 85% yield (28.6 mg, 0.09 mmol) from ethyl 2-(fluoro(naphthalen-2-yl)methyl)acrylate (25.8 mg, 0.1 mmol) and (cyclohex-1-en-1yloxy)trimethylsilane (17.0 mg, 0.1 mmol) after 18 h at 25 °C using the protocol provided above and hexanes/EtOAc (95:5) as the mobile phase. The dr was determined as >20:1 by ^1^H NMR analysis. ^1^H NMR (400 MHz, chloroform-*d*) δ 7.79–7.71 (m, 3H), 7.66 (m, 1H), 7.48–7.38 (m, 2H), 7.33 (m, 1H), 6.26 (s, 1H), 5.66 (s, 1H), 4.40 (d, *J* = 11.2 Hz, 1H), 4.14–3.97 (m, 2H), 3.17 (m, 1H), 2.55–2.31 (m, 2H), 2.02 (m, 1H), 1.82–1.48 (m, 4H), 1.39–1.25 (m, 1H), 1.19 (t, *J* = 7.1 Hz, 3H). ^13^C NMR (100 MHz, chloroform-*d*) δ 212.0, 166.7, 143.2, 138.1, 133.4, 132.4, 128.1, 127.8, 127.7, 127.6, 126.5, 126.0, 125.6, 60.8, 54.6, 45.6, 42.5, 33.4, 29.1, 24.6, 14.0. HRMS (ESI-TOF) *m*/*z*: [M + Na]^+^ calcd for C_22_H_24_O_3_Na 359.1619, found 359.1616.

#### Ethyl (*E*)-3-(Naphthalen-2-yl)-2-((2-oxocyclopentyl)methyl)acrylate (**8**)

Compound **8** was formed as a colorless oil in 83% yield (26.8 mg, 0.08 mmol) from ethyl 2-(fluoro(naphthalen-2-yl)methyl)acrylate (25.8 mg, 0.1 mmol) and 1-(cyclopent-1-en-1-yl)pyrrolidine (13.7 mg, 0.1 mmol) after 18 h at 25 °C using the general protocol provided above and hexanes/EtOAc (95:5) as the mobile phase. The dr was determined as 16:1 by ^1^H NMR analysis. ^1^H NMR (400 MHz, chloroform-*d*) δ (m, 5H), 7.52–7.39 (m, 3H), 4.29 (q, *J* = 7.1 Hz, 2H), 3.16 (m, 1H), 2.66 (m, 1H), 2.41 (m, 1H), 2.26 (m, 1H), 2.08 (m, 1H), 1.89 (m, 1H), 1.62 (m, 1H), 1.45 (m, 1H), 1.36 (t, *J* = 7.1 Hz, 3H), 0.83 (m, 1H). ^13^C NMR (100 MHz, chloroform-*d*) δ 219.8, 168.2, 140.0, 133.1, 133.0, 132.9, 131.7, 129.1, 128.4, 128.2, 127.6, 126.8, 126.7, 126.5, 61.0, 48.7, 37.7, 29.7, 27.1, 20.5, 14.3. HRMS (ESI-TOF) *m*/*z*: [M + Na]^+^ calcd for C_21_H_22_O_3_Na 345.1461, found 345.1459.

#### Ethyl (*E*)-2-(Allylamino)methyl)-3-phenyl acrylate (**14**)

Structure **14** was obtained as a colorless oil in 99% yield (24.5 mg, 0.1 mmol) from ethyl 2-(fluoro(phenyl)methyl)acrylate (20.0 mg, 0.1 mmol) and allyl amine (11.0 mg, 0.2 mmol) after 18 h at 25 °C using the general protocol provided above and hexanes/EtOAc (92:8) as the mobile phase. The dr was determined as >20:1 by ^1^H NMR analysis. ^1^H NMR (400 MHz, methanol-*d*_4_) δ 7.83 (s, 1H), 7.44–7.31 (m, 5H), 5.82 (m, 1H), 5.11–5.00 (m, 2H), 4.28 (q, *J* = 7.1 Hz, 2H), 3.59 (s, 2H), 3.17 (ddd, *J* = 6.3, 1.4, 1.4 Hz, 2H), 1.33 (t, *J* = 7.2 Hz, 3H). ^13^C NMR (100 MHz, methanol-*d*_4_) δ 167.8, 141.9, 141.8, 135.8, 134.8, 130.0, 128.9, 127.8, 116.4, 59.8, 50.5, 43.9, 12.7. HRMS (ESI-TOF) *m*/*z*: [M + Na]^+^ calcd for C_15_H_19_NO_2_Na 268.1313, found 268.1308.

This reaction was repeated on a larger scale, and **14** was obtained as a colorless oil in 94% yield (230.4 mg, 0.94 mmol) from ethyl 2-(fluoro(phenyl)methyl)acrylate (208.2 mg, 1.0 mmol) and allyl amine (114.0 mg, 2.0 mmol) after 18 h at 25 °C using the procedure provided above and hexanes/EtOAc (92:8) as the mobile phase. The dr was determined as >20:1 by ^1^H NMR analysis.

#### Ethyl (*Z*)-2-((Phenethylthio)methyl)-3-phenyl acrylate (**26**)

Structure **26** was produced as a colorless oil in 99% yield (32.6 mg, 0.1 mmol) from ethyl 2-(fluoro(phenyl)methyl)acrylate (20.0 mg, 0.1 mmol) and 2-phenylethane-1-thiol (27.6 mg, 0.2 mmol) after 18 h at 25 °C using the general protocol provided above and hexanes/EtOAc (96:4) as the mobile phase. *R_f_* = 0.66 (hexanes/EtOAc, 8:2). The dr was determined as 10:1 by ^1^H NMR analysis. ^1^H NMR (400 MHz, methanol-*d*_4_) δ 7.80 (s, 1H), 7.43–7.30 (m, 5H), 7.28–7.09 (m, 5H), 4.16 (q, *J* = 7.1 Hz, 2H), 3.60 (s, 2H), 2.80–2.67 (m, 4H), 1.23 (t, *J* = 7.1 Hz, 3H). ^13^C NMR (100 MHz, methanol-*d*_4_) δ 167.6, 141.8, 141.6, 139.4, 134.5, 130.6, 129.1, 128.8, 128.4, 128.2, 125.4, 60.72, 49.7, 44.5, 35.0, 13.0. HRMS (ESI-TOF) *m*/*z*: [M + Na]^+^ calcd for C_20_H_22_NO_2_SNa 349.1238, found 349.1235.

#### 3-Ethyl 1,1-Dimethyl (*E*)-4-phenylbut-3-ene-1,1,3-tricarboxylate (**27**)

Structure **27** was produced as a colorless oil in 95% yield (30.2 mg, 0.1 mmol) from ethyl 2-(fluoro(phenyl)methyl)acrylate (20.0 mg, 0.1 mmol) and dimethyl malonate (26.4 mg, 0.2 mmol) after 18 h at at 25 °C using the procedure provided above and hexanes/EtOAc (95:5) as the mobile phase. The dr was determined as 4:1 by ^1^H NMR (400 MHz, methanol-*d*_4_) δ 7.76 (s, 1H), 7.44–7.29 (m, 5H), 4.25 (q, *J* = 7.1 Hz, 2H), 3.74 (t, *J* = 7.8 Hz, 1H), 3.57 (s, 6H), 3.14 (d, *J* = 7.9 Hz, 2H), 1.32 (t, *J* = 7.1 Hz, 3H). ^13^C NMR (100 MHz, methanol-*d*_4_) δ 169.2, 167.5, 141.6, 141.4, 137.4.0, 134.9, 128.8, 128.4, 60.8, 51.4, 50.3, 25.9, 13.1. HRMS (ESI-TOF) *m*/*z*: [M + Na]^+^ calcd for C_17_H_20_O_6_Na 343.1158, found 343.1152.

#### Ethyl (*E*)-1-(2-(Ethoxycarbonyl)-3-phenylallyl)-2-oxocyclopentane-1-carboxylate ((*E*)-**29**)

Compound (*E*)-**29** (32.8 mg, 0.95 mmol) was isolated as a colorless oil in 95% yield from ethyl 2-(fluoro(phenyl)methyl)acrylate (20.8 mg, 0.1 mmol) and ethyl 2-oxocyclopentanecarboxylate (31.2 mg, 0.2 mmol) after 48 h at 25 °C using the general protocol provided above and hexanes/EtOAc (94:6) as the mobile phase. The dr was determined as 10:1 by ^1^H NMR analysis. The ee was determined by HPLC (Chiracel OJ-H, hexanes/*i-*PrOH 98:2, flow rate 1 mL/min, λ = 254 nm) as 65% ee, *t*_R_ (minor) = 43.8 min, *t*_R_ (major) = 59.7 min. ^1^H NMR (400 MHz, methanol-*d*_4_) δ 7.73 (s, 1H), 7.42–7.30 (m, 5H), 4.20 (q, *J* = 7.1 Hz, 2H), 4.06–3.86 (m, 2H), 3.40 (d, *J* = 14.4 Hz, 1H), 2.96 (d, *J* = 14.4 Hz, 1H), 2.41–2.22 (m, 2H), 2.13 (m, 1H), 1.85–1.72 (m, 3H), 1.31 (t, *J* = 7.1 Hz, 3H), 1.11 (t, J = 7.1 Hz, 3H). ^13^C NMR (100 MHz, methanol-*d*_4_) δ 214.3, 170.8, 168.3, 141.5, 135.2, 129.4, 129.1, 128.8, 128.5, 128.1, 61.3, 60.8, 59.5, 36.6, 32.8, 29.8, 18.9, 13.0, 12.7. HRMS (ESI-TOF) *m*/*z*: [M + Na]^+^ calcd for C_20_H_24_O_5_Na 367.1521, found 367.1517.

## Data Availability

The data underlying this study are available in the published article and its Supporting Information.

## Supporting Information Available

Optimization, mechanistic studies, NMR spectra, chiral HPLC results (PDF)
