# Supplementary material for: Regioconvergent Nucleophilic Substitutions with Morita–Baylis–Hillman Fluorides
Source: J Org Chem. 2024 Jul 17;89(15):10998–1002. doi: 10.1021/acs.joc.4c00660 (PMC11301663; doi:10.1021/acs.joc.4c00660)
Supplement: Supplementary file 1 — jo4c00660_si_001.pdf [file jo4c00660_si_001.pdf]

Supporting Information

Regioconvergent Nucleophilic Substitutions with Morita-Baylis-Hillman Fluorides

Jeffrey S. S. K. Formen, Ciarán C. Lynch, Eryn Nelson,
Andi Yuan, Sarah E. Steber and Christian Wolf*

Department of Chemistry, Georgetown University, 37th and O Streets, Washington, DC 20057, USA. Email: cw27@georgetown.edu

Table of Contents

1. General Information S2
2. Optimization Studies S2
3. Product Synthesis and Characterization S5
4. NMR Spectra S24
5. HPLC Chromatograms S61
6. References S62

1. General Information

The commercially available ligands **L1**-**L7**, $[(\eta^3\text{-}C_3H_5)ClPd]_2$, all nucleophiles, reagents and solvents were used as purchased without further purification. The MBH fluorides[1,2] were synthesized following literature procedures using diethylaminosulfur trifluoride (DAST). ***Caution:*** DAST is acutely toxic and an explosion hazard,[3] it should be used with care in a chemical fume hood using proper protective equipment. All MBH fluorides except **1** are known compounds. NMR spectra were obtained at 400 MHz (^{1}H NMR), 100 MHz (^{13}C NMR), and 376 MHz (^{19}F NMR) in $CDCl_3$ or CD_3OD. Chemical shifts are reported in ppm relative to the solvent signal. HR-MS data were obtained using electron spray ionization time-of-flight (ESI-TOF) spectrometry. Reaction products were purified by column chromatography on silica gel (particle size 40-63 μm) as described below.

2. Optimization Studies

Table S1 Optimization of the allylic substitution using MBH fluoride mixtures **10** and **11** with allyl amine **12**.[a]

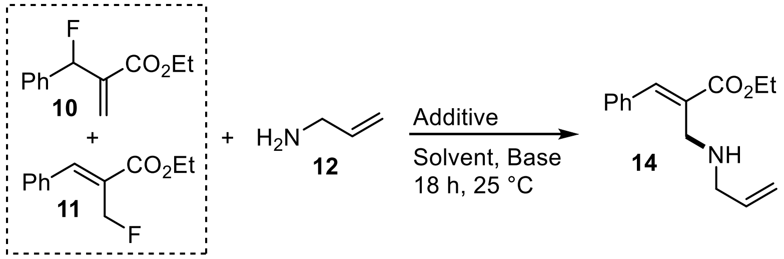

entry	solvent	base	additive	yield[b] [%]	*dr*[c]
1	DCM	DIPEA	LiI	99	>20:1
2	$CHCl_3$	DIPEA	LiI	99	>20:1
3	DCM	DIPEA	-	52	>20:1
4	DCM	N/A	LiI	73	>20:1
5	THF	DIPEA	LiI	74	>20:1
6	DCM	LiOAc	-	70	>20:1
7[d]	DCM	N/A	BCF	60	>20:1
8[e]	DCM	DIPEA	LiI	94	>20:1
9[f]	DCM	DIPEA	LiI	52	>20:1

[a]Reactions were carried out with 1:1 mixture of **10**:**11** (0.1 mmol), allyl amine **12** (0.2 mmol) and additive (0.2 mmol) in 0.5 mL solvent at 25 °C for 18 h under inert atmosphere. [b]Isolated yield. [c]*dr* values were determined using ^{1}H NMR spectroscopy. [d]20 mol% tris(pentafluorophenyl)borane was used as additive. [e] 1.0 eq of LiI was used. [f]10 mol% of LiI was used. Compound **14** was isolated in 52% yield and formation of a dialkylation by-product in 25% yield was determined.

Table S2 Screening of the iodination of the MBH fluoride mixtures **10** and **11**.[a]

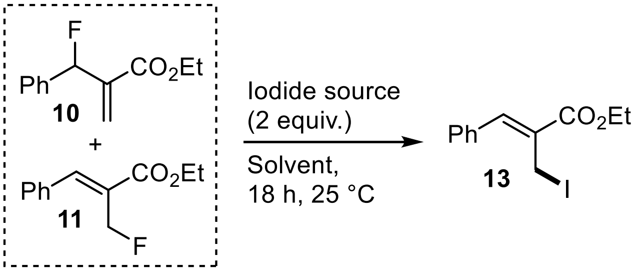

entry	solvent	iodide source	yield[b] [%]	*dr*[c]
1	DCM	LiI	99	>20:1
2	$CHCl_3$	LiI	99	>20:1
3	DCM:THF (1:1)	LiI	99	>20:1
4[d]	Toluene	LiI	20	>20:1
5[e]	DCM	TMSI	67	>20:1
6	DCM	YbI_3·3THF	99	>20:1
7	DCM	YI_3	99	>20:1
8	DCM	TBAI	94	>20:1

[a]Reactions were carried out with 1:1 mixture of **10**:**11** (0.1 mmol) and iodide source (0.2 mmol) in 0.5 mL solvent under inert atmosphere. [b]Isolated yield. [c]*dr* values were determined using ^{1}H NMR spectroscopy. [d] 100 °C. [e]25 °C for 2 h.

Catalytic Asymmetric Allylic Alkylation Study

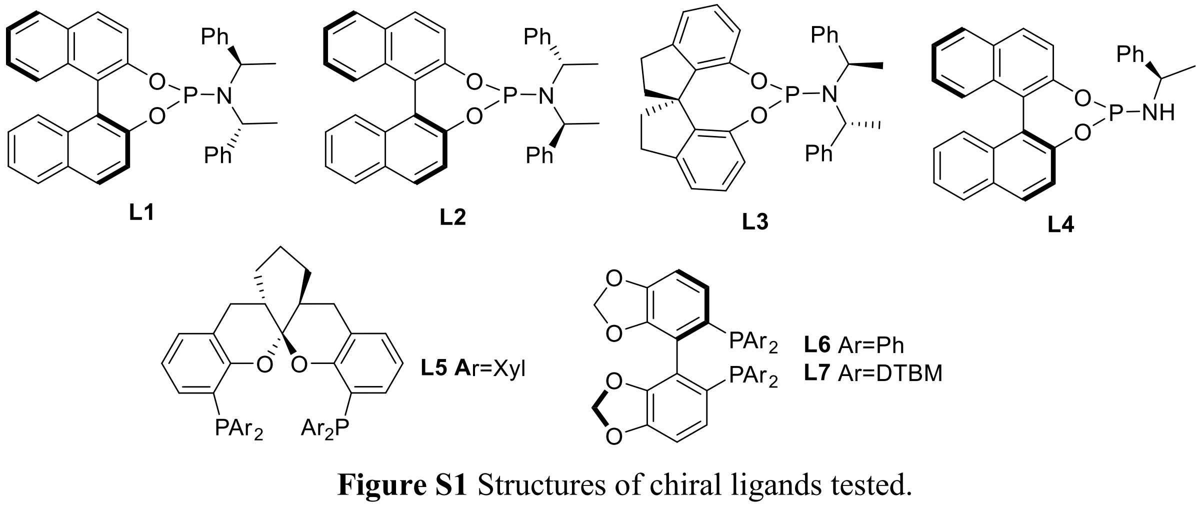

Figure S1 Structures of chiral ligands tested.

Table S3 Optimization of the allylic substitution using MBH fluoride **10** and ketoester **30**.[a]

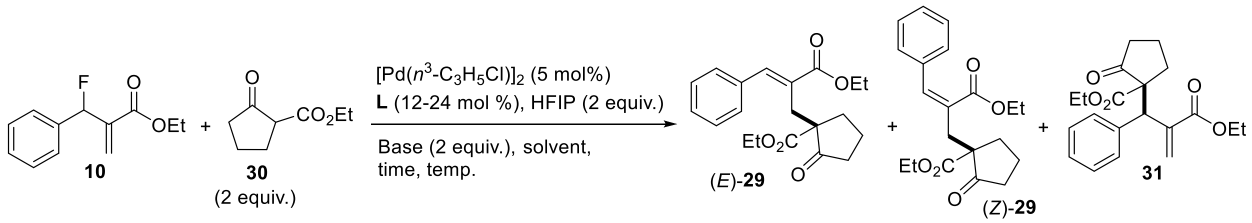

entry	ligand[b]	base	solvent	time [h]	temp [°C]	conversion [%]	(*E*)-29:(*Z*)-29:31[c]	*ee*[d] [%]
1	**L1**	DIPEA	DCM	48	25	>95	10:1:trace	65
2	**L1**	DIPEA	DCM	48	-20	65	6:1:trace	65
3	**L1**	DIPEA	DCM	48	-40	70	6:1:trace	40
4[e]	-	DIPEA	DCM	48	0	15	6:1:trace	-
5	**L1**	DIPEA	$PhCF_3$	48	0	70	8:1:trace	66
6	**L1**	DIPEA	Toluene	48	0	64	9:1:trace	67
7	**L1**	DIPEA	ACN	48	0	97	17:1:trace	62
8	**L1**	DIPEA	1-PrOH	18	0	>95	3:1:trace	75
9[f]	**L1**	DIPEA	1-PrOH	18	0	>95	3:1:trace	77
10	**L1**	DBU	DCM	48	0	94	7:1:trace	16
11	**L1**	Cy_2NMe	DCM	36	0	50	9:1:trace	65
12	**L5**	DIPEA	DCM	48	25	72	12:1:2	-45
13	**L6**	DIPEA	DCM	48	25	83	13:1:0	-65
14	**L7**	DIPEA	DCM	48	25	90	6:1:0	-45
15	**L4**	DIPEA	DCM	48	25	21	6:1:trace	-
16	**L2**	DIPEA	DCM	48	0	45	20:1:trace	43
17	**L3**	DIPEA	DCM	48	0	91	8:1:trace	-20
18[f]	**L1**	DIPEA	DCM	18	0	32	10:1:trace	-66
19[g]	**L1**	DIPEA	DCM	18	0	95	12:1:trace	50
20[e,f]	-	DIPEA	DCM	48	0	-	-	-

[a]Reactions were carried out with **10** (0.1 mmol), ketoester **30** (0.2 mmol), base (0.2 mmol) and HFIP (0.2 mmol) in 0.5 mL solvent under inert atmosphere. [b]Unless otherwise noted, reactions were performed with **L1-L4** (24 mol%) and **L5-L7** (12 mol%). [c]Ratio was determined by ^1H NMR. [d]*ee* values for (*E*)-**29** were determined using a Chiracel OJ-H column and 98:2 hexanes:IPA as mobile phase. [e]No Pd complex. [f]No HFIP. [g]IPA (0.2 mmol) in absence of HFIP.

3. Product Synthesis and Characterization

3.1. Synthesis of the MBH Alcohols[4,i] and Fluorides[1,2,ii]

Into a 20 mL vial were added the aldehyde, DABCO (0.5 equiv.) and acrylate (3 equiv.) and dissolved in H_2O/1,4-dioxane (1:1, v/v, 10.0 M). The mixture was stirred at room temperature for 2–4 days or until judged complete by TLC. The organic layer was separated and the aqueous layer was extracted three times with EtOAc. The combined organic layers were dried over anhydrous Na_2SO_4, filtered and the solvent was removed under reduced pressure The crude product was purified by flash chromatography on silica gel using hexanes-ethyl acetate as mobile phase to give the MBH alcohols as colorless oils. These compounds were used without further purification in the next reaction.

To a solution of an MBH allylic alcohol in dry CH_2Cl_2 (0.33 M), diethylaminosulfur trifluoride (2.0 equiv.) was added while stirring at -78 ºC under nitrogen atmosphere. After 5 minutes, the reaction mixture was brought to room temperature. After stirring for 2 hours at room temperature, the reaction mixture was cooled to 0 ºC and quenched with H_2O. The organic layer was separated and the aqueous layer was extracted three times with CH_2Cl_2. The combined organic layers were dried over anhydrous Na_2SO_4, filtered and the solvent was removed under reduced pressure. The crude product was purified by flash chromatography on silica gel using hexanes-ethyl acetate as mobile phase to give the MBH fluorides as colorless oils. The ^{1}H NMR spectra were identical with the literature reports.

---

[i] Protocol modified from reference 4.

[ii] Protocol modified from references 1 and 2.

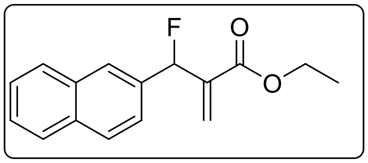

Ethyl 2-(fluoro(naphthalen-2-yl)methyl)acrylate (1). Compound **1** was obtained as a colorless oil in 45% yield (116.1 mg, 1.0 mmol) from ethyl 2-(hydroxy(naphthalen-2-yl)methyl)acrylate (256.3 mg, 1.0 mmol) and diethylaminosulfur trifluoride (322.4 mg, 2.0 mmol) after 2 hours by following the general procedure described above using hexanes/EtOAc (98:2) as the mobile phase. ^{1}H NMR (400 MHz, chloroform-*d*) δ = 7.65-8.05 (m, 4H), 7.39-7.59 (m, 3H), 6.49 (s, 1H), 6.46 (d, $J_{H\text{-}F}$ = 45.7 Hz, 1H), 6.07 (s, 1H), 4.15 (m, 2H), 1.21 (t, J = 7.1 Hz, 3H). ^{13}C{^{1}H} NMR (100 MHz, chloroform-*d*) δ = 164.8 (d, $J_{C\text{-}F}$ = 6.3 Hz), 139.5 (d, $J_{C\text{-}F}$ = 22.6 Hz), 134.8 (d, $J_{C\text{-}F}$ = 20.3 Hz), 133.5 (d, $J_{C\text{-}F}$ = 1.9 Hz), 133.0, 128.3, 128.2 (d, $J_{C\text{-}F}$ = 1.1 Hz), 127.7 (d, $J_{C\text{-}F}$ = 1.1 Hz), 126.8 (d, $J_{C\text{-}F}$ = 6.9 Hz), 126.6 (d, $J_{C\text{-}F}$ = 1.0 Hz), 126.3, 125.8 (d, $J_{C\text{-}F}$ = 8.8 Hz), 124.4 (d, $J_{C\text{-}F}$ = 4.6 Hz), 90.9 (d, $J_{C\text{-}F}$ = 174.0 Hz), 61.0, 14.0 ^{19}F NMR (376 MHz, chloroform-*d*): 171.5 (d, J= 46.3 Hz). HRMS (ESI-TOF) m/z: [M+Na]+ calcd for $C16H_{15}O_2FNa$ 281.0948, found 281.0946.

3.2. Regioselective Allylation with Silyl Enol Ethers

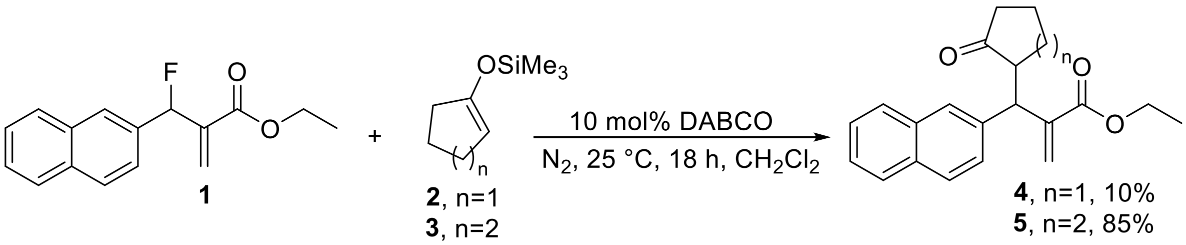

Compounds **4** and **5** were synthesized according to a modified literature protocol.[6] An 8 mL vial was charged with silyl enol ether (0.1 mmol), DABCO (0.01 mmol), MBH fluoride **1** (0.1 mmol), and anhydrous CH_2Cl_2 (0.5 mL) under nitrogen. The reaction was stirred for 18 hours and monitored by TLC. The crude reaction mixture was purified by flash chromatography as described below.

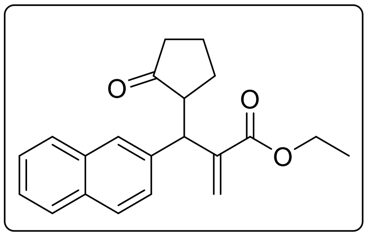

Ethyl 2-(naphthalen-2-yl(2-oxocyclopentyl)methyl)acrylate (4). Compound **4** was obtained as a colorless oil in 10% yield (3.3 mg, 0.01 mmol) from ethyl 2-(fluoro(naphthalen-2-yl)methyl)acrylate (25.8 mg, 0.1 mmol) and (cyclopent-1-en-1yloxy)trimethylsilane (15.6 mg, 0.1 mmol) after 18 hours at room temperature following the general procedure described above using hexanes/EtOAc (95:5) as the mobile phase. The *dr* was determined as 4:1 by ^{1}H NMR analysis. ^{1}H NMR (400 MHz, chloroform-*d*) δ = 7.79 – 7.73 (m, 3H), 7.58 (m, 1H), 7.42 (m, 2H), 7.28 (m, 1H), 6.41 (s, 1H), 5.75 (s, 1H), 4.53 (d, *J* = 6.7 Hz, 1H), 4.12 – 4.00 (m, 2H), 2.80 (m, 1H), 2.23 (m, 1H), 2.06 – 1.89 (m, 2H), 1.85 – 1.65 (m, 2H), 1.51 (m, 1H), 1.11 (t, *J* = 7.1 Hz, 3H). ^{13}C{^{1}H} NMR (100 MHz, chloroform-*d*) δ = δ 218.8, 166.2, 142.7, 137.9, 133.3, 132.4, 128.0, 127.8, 127.5, 127.3, 127.0, 126.0, 125.6, 124.4, 60.8, 51.9, 46.2, 38.2, 27.8, 20.4, 14.0. HRMS (ESI-TOF) m/z: [M+Na]+ calcd for $C_{21}H_{22}O_3Na$ 345.1461, found 345.1460.

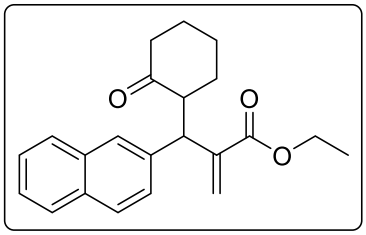

Ethyl 2-(naphthalen-2-yl(2-oxocyclohexyl)methyl)acrylate (5). Compound **5** was obtained as a colorless oil in 85% yield (28.6 mg, 0.09 mmol) from ethyl 2-(fluoro(naphthalen-2-yl)methyl)acrylate (25.8 mg, 0.1 mmol) and (cyclohex-1-en-1yloxy)trimethylsilane (17.0 mg, 0.1 mmol) after 18 hours at room temperature following the general procedure described above using

hexanes/EtOAc (95:5) as the mobile phase. The *dr* was determined as >20:1 by ^{1}H NMR analysis. ^{1}H NMR (400 MHz, chloroform-*d*) δ = 7.79 – 7.71 (m, 3H), 7.66 (m, 1H), 7.48 - 7.38 (m, 2H), 7.33 (m, 1H), 6.26 (s, 1H), 5.66 (s, 1H), 4.40 (d, *J* = 11.2 Hz, 1H), 4.14 - 3.97 (m, 2H), 3.17 (m, 1H), 2.55 - 2.31 (m, 2H), 2.02 (m, 1H), 1.82 – 1.48 (m, 4H), 1.39 – 1.25 (m, 1H), 1.19 (t, *J* = 7.1 Hz, 3H). ^{13}C{^{1}H} NMR (100 MHz, chloroform-*d*) δ = 212.0, 166.7, 143.2, 138.1, 133.4, 132.4, 128.1, 127.8, 127.7, 127.6, 126.5, 126.0, 125.6, 60.8, 54.6, 45.6, 42.5, 33.4, 29.1, 24.6, 14.0. HRMS (ESI-TOF) m/z: [M+Na]+ calcd for $C_{22}H_{24}O_3Na$ 359.1619, found 359.1616.

3.3 Regioselective Allylation with Enamines

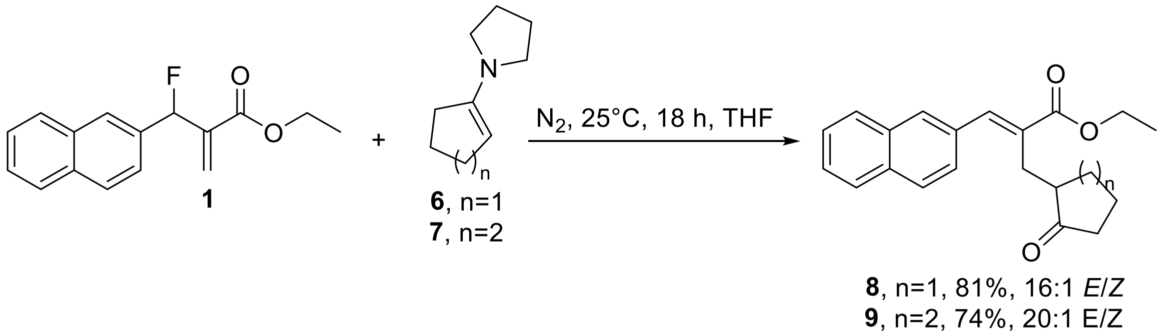

An 8 mL vial was charged with MBH fluoride **1** (0.1 mmol), enamine (0.1 mmol), and THF (0.5 mL) under nitrogen. The reaction was stirred for 18 hours and monitored by TLC. The mixture was washed with saturated ammonium chloride and extracted with CH_2Cl_2, followed by purification of the residue by flash chromatography as described below.

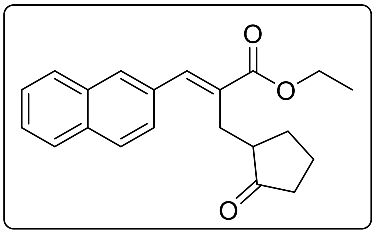

Ethyl (*E*)-3-(naphthalen-2-yl)-2-((2-oxocyclopentyl)methyl)acrylate (8). Compound **8** was obtained as a colorless oil in 83% yield (26.8 mg, 0.08 mmol) from ethyl 2-(fluoro(naphthalen-2-

yl)methyl)acrylate (25.8 mg, 0.1 mmol) and 1-(cyclopent-1-en-1-yl)pyrrolidine (13.7 mg, 0.1 mmol) after 18 hours at room temperature following the general procedure described above using hexanes/EtOAc (95:5) as the mobile phase. The *dr* was determined as 16:1 by ^{1}H NMR analysis. ^{1}H NMR (400 MHz, chloroform-*d*) δ = (m, 5H), 7.52 – 7.39 (m, 3H), 4.29 (q, *J* = 7.1 Hz, 2H), 3.16 (m, 1H), 2.66 (m, 1H), 2.41 (m, 1H), 2.26 (m, 1H), 2.08 (m, 1H), 1.89 (m, 1H), 1.62 (m, 1H), 1.45 (m, 1H), 1.36 (t, *J* = 7.1 Hz, 3H), 0.83 (m, 1H). ^{13}C{^{1}H} NMR (100 MHz, chloroform-*d*) δ = 219.8, 168.2, 140.0, 133.1, 133.0, 132.9, 131.7, 129.1, 128.4, 128.2, 127.6, 126.8, 126.7, 126.5, 61.0, 48.7, 37.7, 29.7, 27.1, 20.5, 14.3. HRMS (ESI-TOF) m/z: [M+Na]+ calcd for $C_{21}H_{22}O_3Na$ 345.1461, found 345.1459.

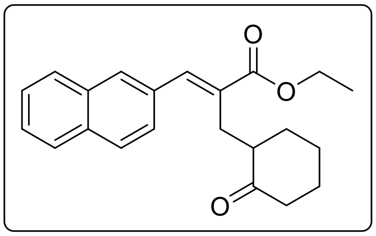

Ethyl (*E*)-3-(naphthalen-2-yl)-2-((2-oxocylcohexyl)methyl)acrylate (9). Compound **9** was obtained as a colorless oil in 74% yield (24.1 mg, 0.07 mmol) from ethyl 2-(fluoro(naphthalen-2-yl)methyl)acrylate (25.8 mg, 0.1 mmol) and 1-(cyclohex-1-en-1-yl)pyrrolidine (15.1 mg, 0.1 mmol) after 18 hours at room temperature following the general procedure described above using hexanes/EtOAc (95:5) as the mobile phase. The *dr* was determined as >20:1 by ^{1}H NMR analysis. ^{1}H NMR (400 MHz, chloroform-*d*) δ = 7.89 (s, 1H), 7.84 – 7.72 (m, 4H), 7.49 – 7.47 (m, 3H), 4.27 (q, *J* = 7.1 Hz, 2H), 3.14 (m, 1H), 2.73 (m, 1H), 2.62 (m, 1H), 2.35 (m, 1H), 2.24 (m, 1H), 2.05 – 1.93 (m, 2H), 1.69 (m, 1H), 1.58 – 1.49 (m, 2H), 1.35 (t, *J*= 7.1 Hz, 3H), 1.19 (m, 1H). ^{13}C{^{1}H} NMR (100 MHz, chloroform-*d*) δ = 212.6, 165.7, 140.4, 133.2, 133.1, 132.9, 131.7,

129.0, 128.3, 128.1, 127.6, 126.6, 126.5, 126.4, 60.9, 50.2, 41.3, 32.5, 27.7, 26.8, 24.9, 14.3. HRMS (ESI-TOF) m/z: [M+Na]+ calcd for $C_{22}H_{24}O_3Na$ 359.1619, found 359.1614.

3.4. Synthesis of Iodide 13

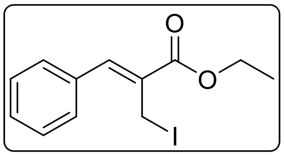

Ethyl (*Z*)-2-(iodomethyl)-3-phenylacrylate (13).[5] Compound **13** was obtained as a colorless oil in 99% yield (31.5 mg, 0.1 mmol) from ethyl 2-(fluoro(phenyl)methyl)acrylate (20.0 mg, 0.1 mmol) and LiI (26.8 mg, 0.2 mmol) after 18 h at room temperature by following the general procedure described above using hexanes/EtOAc (98:2) as the mobile phase. Rf = 0.78 (hexanes/EtOAc, 8:2). The *dr* was determined as >20:1 by ^{1}H NMR analysis. ^{1}H NMR (400 MHz, chloroform-*d*) δ = 7.81 (s, 1H), 7.57 (dd, *J* = 7.7, 1.7 Hz, 2H), 7.50 – 7.43 (m, 2H), 7.41 (m, 1H), 4.41 (q, *J* = 7.1 Hz, 2H), 4.34 (s, 2H), 1.41 (t, *J* = 7.1 Hz, 3H). $^{13}C\{^{1}H\}$ NMR (100 MHz, chloroform-*d*) δ = 166.3, 140.6, 134.9, 130.4, 129.6, 129.3, 129.0, 61.6, 14.4, -0.1.

3.5. Regioconvergent Substitution of MBH Fluorides

An 8 mL vial was charged with LiI (0.2 mmol), MBH fluoride (0.1 mmol), nucleophile (0.2 mmol), DIPEA (0.2 mmol) and anhydrous dichloromethane (0.5 mL). The mixture was stirred at room temperature under N_2 atmosphere for 24 hours and the reaction was monitored by TLC. The crude reaction mixture was directly dry-loaded onto silica gel and purified by flash chromatography using hexanes-ethyl acetate as mobile phase as described below.

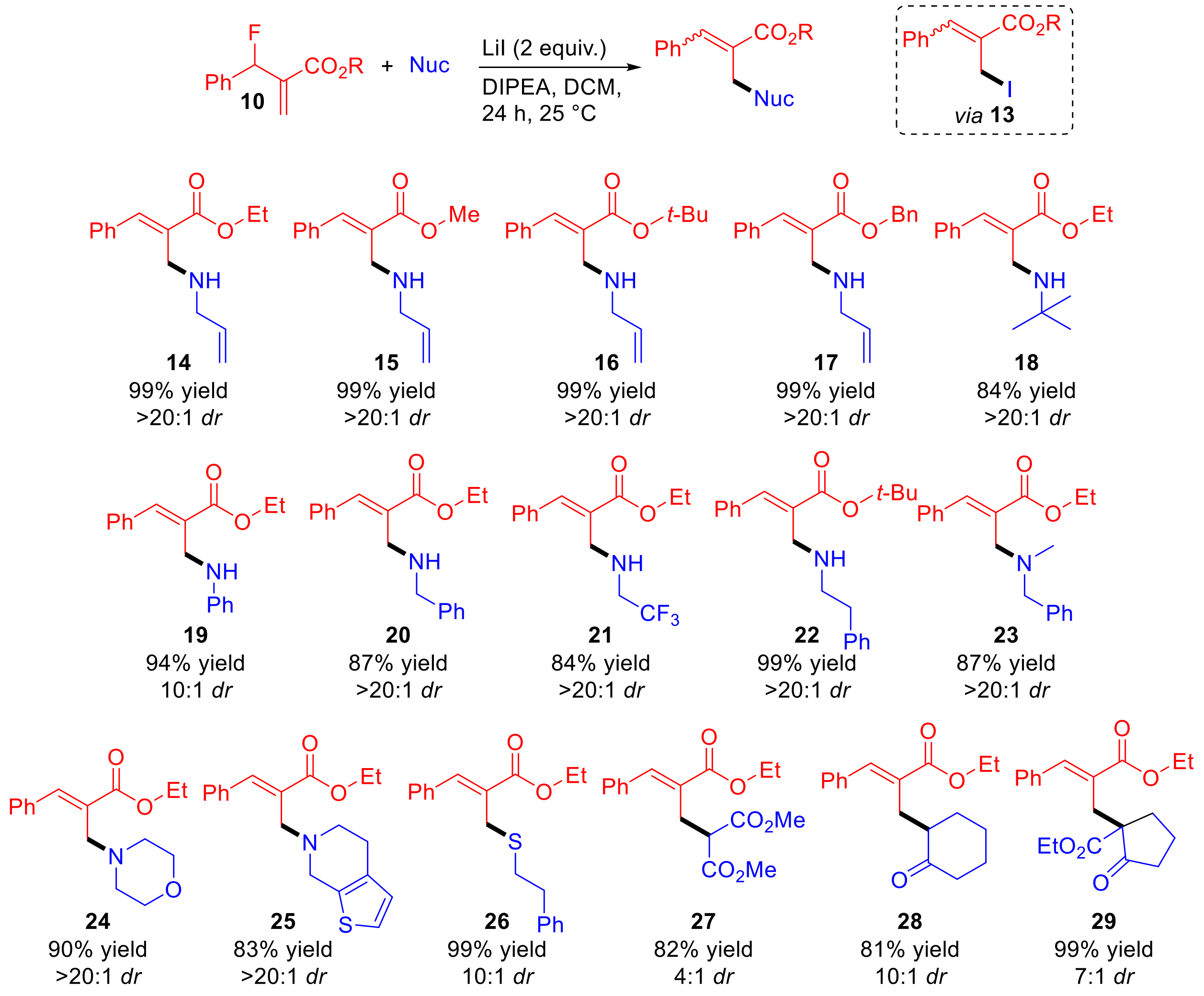

Scheme S1 Substrate scope of the allylic substitution using MBH fluorides and commercially available nucleophiles.

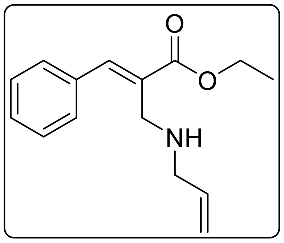

Ethyl (*E*)-2-(allylamino)methyl)-3-phenylacrylate (14). Structure **14** was formed as a colorless oil in 99% yield (24.5 mg, 0.1 mmol) from ethyl 2-(fluoro(phenyl)methyl)acrylate (20.0 mg, 0.1 mmol) and allyl amine (11.0 mg, 0.2 mmol) after 18 hours at 25 ºC using the general protocol provided above and hexanes/EtOAc (92:8) as the mobile phase. Rf = 0.20 (hexanes/EtOAc, 8:2) The *dr* was determined as >20:1 by ^{1}H NMR analysis. ^{1}H NMR (400 MHz, methanol-d$_4$): δ = 7.83 (s, 1H), 7.44-7.31 (m, 5H), 5.82 (m, 1H), 5.11 – 5.00 (m, 2H), 4.28 (q, *J* = 7.1 Hz, 2H), 3.59 (s, 2H), 3.17 (ddd, *J* = 6.3, 1.4, 1.4 Hz, 2H), 1.33 (t, *J* = 7.2 Hz, 3H). ^{13}C NMR (100 MHz, methanol-d$_4$): δ = 167.8, 141.9, 141.8, 135.8, 134.8, 130.0, 128.9, 127.8, 116.4, 59.8, 50.5, 43.9, 12.7. HRMS (ESI-TOF) m/z: [M+Na]+ calcd for $C_{15}H_{19}NO_2Na$ 268.1313, found 268.1308.

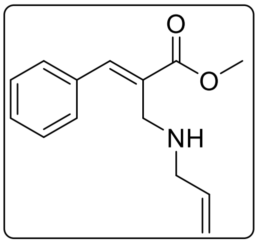

Methyl (*E*)-2-(allylamino)methyl)-3-phenylacrylate (15). Compound **15** was obtained as a colorless oil in 99% yield (23.0 mg, 0.1 mmol) from methyl 2-(fluoro(phenyl)methyl)acrylate (19.4 mg, 0.1 mmol) and allyl amine (11.0 mg, 0.2 mmol) after 18 h at room temperature by following the general procedure described above using hexanes/EtOAc (92:8) as the mobile phase. Rf = 0.14 (hexanes/EtOAc, 8:2). The *dr* was determined as >20:1 by ^{1}H NMR analysis. ^{1}H NMR (400 MHz, chloroform-d): δ = 7.80 (s, 1H), 7.47 (d, *J* = 7.0 Hz, 2H), 7.40 – 7.30 (m, 3H), 5.88 (m,

1H), 5.16 – 5.00 (m, 2H), 3.82 (s, 3H), 3.58 (s, 2H), 3.28 – 3.19 (m, 2H). ^{13}C NMR (100 MHz, chloroform-d): δ = ^{13}C{^{1}H} NMR (100 MHz, cdcl$_3$) δ 168.5, 141.9, 136.6, 135.1, 130.7, 129.6, 128.8, 128.4, 116.1, 52.1, 52.0, 45.2. HRMS (ESI-TOF) m/z: [M+Na]+ calcd for $C_{14}H_{17}NO_2Na$ 254.1157, found 254.1121.

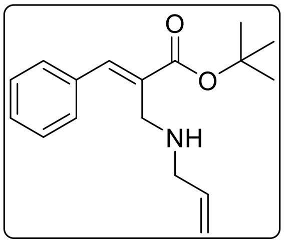

***tert*-Butyl (*E*)-2-(allylamino)methyl)-3-phenylacrylate (16)**. Compound **16** was obtained as a colorless oil in 99% yield (27.3 mg, 0.1 mmol) from *tert*-butyl 2-(fluoro(phenyl)methyl)acrylate (23.6 mg, 0.1 mmol) and allyl amine (11.0 mg, 0.2 mmol) after 18 h at room temperature by following the general procedure described above using hexanes/EtOAc (92:8) as the mobile phase. Rf = 0.62 (hexanes/EtOAc, 1:1). The *dr* was determined as >20:1 by ^{1}H NMR analysis.^{1}H NMR (400 MHz, chloroform-d): δ = 7.70 (s, 1H), 7.45 (d, *J* = 7.0 Hz, 2H), 7.41 – 7.27 (m, 3H), 5.89 (m, 1H), 5.17 – 4.99 (m, 2H), 3.52 (s, 2H), 3.23 (d, *J* = 6.1, 2H), 1.53 (s, 9H). ^{13}C{^{1}H} NMR (100 MHz, chloroform-d): δ = 167.3, 140.8, 136.8, 135.4, 132.4, 129.5, 128.4, 116.1, 81.0, 52.1, 45. 5, 28.2. HRMS (ESI-TOF) m/z: [M+H]+ calcd for $C_{17}H_{24}NO_2$ 274.1802, found 274.1815.

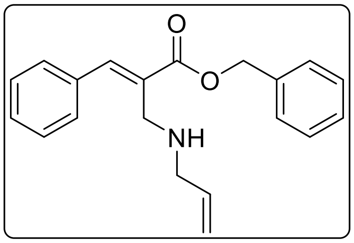

Benzyl (*E*)-2-(allylamino)methyl)-3-phenylacrylate (17). Compound **17** was obtained as a colorless oil in 99% yield (30.7 mg, 0.1 mmol) from benzyl 2-(fluoro(phenyl)methyl)acrylate (27.0 mg, 0.1 mmol) and allyl amine (11.0 mg, 0.2 mmol) after 18 h at room temperature by following the general procedure described above using hexanes/EtOAc (92:8) as the mobile phase. Rf = 0.52 (hexanes/EtOAc, 1:1). The *dr* was determined as >20:1 by ^{1}H NMR analysis. ^{1}H NMR (400 MHz, chloroform-d): δ = 7.84 (s, 1H), 7.47 (d, *J* = 7.5 Hz, 2H), 7.43 – 7.29 (m, 8H), 5.86 (m, 1H), 5.26 (s, 2H), 5.15 – 4.97 (m, 2H), 3.60 (s, 2H), 3.23 (d, *J* = 6.2 Hz, 2H). ^{13}C{^{1}H} NMR (100 MHz, chloroform-d): δ = 167.9, 142.2, 136.6, 136.0, 135.1, 130.7, 129.6, 128.9, 128.6, 128.4, 128.2, 128.1, 116.1, 66.7, 52.1, 45.3. HRMS (ESI-TOF) m/z: [M+Na]+ calcd for $C_{20}H_{21}NO_2Na$ 330.1470, found 330.1484.

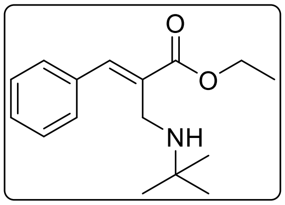

Ethyl (*E*)-2-((*tert*-butylamino)methyl)-3-phenylacrylate (18). Compound **18** was obtained as a colorless oil in 84% yield (22.0 mg, 0.1 mmol) from ethyl 2-(fluoro(phenyl)methyl)acrylate (27.0 mg, 0.1 mmol) and *tert*-butyl amine (14.6 mg, 0.2 mmol) after 18 h at room temperature by following the general procedure described above using hexanes/EtOAc (92:8) as the mobile phase. Rf = 0.25 (hexanes/EtOAc, 8:2). The *dr* was determined as >20:1 by ^{1}H NMR analysis. ^{1}H NMR (400 MHz, chloroform-d): δ = 7.77 (s, 1H), 7.58 (d, *J* = 7.4 Hz, 2H), 7.41 – 7.28 (m, 3H), 4.26 (q, *J* = 7.1 Hz, 2H), 3.52 (s, 2H), 1.33 (t, *J* = 7.1, 3H), 1.14 (s, 9H). ^{13}C{^{1}H} NMR (100 MHz, chloroform-d): δ = 168.2, 141.4, 135.3, 131.4, 129.7, 128.7, 128.3, 60.8, 50.9, 39.7, 28.9, 14.3. HRMS (ESI-TOF) m/z: [M+H]+ calcd for $C_{16}H_{24}NO_2$ 262.1802, found 262.1802.

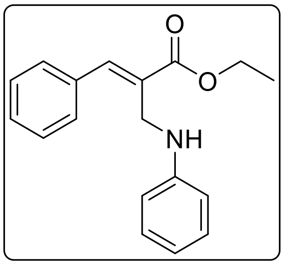

Ethyl (*E*)-3-phenyl-2-((phenylamino)methyl)acrylate (19). Compound **19** was obtained as a white oil in 94% yield (26.4 mg, 0.09 mmol), from ethyl 2-(fluoro(phenyl)methyl)acrylate (20.0 mg, 0.1 mmol) and aniline (18.6 mg, 0.2 mmol) after 18 h at room temperature by following the general procedure described above using hexanes/EtOAc (96:4) as the mobile phase. Rf = 0.44 (hexanes/EtOAc, 8:2). The *dr* was determined as 10:1 by ^{1}H NMR analysis. ^{1}H NMR (400 MHz, chloroform-d): δ = 7.86 (s, 1H), 7.47 – 7.32 (m, 5H), 7.16 – 7.09 (m, 2H), 6.70 (dd, *J* = 7.3, 0.7 Hz, 1H), 6.51 (d, *J* = 8.0 Hz, 2H), 4.28 (q, *J* = 7.1 Hz, 2H), 4.12 (s, 2H), 1.33 (t, *J* = 7.1 Hz, 3H). ^{13}C{^{1}H} NMR (100 MHz, chloroform-d): δ = 167.7, 147.8, 142.5, 134.9, 129.6, 129.5, 129.1, 128.8, 128.6, 117.8, 113.4, 61.1, 41.0, 14.3. HRMS (ESI-TOF) m/z: [M+Na]+ calcd for $C_{18}H_{19}NO_2Na$ 304.1313, found 304.1307.

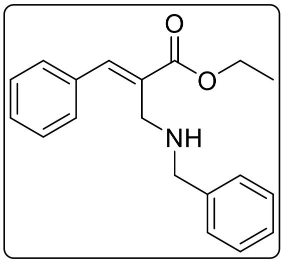

Ethyl (*E*)-2-((benzylamino)methyl)-3-phenylacrylate (20). Compound **20** was obtained as a colorless oil in 87% yield (26.9 mg, 0.09 mmol) from ethyl 2-(fluoro(phenyl)methyl)acrylate (20.0 mg, 0.1 mmol) and *N*-benzyl amine (21.4 mg, 0.2 mmol) after 18 h at room temperature by

following the general procedure described above using hexanes/EtOAc (92:8) as the mobile phase. Rf = 0.36 (hexanes/EtOAc, 8:2). The *dr* was determined as >20:1 by ^{1}H NMR analysis. ^{1}H NMR (400 MHz, chloroform-d): δ = 7.79 (s, 1H), 7.43 – 7.40 (m, 2H), 7.34 – 7.19 (m, 8H), 4.27 (q, *J* = 7.1 Hz, 2H), 3.80 (s, 2H), 3.58 (s, 2H), 1.33 (t, *J* = 7.1 Hz, 3H). $^{13}C\{^{1}H\}$ NMR (100 MHz, chloroform-d): δ = 168.1, 141.7, 140.0, 135.1, 130.8, 129.6, 128.7, 128.4, 128.4, 128.3, 126.9, 60.9, 53.7, 45.4, 14.3. HRMS (ESI-TOF) m/z: HRMS (ESI-TOF) m/z: [M+H]+ calcd for $C_{19}H_{21}NO_2H$ 296.1648, found 296.1648.

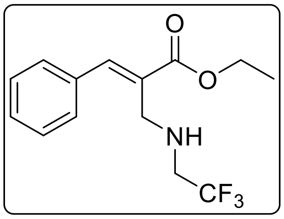

Ethyl (*E*)-3-phenyl-2-(((2,2,2-trifluoromethyl)amino)methyl) acrylate (21). Compound **21** was obtained as a colorless oil in 84% yield (26.9 mg, 0.09 mmol) from ethyl 2-(fluoro(phenyl)methyl)acrylate (20.0 mg, 0.1 mmol) and 2,2,2-trifluoroethane-1-amine (19.8 mg, 0.2 mmol) after 18 h at room temperature by following the general procedure described above using hexanes/EtOAc (97:3) as the mobile phase. Rf = 0.72 (hexanes/EtOAc, 8:2). The *dr* was determined as >20:1 by ^{1}H NMR analysis. ^{1}H NMR (400 MHz, chloroform-d): δ = 7.83 (s, 1H), 7.48 – 7.45 (m, 2H), 7.41 – 7.33 (m, 3H), 4.29 (q, *J* = 7.2 Hz, 2H), 3.70 (s, 2H), 3.18 (q, *J* = 9.4 Hz, 2H), 1.35 (d, *J* = 7.2 Hz, 3H). $^{13}C\{^{1}H\}$ NMR (100 MHz, chloroform-d): δ = 167.8. 142.4, 134.8, 130.0, 129.4, 129.0, 128.5, 126.0 (q, *J* = 279 Hz), 61.1, 50.1 (q, *J* = 31.7 Hz), 45.8, 14.2. ^{19}F NMR (376 MHz, chloroform-d) δ = -71.65 (t, *J* = 9.4 Hz). HRMS (ESI-TOF) m/z: [M+Na]+ calcd for $C_{14}H_{16}F_3NO_2Na$ 310.1031, found 310.1038.

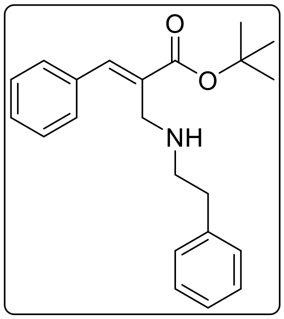

***tert*-Butyl (*E*)-2-((phenethylamino)methyl)-3-phenylacrylate (22).** Compound **22** was obtained as a colorless oil in 99% yield (30.9 mg, 0.1 mmol) from *tert*-butyl 2-(fluoro(phenyl)methyl)acrylate (20.0 mg, 0.1 mmol) and 2-phenylethan-1-amine (24.2 mg, 0.2 mmol) after 18 h at room temperature by following the general procedure described above using hexanes/EtOAc (92:8) as the mobile phase. Rf = 0.32 (hexanes/EtOAc, 8:2). The *dr* was determined as >20:1 by ^{1}H NMR analysis. ^{1}H NMR (400 MHz, chloroform-d): δ = 7.69 (s, 1H), 7.45 – 7.39 (m, 2H), 7.39 – 7.23 (m, 5H), 7.21 – 7.14 (m, 3H), 3.56 (s, 2H), 2.88 – 2.84 (m, 2H), 2.80 – 2.75 (m, 2H), 1.46 (s, 9H). ^{13}C{^{1}H} NMR (100 MHz, chloroform-d): δ = 167.2, 140.8, 140.1, 135.4, 132.3, 129.4, 128.7, 128.5, 128.4, 126.0, 80.9, 50.6, 45.9, 36.3, 28.1. HRMS (ESI-TOF) m/z: [M+Na]+ calcd for $C_{22}H_{27}NO_2Na$ 360.1939, found 360.1934.

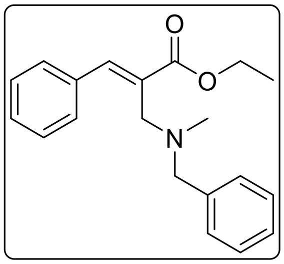

Ethyl (*E*)-2-((benzyl(methyl)amino)methyl)-3-phenylacrylate (23). Compound **23** was obtained as a colorless oil in 87% yield (26.9 mg, 0.09 mmol) from ethyl 2-(fluoro(phenyl)methyl)acrylate (20.0 mg, 0.1 mmol) and *N*-methylbenzylamine (31.5 mg, 0.2

mmol) after 18 h at room temperature by following the general procedure described above using hexanes/EtOAc (92:8) as the mobile phase. Rf = 0.75 (hexanes/EtOAc, 1:1). The *dr* was determined as >20:1 by ^{1}H NMR analysis. ^{1}H NMR (400 MHz, chloroform-d): δ = 7.81 (s, 1H), 7.67 (d, *J* = 7.3 Hz, 2H), 7.41 – 7.18 (m, 8H), 4.28 (q, *J* = 7.1 Hz, 2H), 3.50 (s, 2H), 3.45 (s, 2H), 2.13 (s, 3H), 1.34 (t, *J* = 7.1 Hz, 3H). ^{13}C{^{1}H} NMR (100 MHz, chloroform-d): δ = 168.8, 142. 6, 139.3, 135.4, 130.6, 130.5, 129.0, 128.8, 128.3, 128.1, 126.9, 61.9, 60.9, 53.1, 41.5, 14.3. HRMS (ESI-TOF) m/z: [M+Na]+ calcd for $C_{20}H_{23}NO_2Na$ 332.1626, found 332.1615.

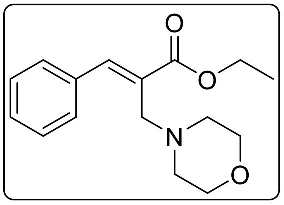

Ethyl (*E*)-2-(morpholinomethyl)-3-phenylacrylate (24). Compound **24** was obtained as a colorless oil in 90% yield (24.8 mg, 0.1 mmol) from ethyl 2-(fluoro(phenyl)methyl)acrylate (27.0 mg, 0.1 mmol) and morpholine (17.4 mg, 0.2 mmol) after 18 h at room temperature by following the general procedure described above using hexanes/EtOAc (96:4) as the mobile phase. Rf = 0.44 (hexanes/EtOAc, 8:2). The *dr* was determined as >20:1 by ^{1}H NMR analysis.^{1}H NMR (400 MHz, chloroform-d): δ = 7.84 (s, 1H), 7.63 (d, *J* = 7.3 Hz, 2H), 7.42 – 7.30 (m, 3H), 4.27 (q, *J* = 7.2 Hz, 2H), 3.68 – 3.66 (m, 4H), 3.34 (s, 2H), 2.52 – 2.41 (m, 4H), 1.34 (t, *J* = 7.1 Hz, 3H). ^{13}C{^{1}H} NMR (100 MHz, chloroform-d): δ = 168.5, 143.1, 135.4, 130.3, 129.6, 128.9, 128.3, 67.1, 60.9, 53.7, 53.1, 14.3. HRMS (ESI-TOF) m/z: [M+Na]+ calcd for $C_{16}H_{21}NO_3Na$ 298.1419, found 298.1389.

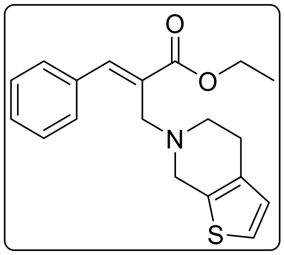

Ethyl (*E*)-2-((4,7-dihydrothieno[2,3-*c*]pyridine-6(*5H*)-yl)methyl)-3-phenylacrylate (25). Compound **25** was obtained as a colorless oil in 83% yield (99.5 mg, 0.08 mmol) from ethyl 2-(fluoro(phenyl)methyl)acrylate (20.0 mg, 0.1 mmol) and 4,5,6,7-tetrahydrothieno[2,3-*c*]pyridine hydrochloride (35.1 mg, 0.2 mmol) after 18 h at room temperature by following the general procedure described above using hexanes/EtOAc (98:2) as the mobile phase. Rf = 0.67 (hexanes/EtOAc, 8:2). The *dr* was determined as >20:1 by ^{1}H NMR analysis. ^{1}H NMR (400 MHz, chloroform-d): δ 7.87 (s, 1H), 7.67 – 7.63 (m 2H), 7.42 – 7.29 (m, 3H), 7.04 (d, J = 5.1 Hz, 1H), 6.69 (d, J = 5.1 Hz, 1H), 4.29 (q, J = 7.2, 2H), 3.59 (s, 2H), 3.55 (s, 2H), 2.86 – 2.79 (m, 4H), 1.35 (t, J = 7.2 Hz, 3H). ^{13}C{^{1}H} NMR (100 MHz, chloroform-d): δ = 168.6, 143.1, 135.4, 134.0, 133.4, 130.4, 130.1, 128.9, 128.4, 125.3, 122.4, 61.3, 52.6, 52.5, 50.2, 25.5, 14.3. HRMS (ESI-TOF) m/z: [M+H]+ calcd for $C_{19}H_{21}NO_2SH$ 328.1367, found 328.1367.

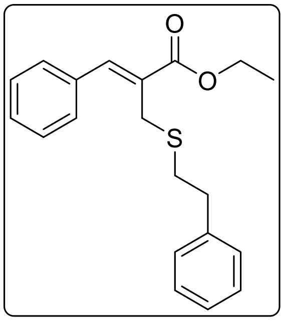

Ethyl (*Z*)-2-((phenethylthio)methyl)-3-phenylacrylate (26). Structure **26** was produced as a colorless oil in 99% yield (32.6 mg, 0.1 mmol), from ethyl 2-(fluoro(phenyl)methyl)acrylate (20.0

mg, 0.1 mmol) and 2-phenylathane-1-thiol (27.6 mg, 0.2 mmol) after 18 hours at 25 °C using the general protocol provided above and hexanes/EtOAc (96:4) as the mobile phase. Rf = 0.66 (hexanes/EtOAc, 8:2). The *dr* was determined as 10:1 by ^{1}H NMR analysis. ^{1}H NMR (400 MHz, methanol-d_4): δ = 7.80 (s, 1H), 7.43 – 7.30 (m, 5H), 7.28 – 7.09 (m, 5H), 4.16 (q, *J* = 7.1 Hz, 2H), 3.60 (s, 2H), 2.80 – 2.67 (m, 4H), 1.23 (t, *J* = 7.1 Hz, 3H). ^{13}C NMR (100 MHz, methanol-d_4): δ = 167.6, 141.8, 141.6, 139.4, 134.5, 130.6, 129.1, 128.8, 128.4, 128.2, 125.4, 60.72, 49.7, 44.5, 35.0, 13.0. HRMS (ESI-TOF) m/z: [M+Na]+ calcd for $C_{20}H_{22}O_2SNa$ 349.1233, found 349.1235.

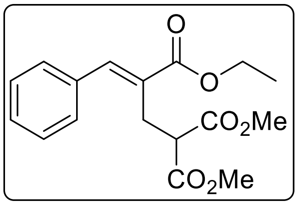

3-Ethyl 1,1-dimethyl (*E*)-4-phenylbut-3-ene-1,1,3-tricarboxylate (27). Structure **27** was isolated as a colorless oil in 95% yield (30.2 mg, 0.1 mmol), from ethyl 2-(fluoro(phenyl)methyl)acrylate (20.0 mg, 0.1 mmol) and dimethyl malonate (26.4 mg, 0.2 mmol) after 18 hours at 25 °C using the general protocol provided above and hexanes/EtOAc (95:5) as the mobile phase. Rf = 0.62 (hexanes/EtOAc, 8:2). The *dr* was determined as 4:1 by ^{1}H NMR analysis. ^{1}H NMR (400 MHz, methanol-d_4): δ = 7.76 (s, 1H), 7.44-7.29 (m, 5H), 4.25 (q, *J* = 7.1 Hz, 2H), 3.74 (t, *J* = 7.8 Hz, 1H), 3.57 (s, 6H), 3.14 (d, *J* = 7.9 Hz, 2H), 1.32 (t, *J* = 7.1 Hz, 3H). ^{13}C NMR (100 MHz, methanol-d_4): δ = 169.2, 167.5, 141.6, 141.4, 137.4.0, 134.9, 128.8, 128.4, 60.8, 51.4, 50.3, 25.9, 13.1. HRMS (ESI-TOF) m/z: [M+Na]+ calcd for $C_{17}H_{20}O_6Na$ 343.1158, found 343.1152.

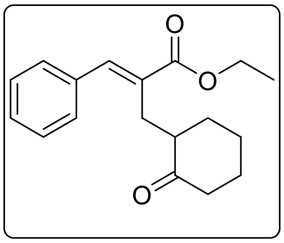

Ethyl (*E*)-2-((2-oxocyclohexyl)methyl)-3-phenylacrylate (28). Compound **28** was obtained as a colorless oil in 81% yield (23.2 mg, 0.08 mmol) from ethyl 2-(fluoro(phenyl)methyl)acrylate (20.0 mg, 0.1 mmol) and 1-pyrrolidino-1-cyclohexene (30.3 mg, 0.2 mmol) after 18 h at room temperature by following the general procedure described above using hexanes/EtOAc (94:6) as the mobile phase. Rf = 0.47 (hexanes/EtOAc, 1:1). The *dr* was determined as 10:1 by ^{1}H NMR analysis. ^{1}H NMR (400 MHz, chloroform-d): δ = 7.73 (s, 1H), 7.40 – 7.26 (m, 5H), 4.24 (q, *J* = 7.1 Hz, 2H), 3.03 (m, 1H), 2.68 – 2.54 (m, 2H), 2.40 – 2.17 (m, 3H), 2.05 – 1.92 (m, 2H), 1.62 – 1.47 (m, 3H), 1.32 (t, *J* = 7.1 Hz, 3H). ^{13}C{^{1}H} NMR (100 MHz, chloroform-d): δ = 212.2, 168.3, 140.4, 135.7, 131.5, 129.1, 128.5, 128.2, 60.8, 49.7, 41.8, 33.1, 28.5, 26.7, 24.9, 14.3. HRMS (ESI-TOF) m/z: [M+Na]+ calcd for $C_{18}H_{22}O_3Na$ 309.1461, found 309.1462.

Asymmetric Allylic Alkylation Procedure

An 8 mL vial was charged with (*S*)-(+)-(3,5-dioxa-4-phospha-cyclohepta[2,1-a;3,4-a']dinaphthalen-4-yl)bis[(1*R*)-1-phenylethyl]amine (0.024 mmol, 24 mol%) and [η^3-C_3H_5ClPd]$_2$ (0.01 mmol, 5 mol%) in anhydrous dichloromethane (0.5 mL). The mixture was stirred at room temperature under N_2 atmosphere for 1 hour. HFIP (0.2 mmol) was added followed by DIPEA (0.2 mmol), the ketoester (0.2 mmol) and the MBH fluoride (0.1 mmol). The resulting mixture

was stirred at room temperature for 2 days and the reaction was monitored by TLC. The crude reaction mixture was directly dry-loaded onto silica gel and purified by flash chromatography using hexanes-ethyl acetate as mobile phase as described below.

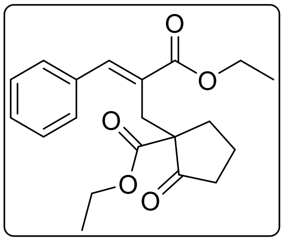

Ethyl (*E*)-1-(2-(ethoxycarbonyl)-3-phenylallyl)-2-oxocyclopentane-1-carboxylate ((*E*)-29).

Structure (*E*)-**31** (32.8 mg, 0.95 mmol) was produced as a colorless oil in 95% yield from ethyl 2-(fluoro(phenyl)methyl)acrylate (20.8 mg, 0.1 mmol) and ethyl 2-oxocyclopentanecarboxylate (31.2 mg, 0.2 mmol) after 48 hours at 25 ºC using the protocol provided above and hexanes/EtOAc (94:6) as the mobile phase. Rf = 0.56 (hexanes/EtOAc, 8:2). The *dr* was determined as 7:1 by ^{1}H NMR analysis. The *ee* was determined by HPLC (Chiracel OJ-H, hexanes/*i*-PrOH 98:2, flow rate 1 mL/min, λ = 254 nm) as 65% *ee*, t_R (minor) = 43.8 min, t_R (major) = 59.7 min; ^{1}H NMR (400 MHz, methanol-d_4): δ = 7.73 (s, 1H), 7.42 – 7.30 (m, 5H), 4.20 (q, *J* = 7.1 Hz, 2H), 4.06-3.86 (m, 2H), 3.40 (d, *J* = 14.4 Hz, 1H), 2.96 (d, *J* = 14.4 Hz, 1H), 2.41 – 2.22 (m, 2H), 2.13 (m, 1H), 1.85 – 1.72 (m, 3H), 1.31 (t, *J* = 7.1 Hz, 3H), 1.11 (t, J = 7.1 Hz, 3H). ^{13}C NMR (100 MHz, methanol-d_4): δ = 214.3, 170.8, 168.3, 141.5, 135.2, 129.4, 129.1, 128.8, 128.5, 128.1, 61.3, 60.8, 59.5, 36.6, 32.8, 29.8, 18.9, 13.0, 12.7. HRMS (ESI-TOF) m/z: [M+Na]+ calcd for $C_{20}H_{24}O_5Na$ 367.1521, found 367.1517.

3.6. Large scale regioconvergent functionalization of MBH fluorides

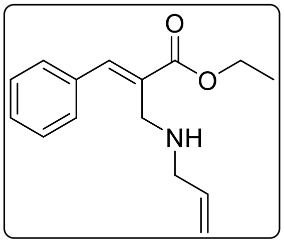

Ethyl (*E*)-2-(allylamino)methyl)-3-phenylacrylate (14). Structure **14** was isolated as a colorless oil in 94% yield (230.4 mg, 0.94 mmol) from ethyl 2-(fluoro(phenyl)methyl)acrylate (208.2 mg, 1.0 mmol) and allyl amine (114.0 mg, 2.0 mmol) after 18 hours at 25 ºC using the general protocol provided above and hexanes/EtOAc (92:8) as the mobile phase. The *dr* was determined as >20:1 by ^{1}H NMR analysis. ^{1}H NMR (400 MHz, methanol-d$_4$): δ = 7.83 (s, 1H), 7.44-7.31 (m, 5H), 5.82 (m, 1H), 5.11 – 5.00 (m, 2H), 4.28 (q, *J* = 7.1 Hz, 2H), 3.59 (s, 2H), 3.17 (ddd, *J* = 6.3, 1.4, 1.4 Hz, 2H), 1.33 (t, *J* = 7.2 Hz, 3H). ^{13}C NMR (100 MHz, methanol-d$_4$): δ = 167.8, 141.9, 141.8, 135.8, 134.8, 130.0, 128.9, 127.8, 116.4, 59.8, 50.5, 43.9, 12.7. HRMS (ESI-TOF) m/z: [M+Na]+ calcd for $C_{15}H_{19}NO_2Na$ 268.1313, found 268.1308.

4. NMR spectra

Figure S2. ^{1}H NMR spectrum of Ethyl 2-(naphthalen-2-yl(2-oxocyclopentyl)methyl)acrylate (**4**) (400 MHz, chloroform-d). (*dr* = 4:1)

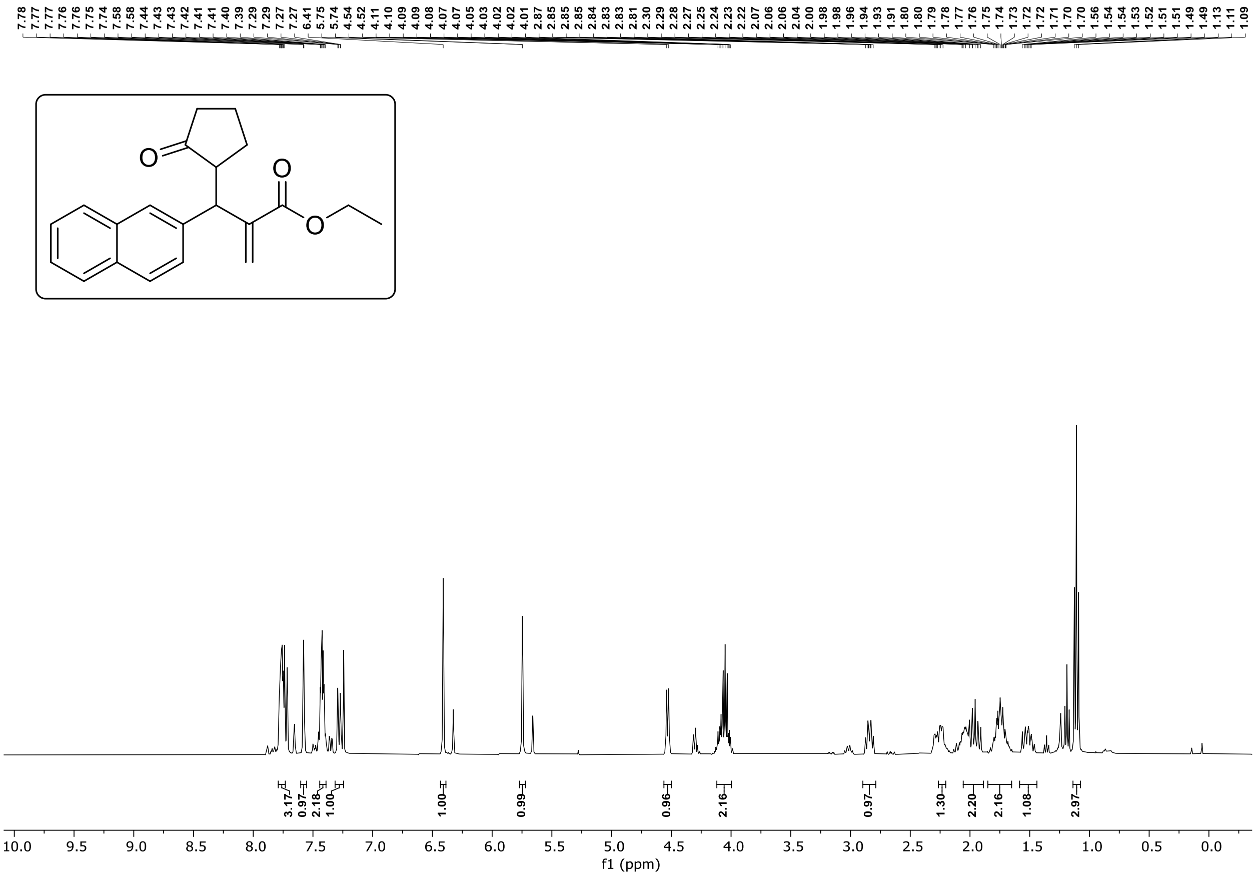

Figure S3. $^{13}C\{^1H\}$ NMR spectrum of Ethyl 2-(naphthalen-2-yl(2-oxocyclopentyl)methyl)acrylate (**4**). (100 MHz, chloroform-d) (*dr* = 4:1)

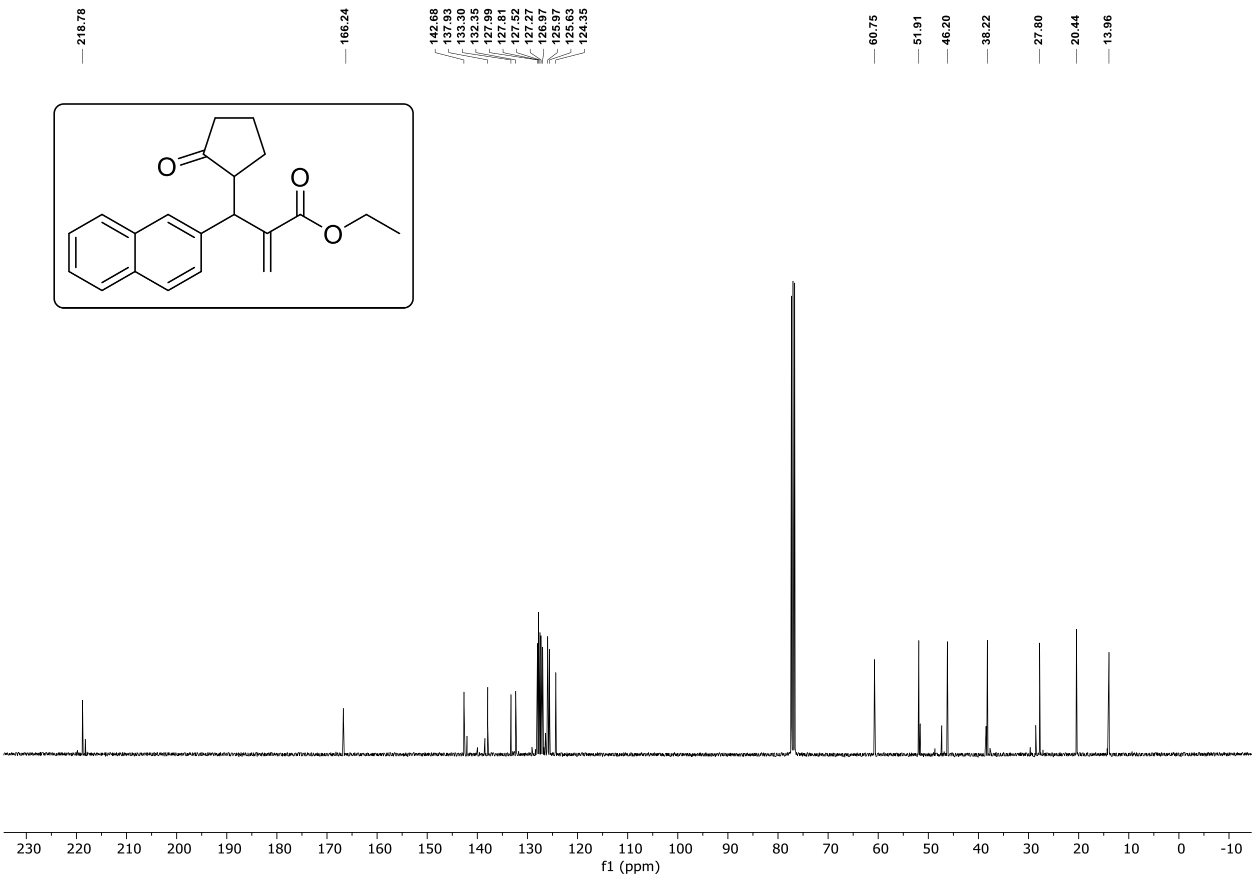

Figure S4. ^{1}H NMR spectrum of Ethyl 2-(naphthalen-2-yl(2-oxocyclohexyl)methyl)acrylate (**5**). (400 MHz, chloroform-d) (*dr* > 20:1)

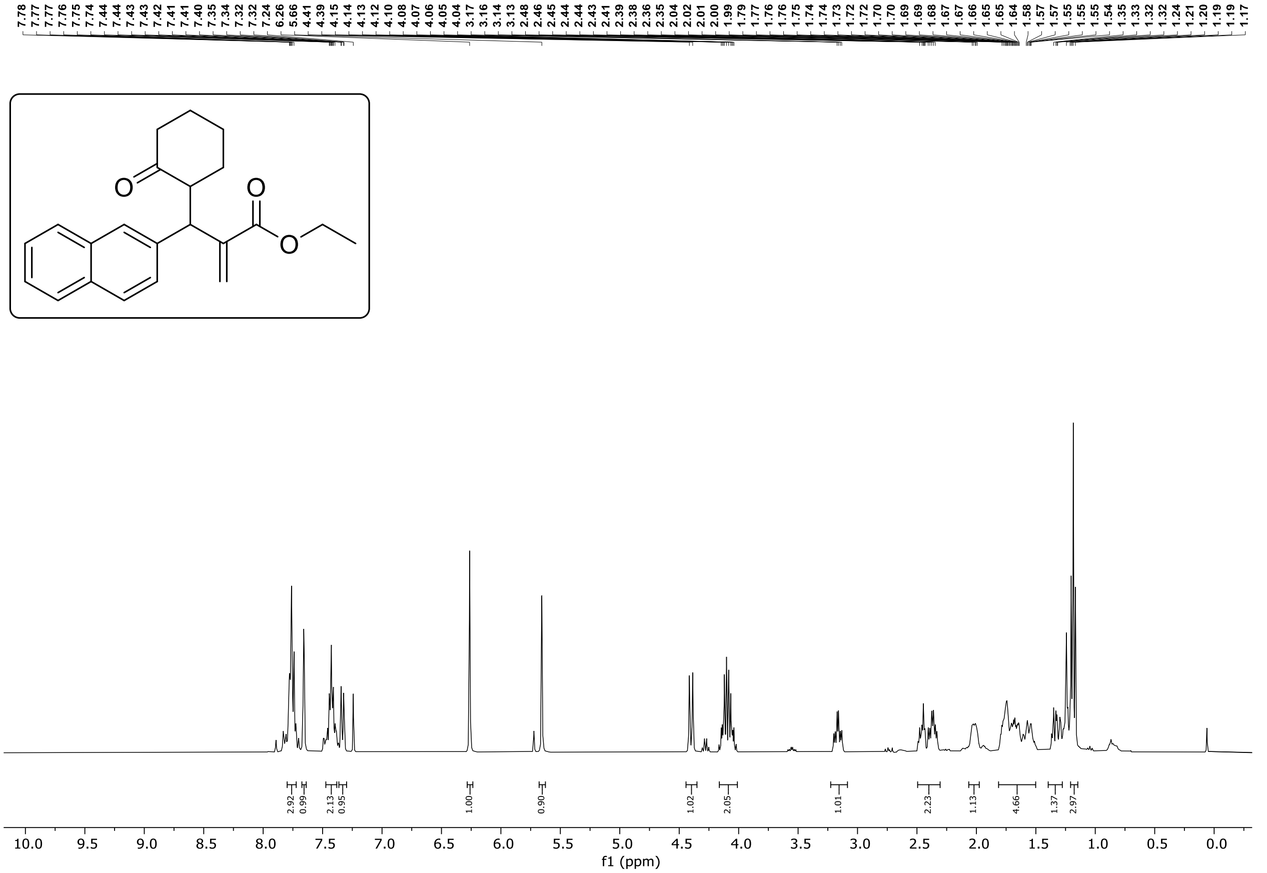

Figure S5. $^{13}C\{^{1}H\}$ NMR spectrum of Ethyl 2-(naphthalen-2-yl(2-oxocyclohexyl)methyl)acrylate (**5**). (100 MHz, chloroform-d) (*dr* > 20:1)

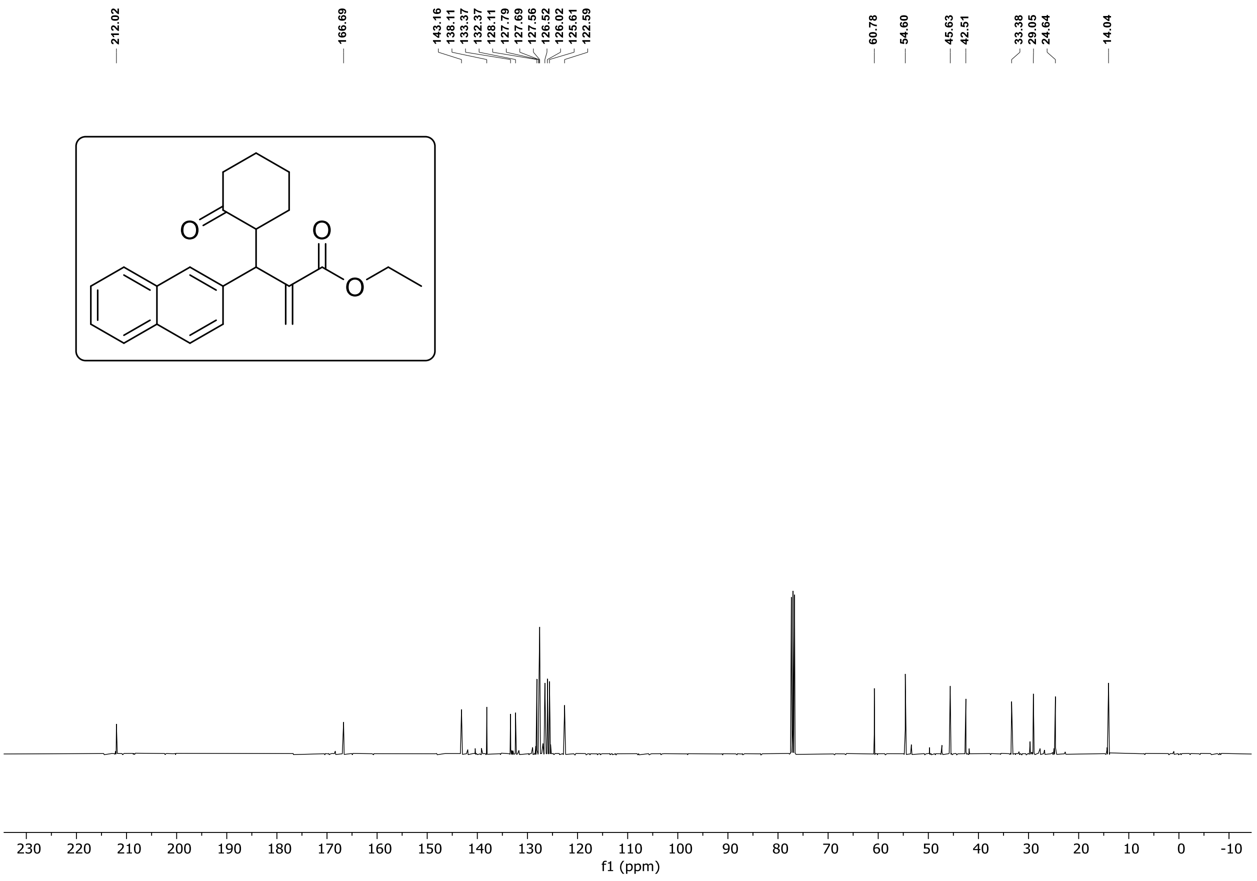

Figure S6. ^{1}H NMR spectrum of Ethyl (*E*)-3-(naphthalen-2-yl)-2-((2-oxocylcopentyl)methyl)acrylate (**8**). (400 MHz, chloroform-d) (*dr* = 16:1)

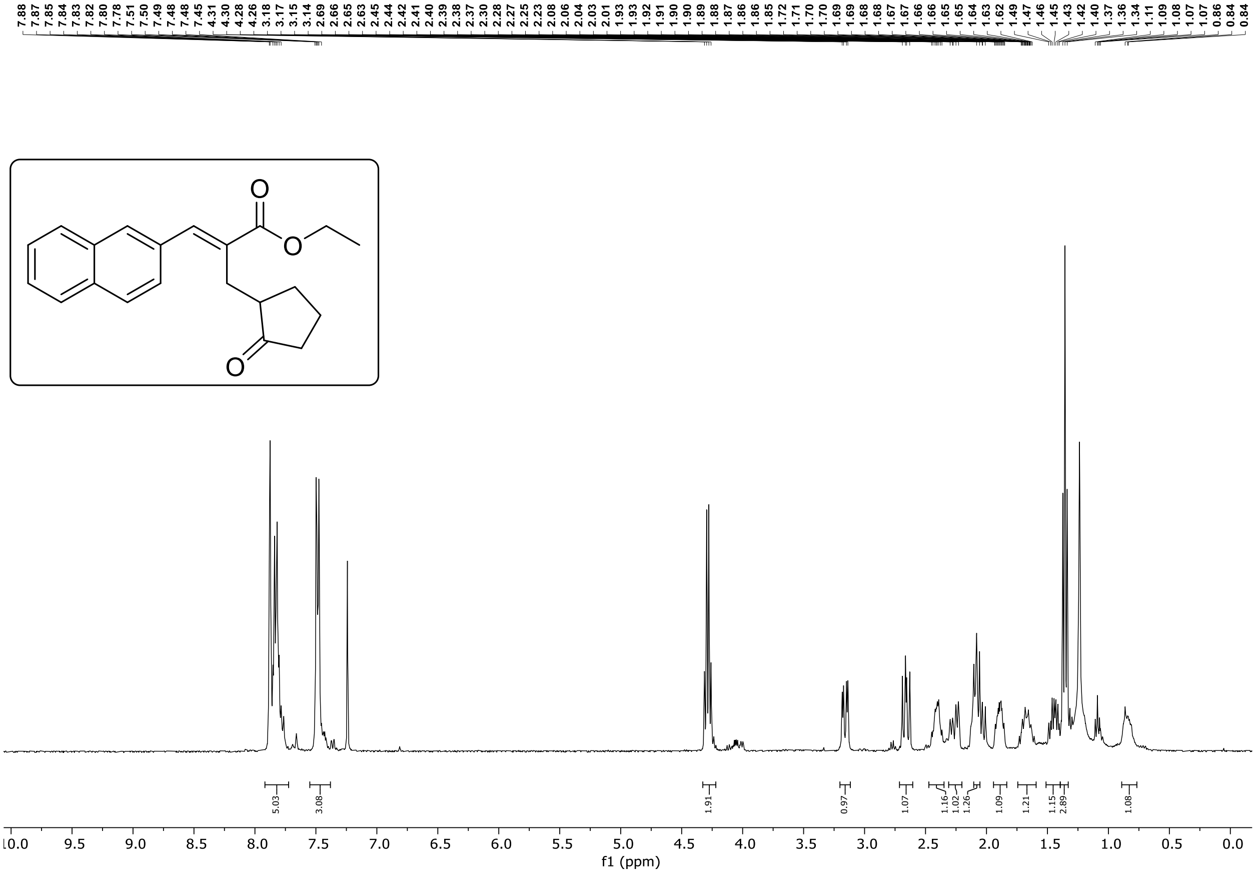

Figure S7. $^{13}C\{^{1}H\}$ NMR spectrum of Ethyl (*E*)-3-(naphthalen-2-yl)-2-((2-oxocylcopentyl)methyl)acrylate (**8**). (100 MHz, chloroform-d) (*dr* = 16:1)

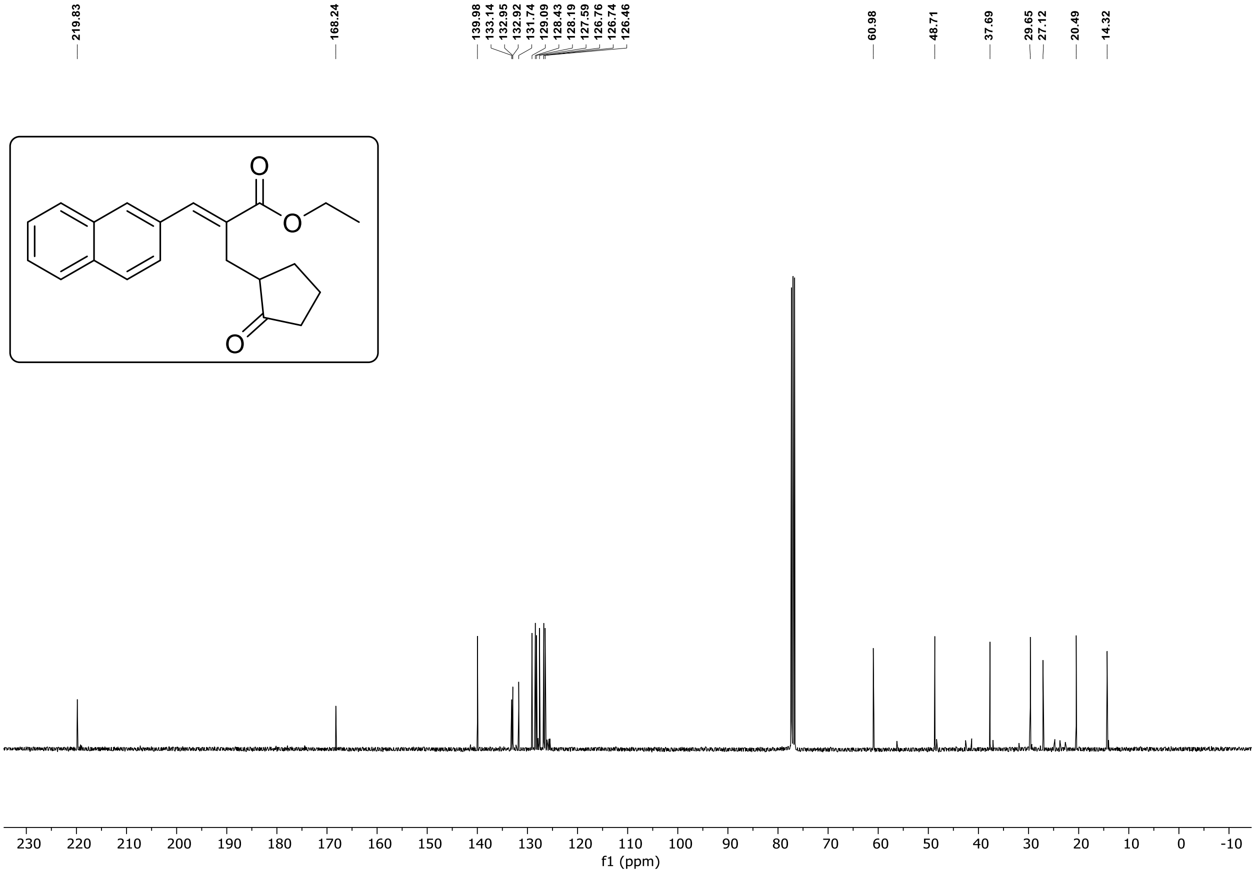

Figure S8. ^{1}H NMR spectrum of Ethyl (*E*)-3-(naphthalen-2-yl)-2-((2-oxocylcohexyl)methyl)acrylate (**9**). (400 MHz, chloroform-d) (*dr* > 20:1)

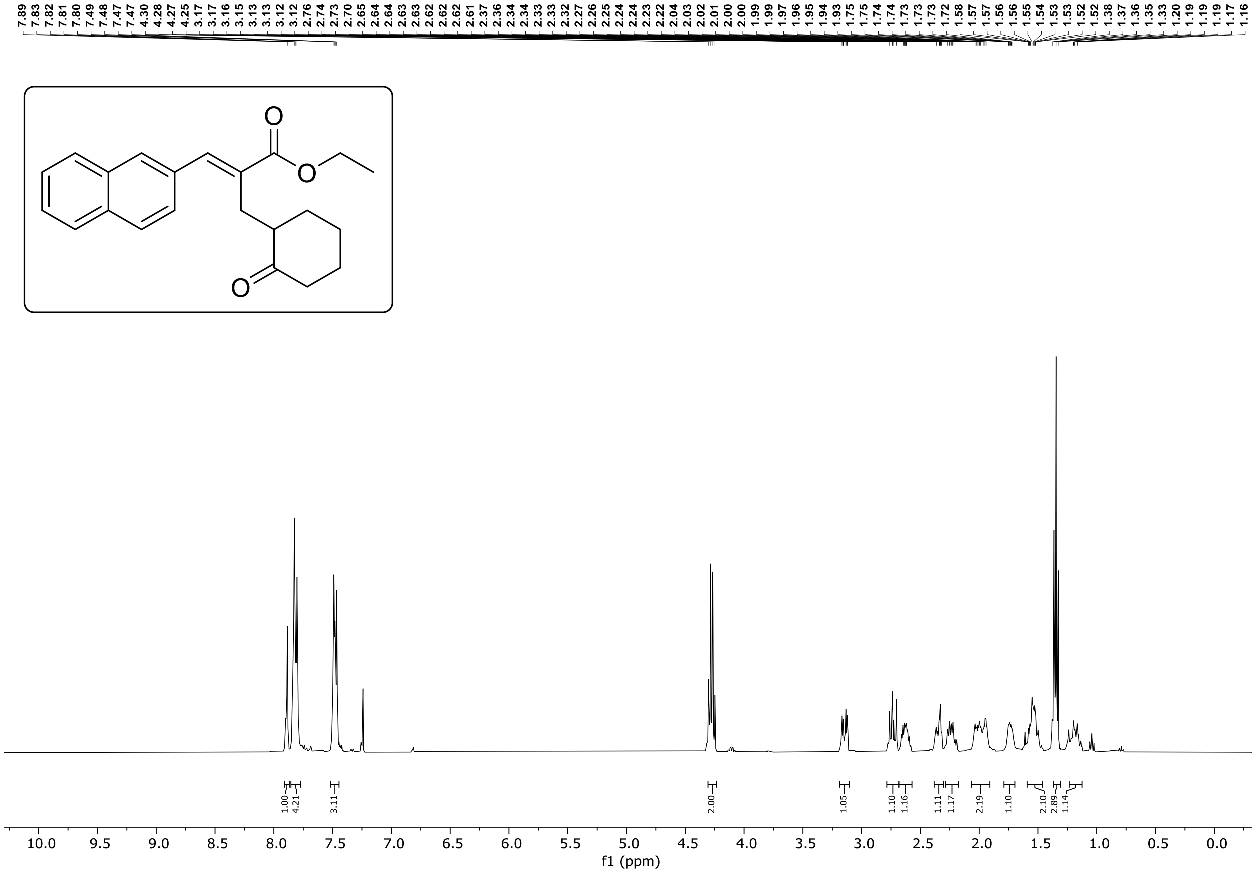

Figure S9. $^{13}C\{^1H\}$ NMR spectrum of Ethyl (*E*)-3-(naphthalen-2-yl)-2-((2-oxocylcohexyl)methyl)acrylate (**9**). (100 MHz, chloroform-d) (*dr* > 20:1)

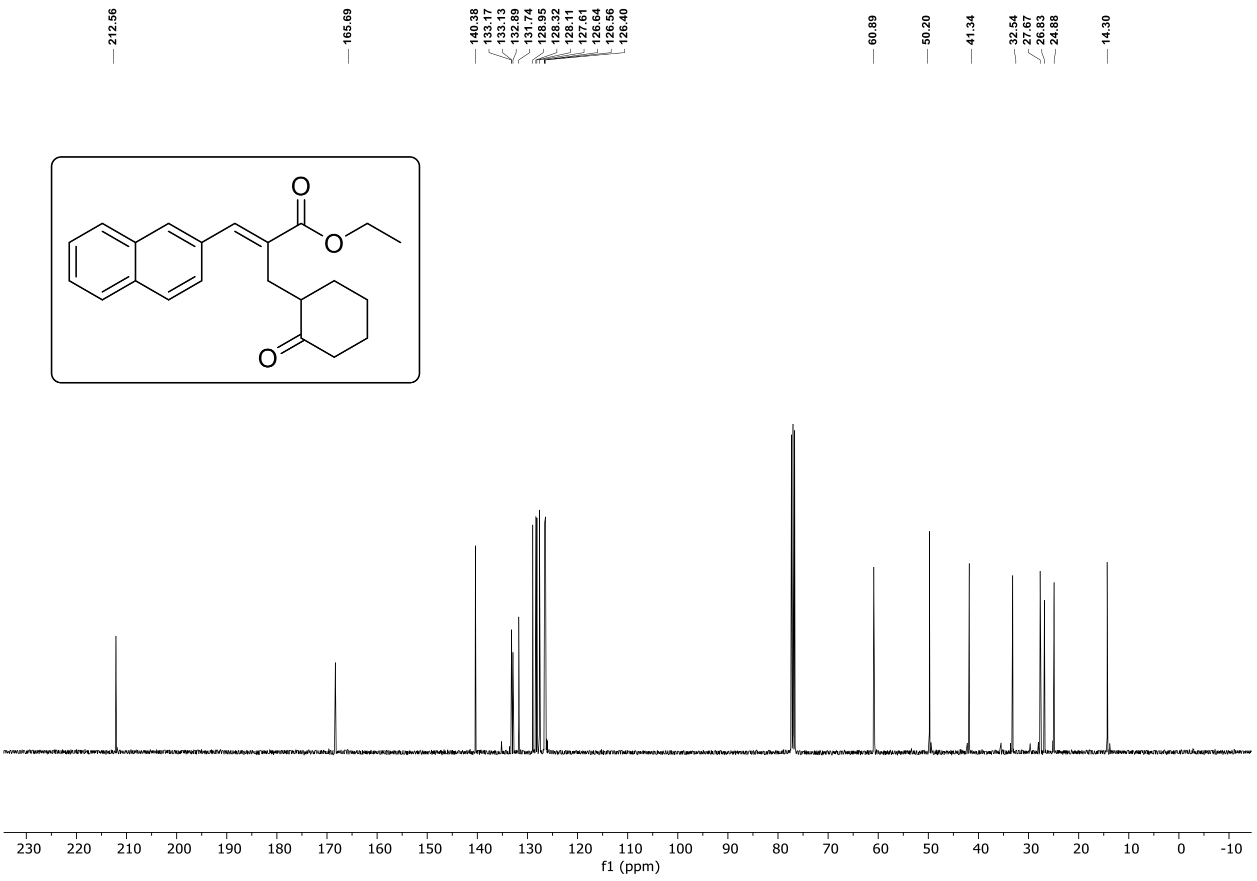

Figure S10. ^{1}H NMR spectrum of Ethyl (*Z*)-2-(iodomethyl)-3-phenylacrylate (**13**). (400 MHz, chloroform-d) (>20:1 *dr*)

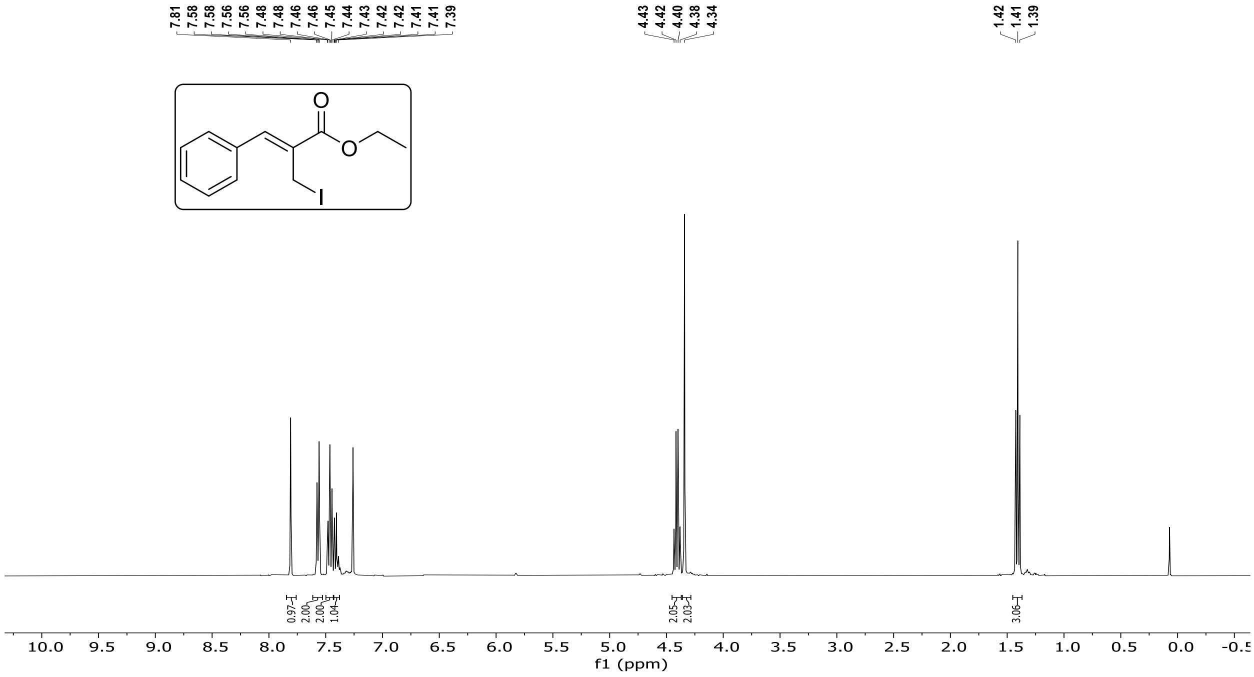

Figure S11. $^{13}C\{^1H\}$ NMR spectrum of Ethyl (*Z*)-2-(iodomethyl)-3-phenylacrylate (**13**). (100 MHz, chloroform-d) (>20:1 *dr*)

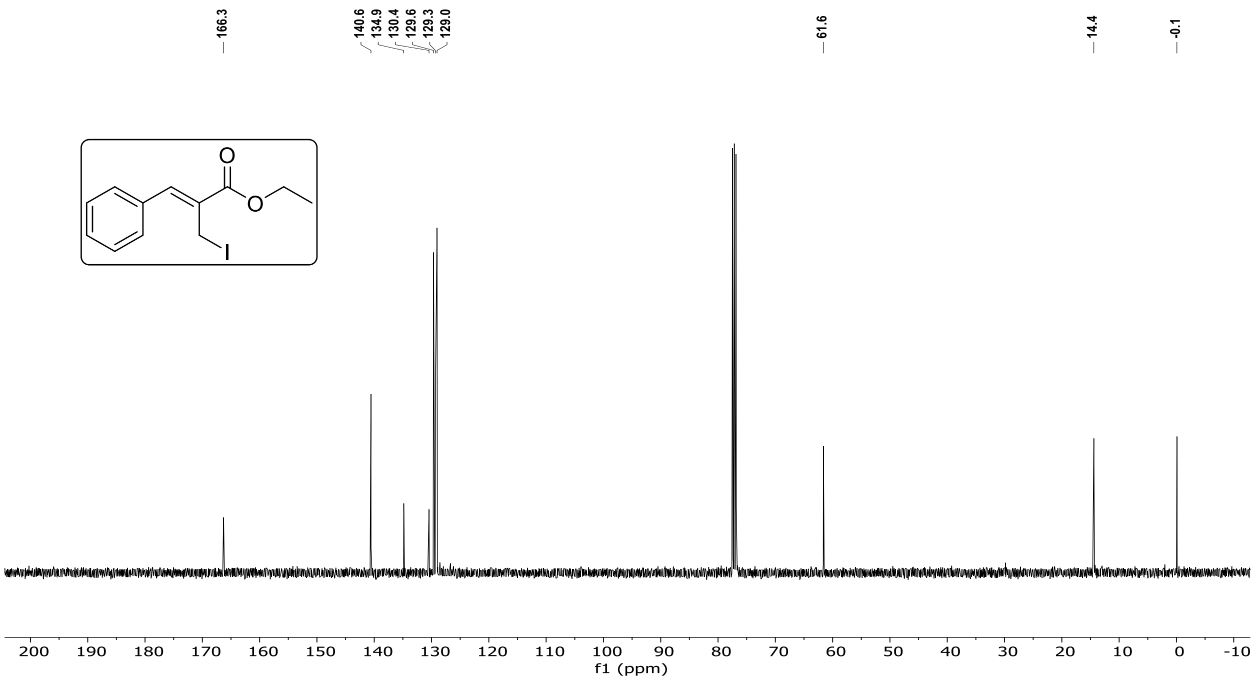

Figure S12. ^{1}H NMR spectrum of Ethyl (*E*)-2-(allylamino)methyl)-3-phenylacrylate (**14**). (400 MHz, methanol-d_4) (>20:1 *dr*)

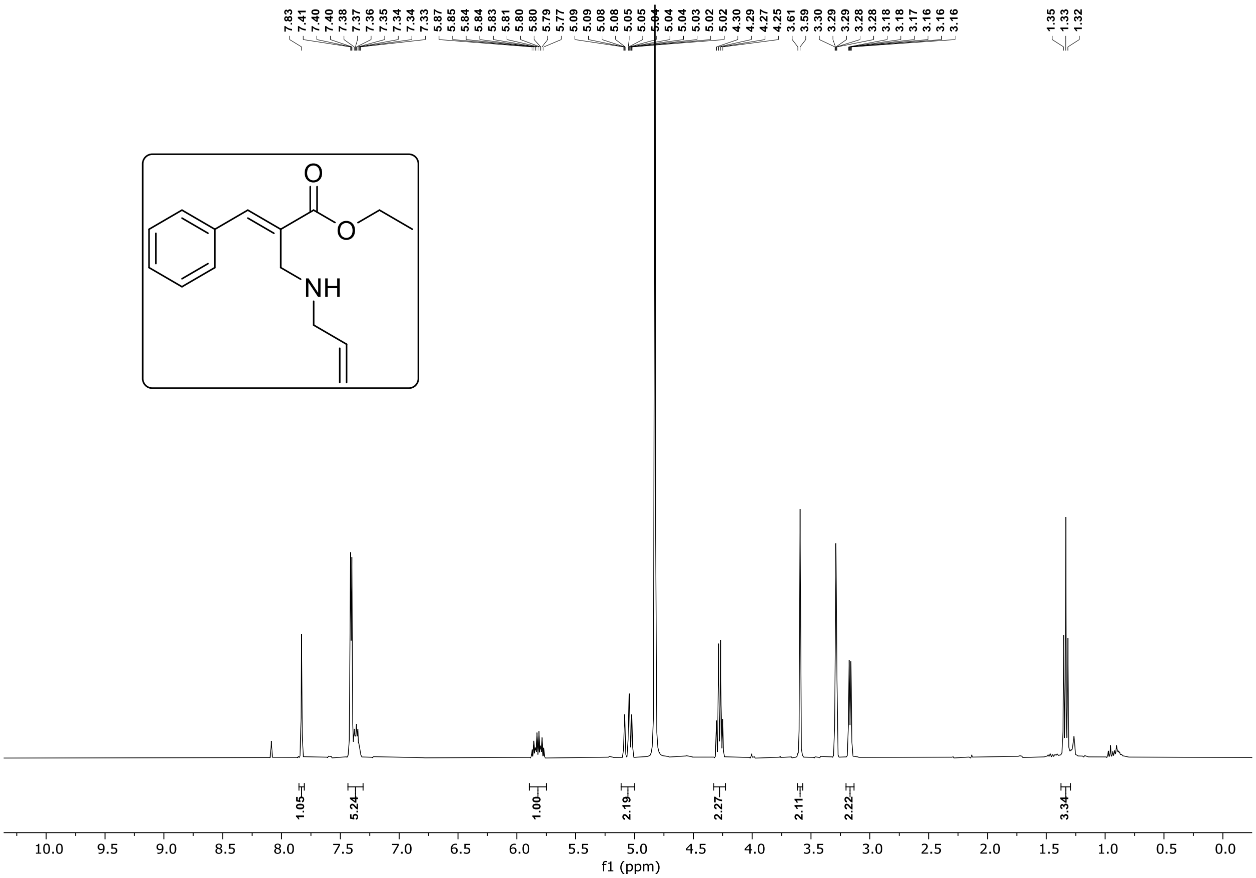

Figure S13. $^{13}C\{^1H\}$ NMR spectrum of Ethyl (*E*)-2-(allylamino)methyl)-3-phenylacrylate (**14**). (100 MHz, methanol-d$_4$) (>20:1 *dr*)

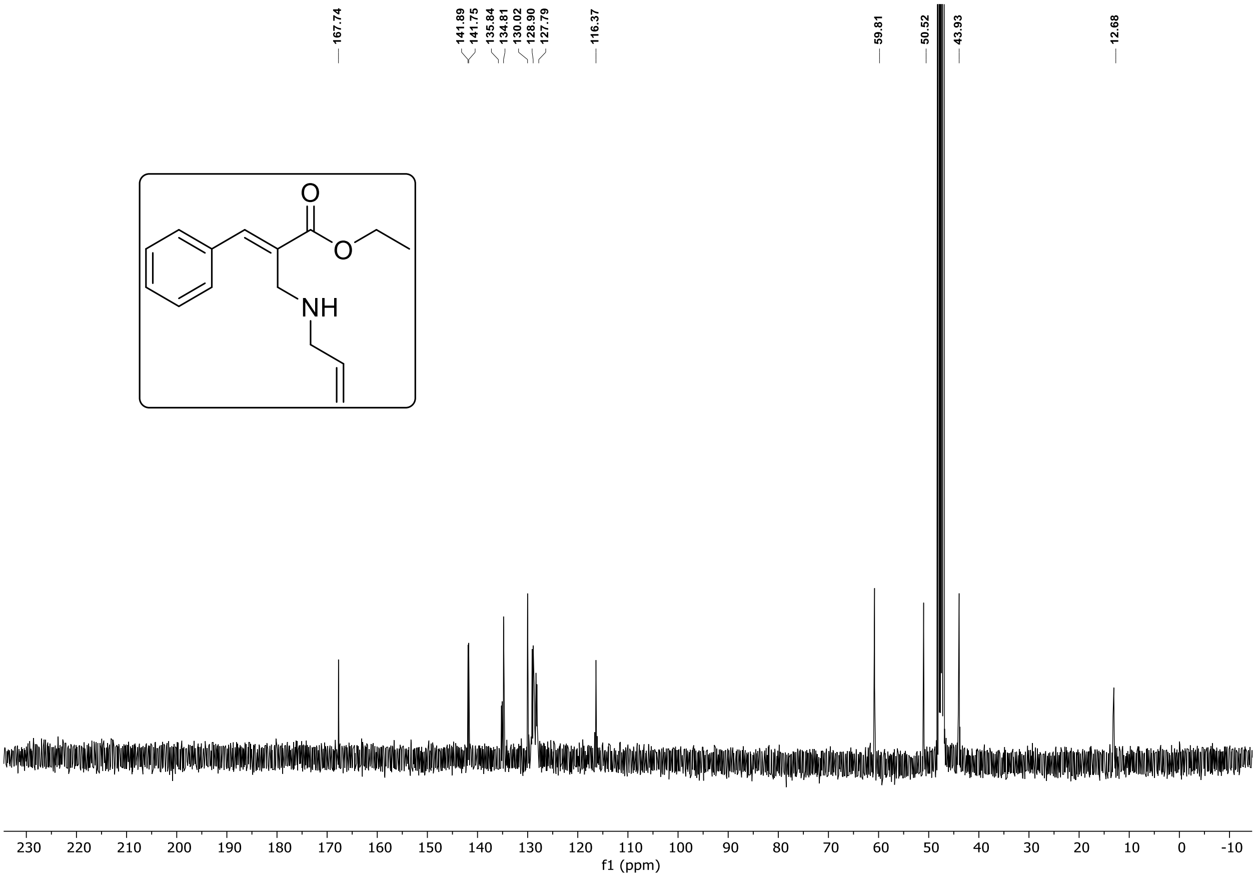

Figure S14. ^{1}H NMR spectrum of Methyl (*E*)-2-(allylamino)methyl)-3-phenylacrylate (**15**). (400 MHz, chloroform-d) (>20:1 *dr*)

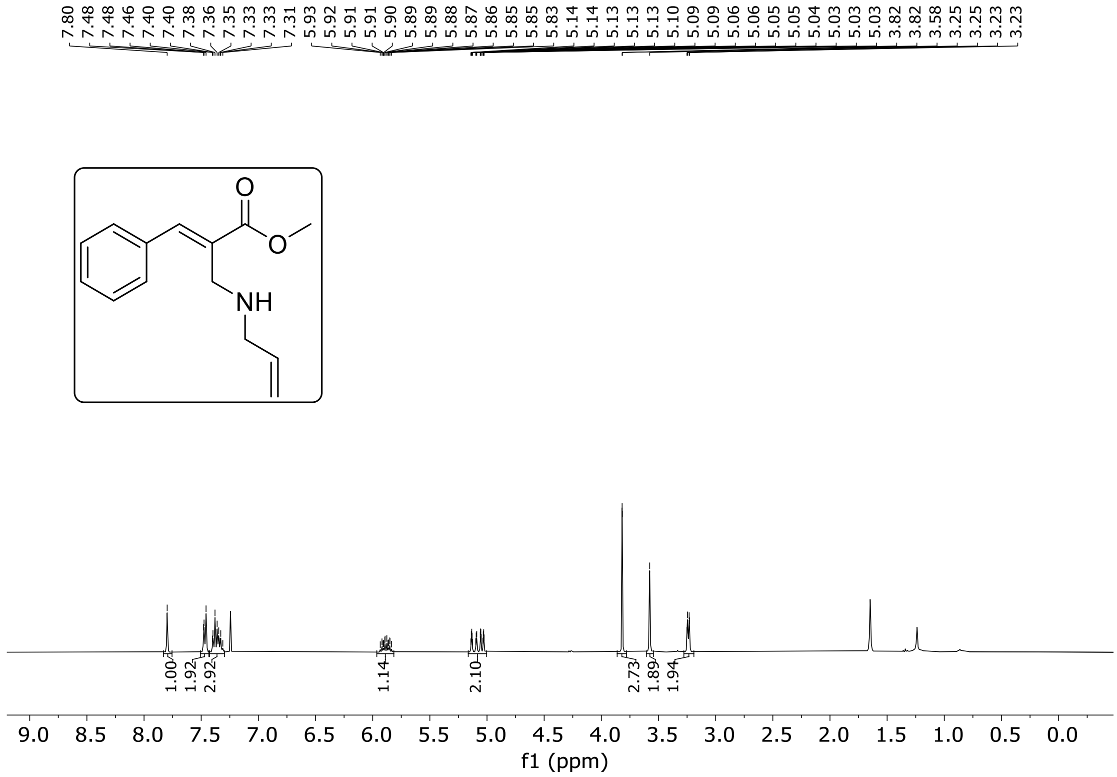

Figure S15. $^{13}C\{^{1}H\}$ NMR spectrum of Methyl (*E*)-2-(allylamino)methyl)-3-phenylacrylate (**15**). (100 MHz, chloroform-d) (>20:1 *dr*)

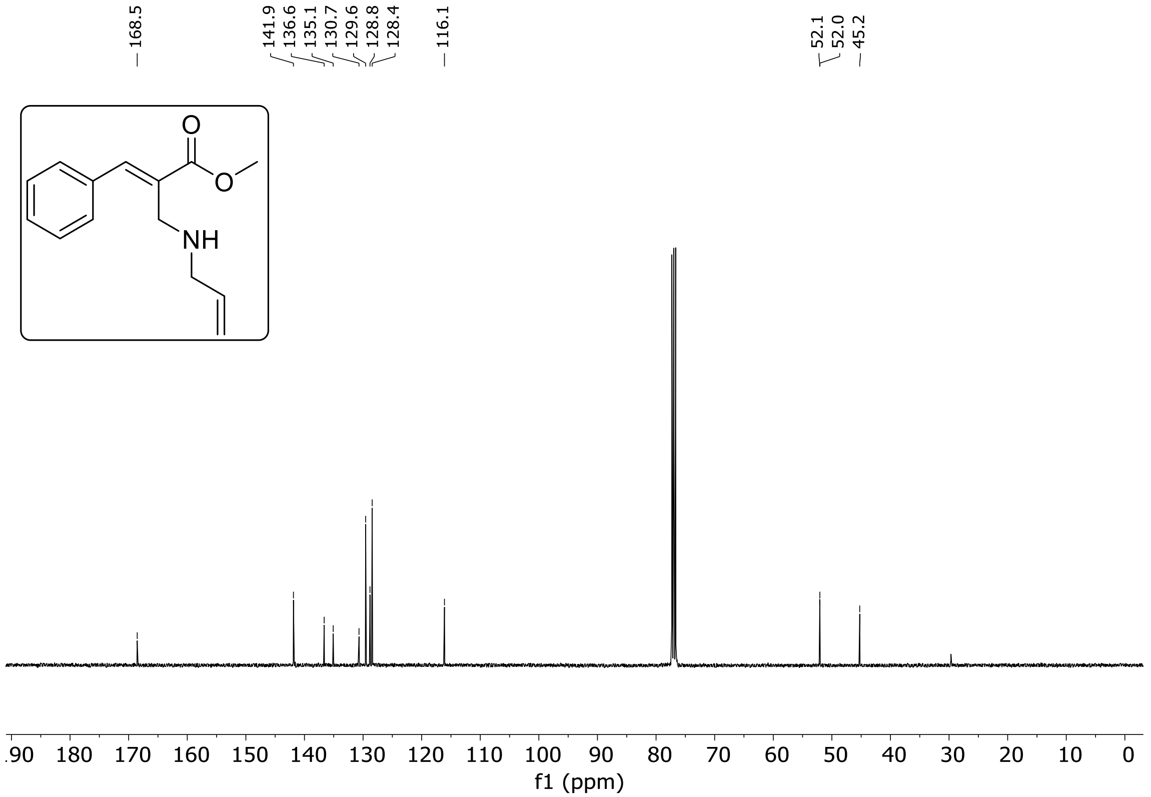

Figure S16. ^{1}H NMR spectrum of *tert*-Butyl (*E*)-2-(allylamino)methyl)-3-phenylacrylate (**16**). (400 MHz, chloroform-d) (>20:1 *dr*)

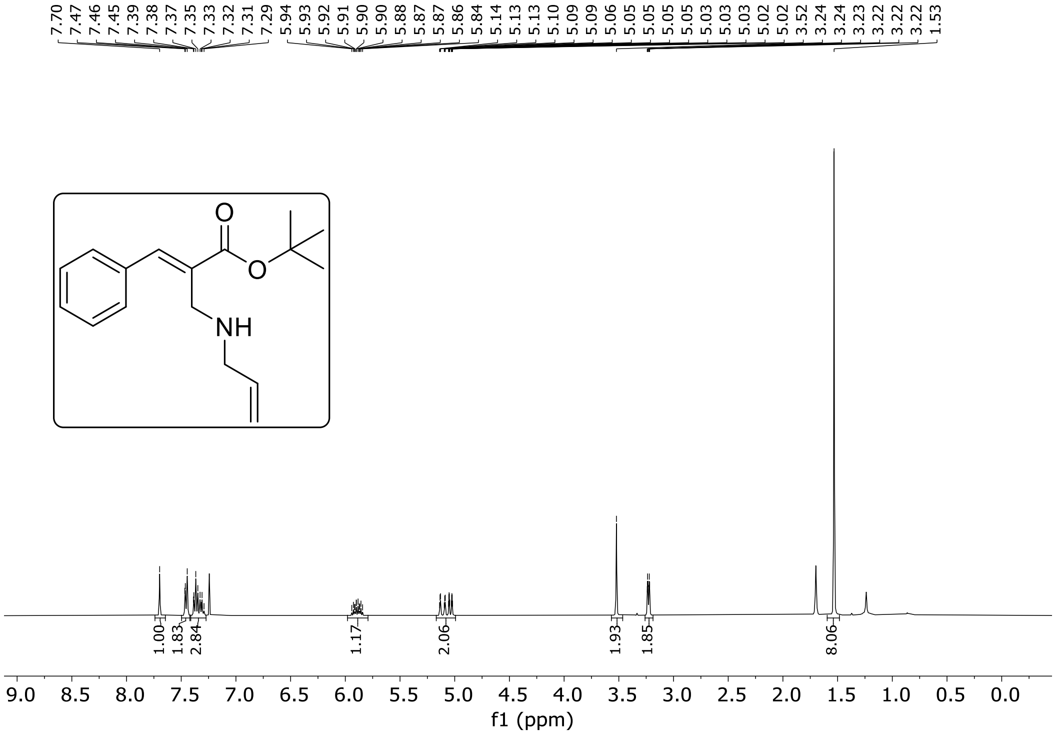

Figure S17. ^{13}C{^{1}H} NMR spectrum of *tert*-Butyl (*E*)-2-(allylamino)methyl)-3-phenylacrylate (**16**). (100 MHz, chloroform-d) (>20:1 *dr*)

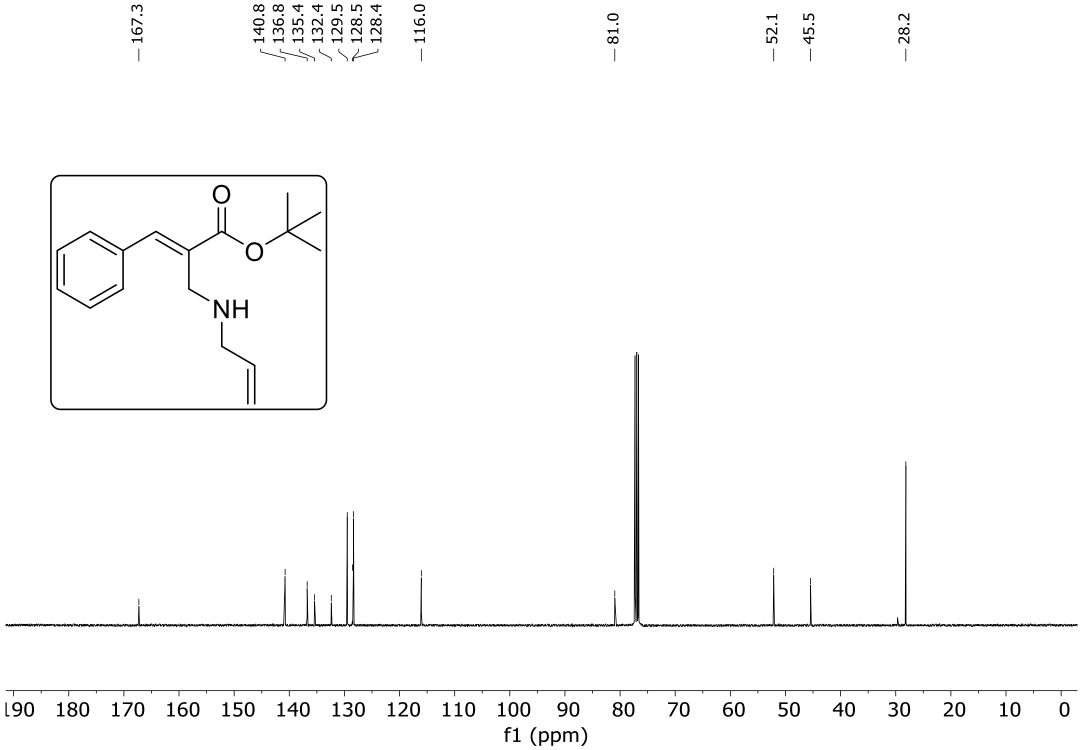

Figure S18. ^{1}H NMR spectrum of Benzyl (*E*)-2-(allylamino)methyl)-3-phenylacrylate (**17**). (400 MHz, chloroform-d) (>20:1 *dr*)

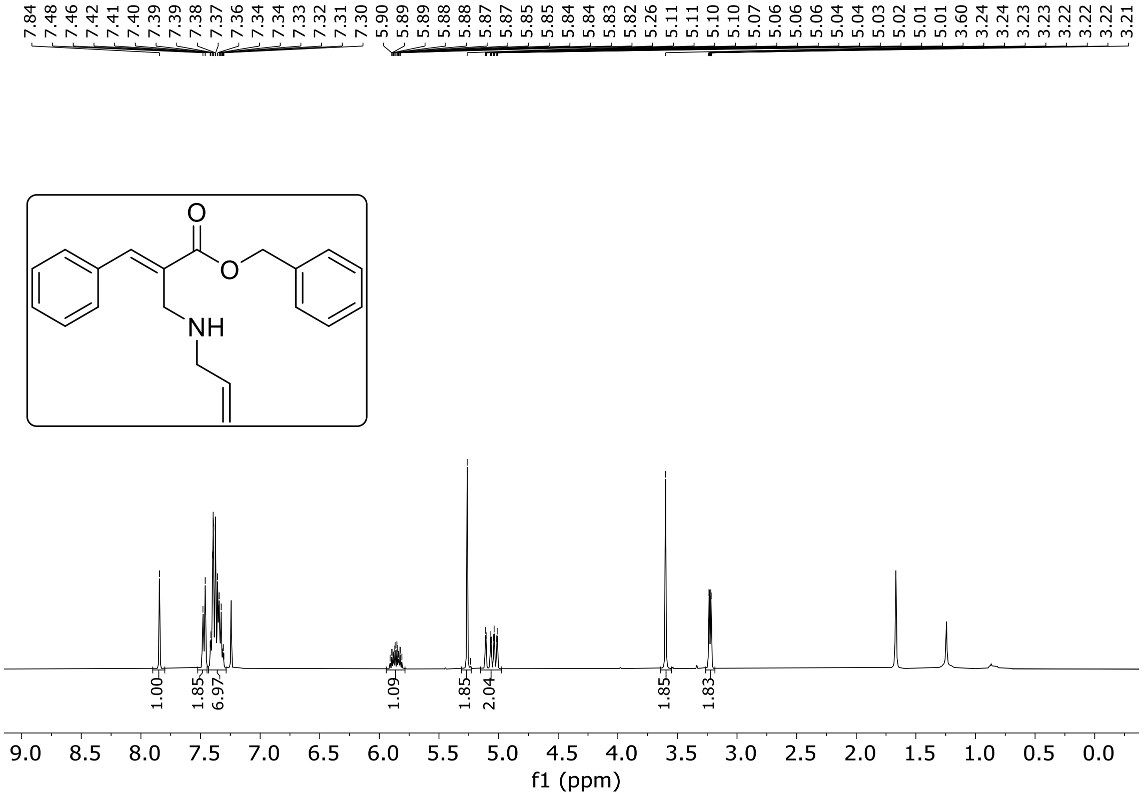

Figure S19. $^{13}C\{^{1}H\}$ NMR spectrum of Benzyl (*E*)-2-(allylamino)methyl)-3-phenylacrylate (**17**). (100 MHz, chloroform-d) (>20:1 *dr*)

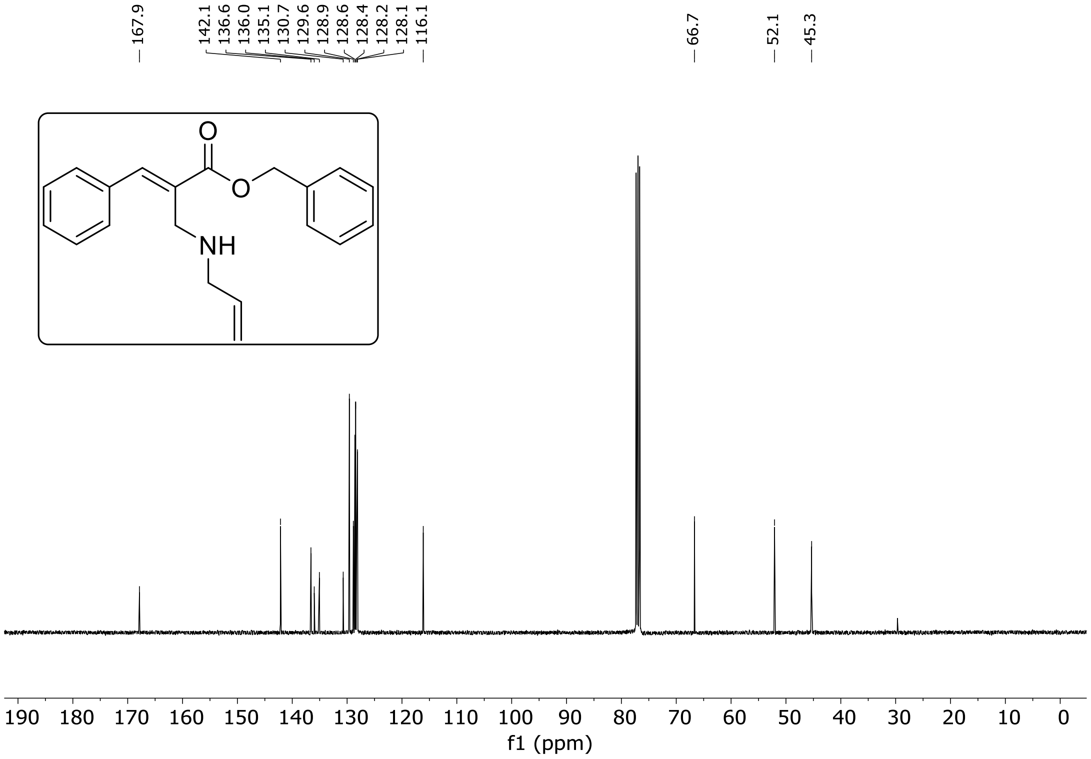

Figure S20. ^{1}H NMR spectrum of Ethyl (*E*)-2-((*tert*-butylamino)methyl)-3-phenylacrylate (**18**). (400 MHz, chloroform-d) (>20:1 *dr*)

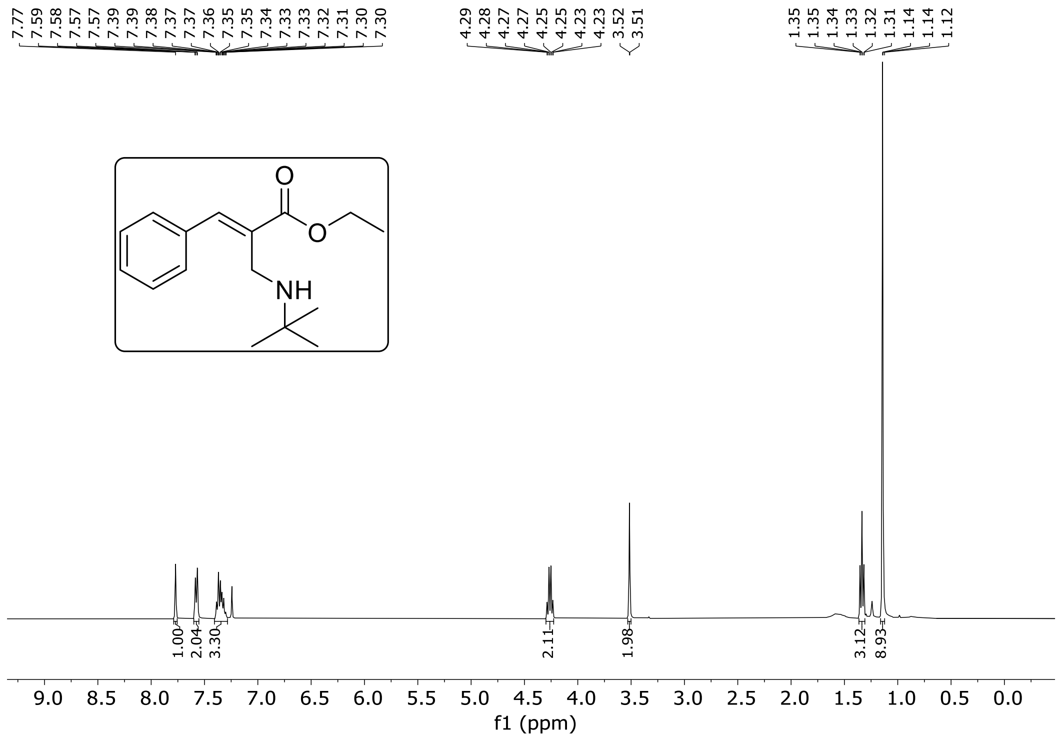

Figure S21. $^{13}C\{^1H\}$ NMR spectrum of Ethyl (*E*)-2-((*tert*-butylamino)methyl)-3-phenylacrylate (**18**). (100 MHz, chloroform-d) (>20:1 *dr*)

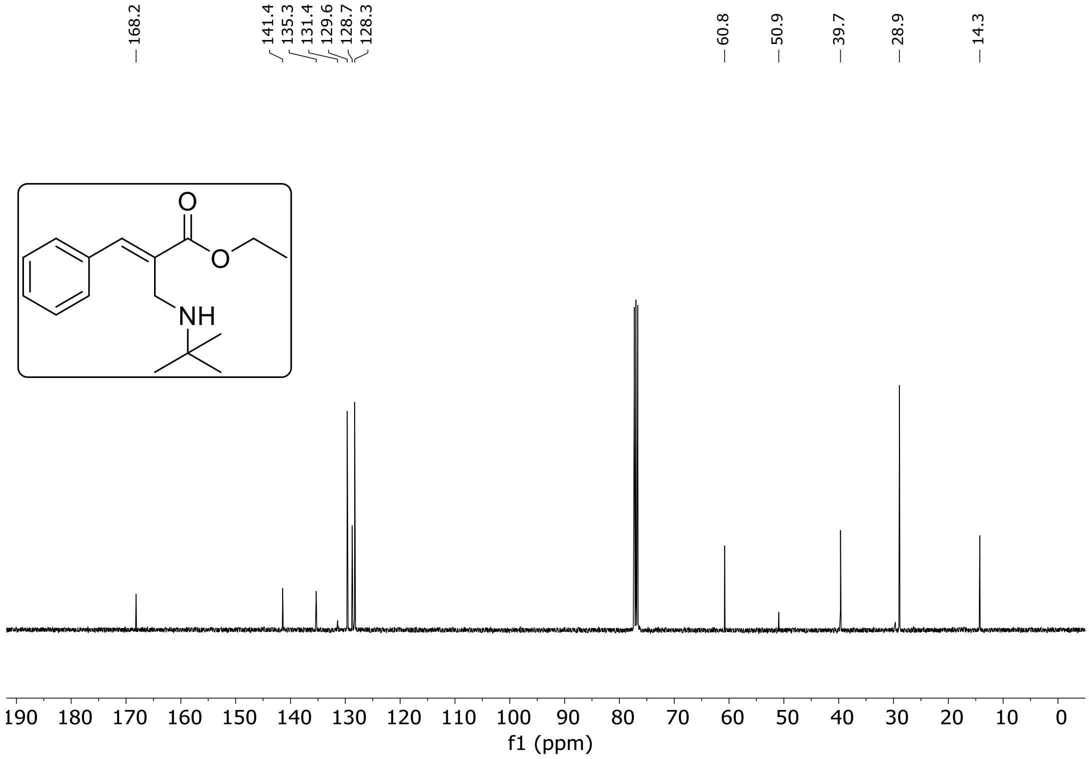

Figure S22. ^1H NMR spectrum of Ethyl (*E*)-3-phenyl-2-((phenylamino)methyl)acrylate (**19**). (400 MHz, chloroform-d) (>20:1 *dr*)

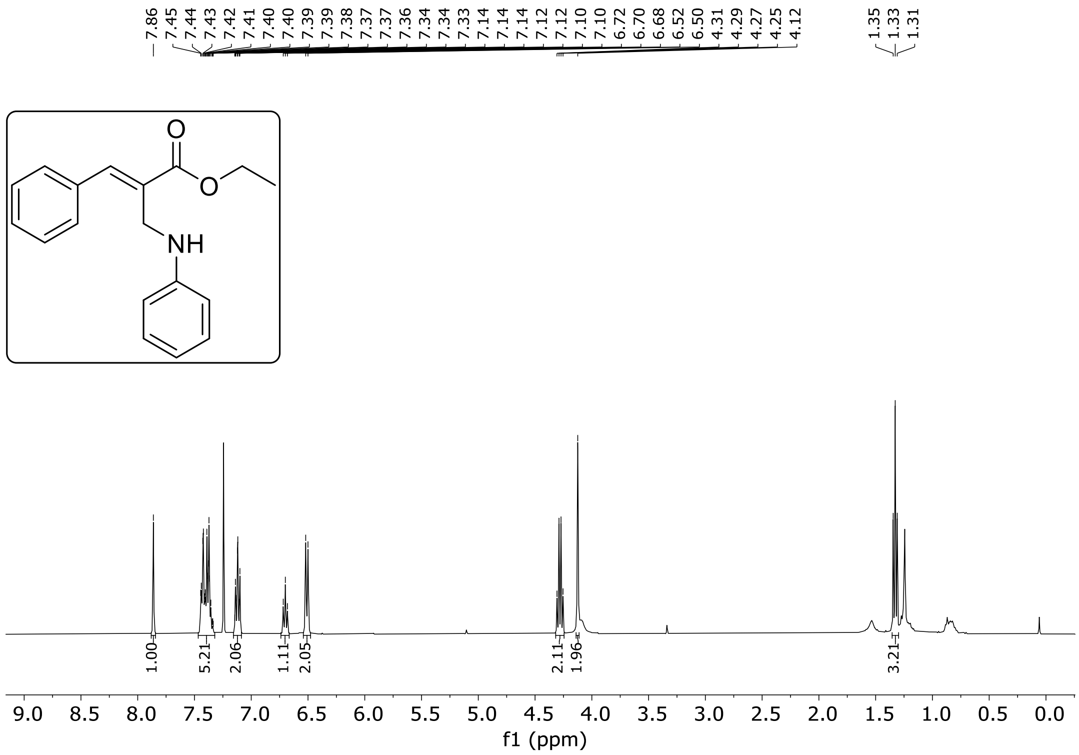

Figure S23. $^{13}C\{^{1}H\}$ NMR spectrum of Ethyl (*E*)-3-phenyl-2-((phenylamino)methyl)acrylate (**19**). (100 MHz, chloroform-d) (>20:1 *dr*)

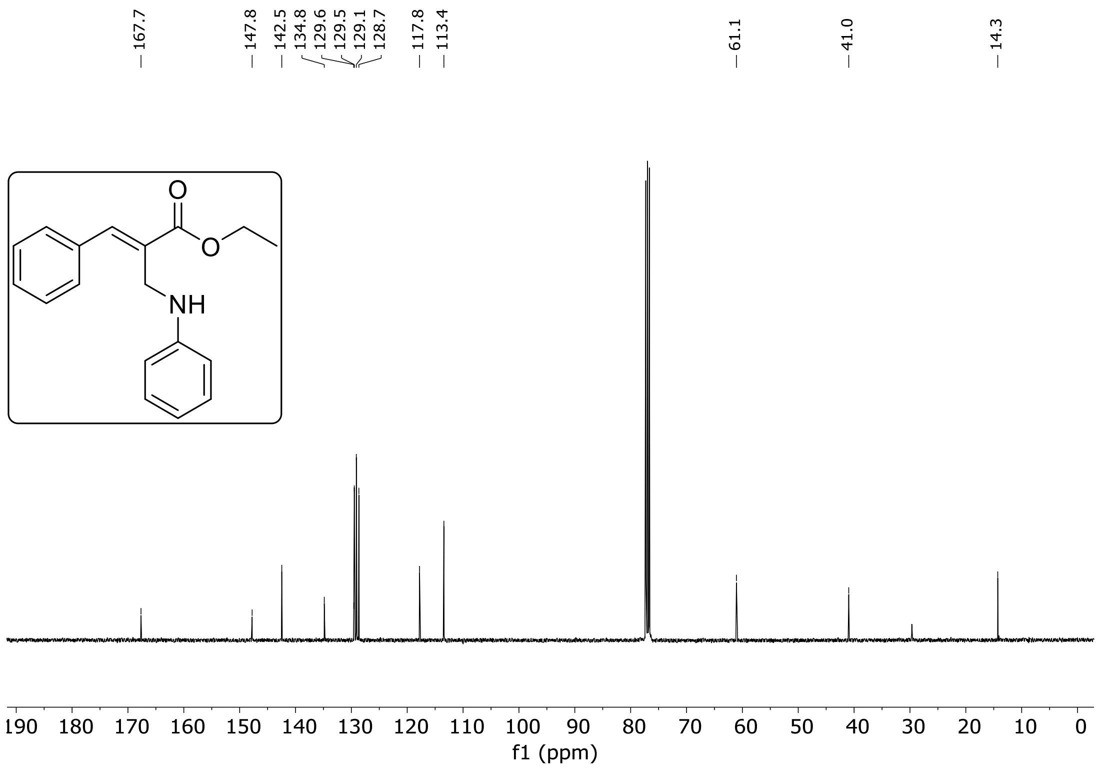

Figure S24. ^{1}H NMR spectrum of Ethyl (*E*)-2-((benzylamino)methyl)-3-phenylacrylate (**20**). (400 MHz, chloroform-d) (>20:1 *dr*)

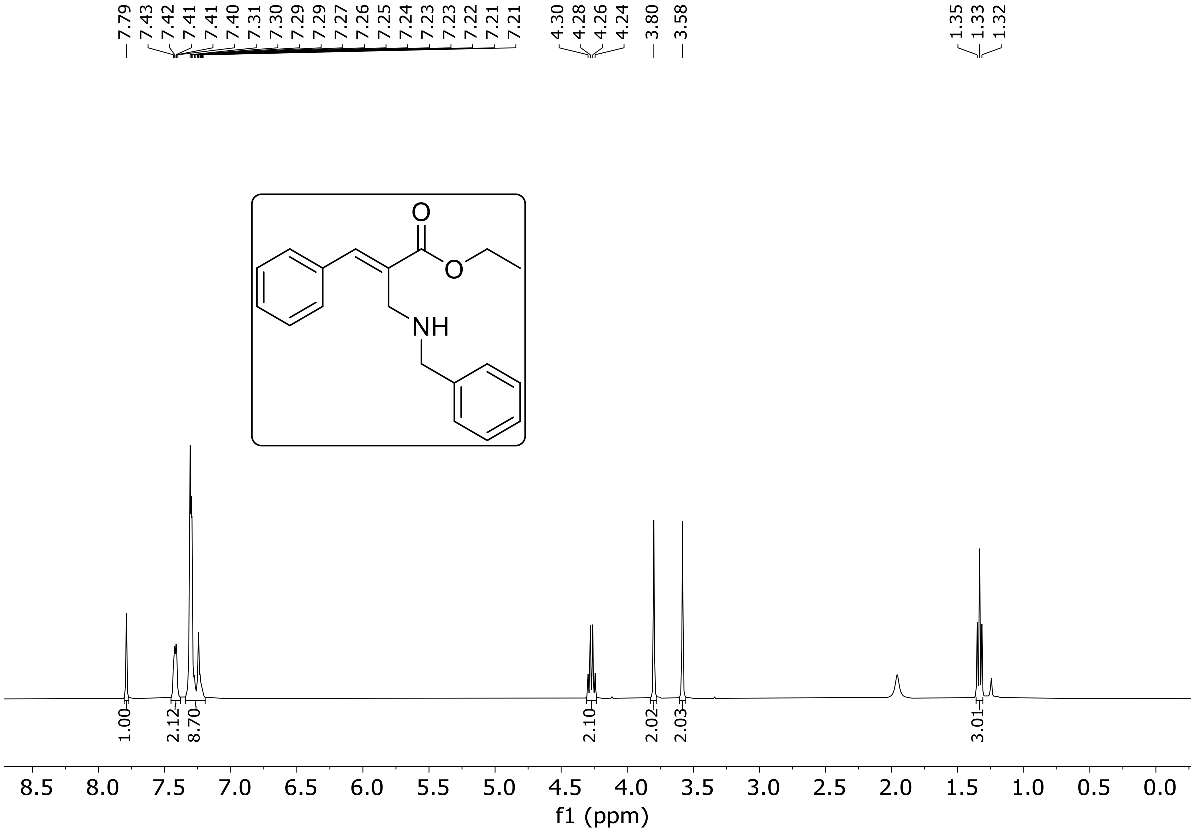

Figure S25. $^{13}C\{^{1}H\}$ NMR spectrum of Ethyl (*E*)-2-((benzylamino)methyl)-3-phenylacrylate (**20**). (100 MHz, chloroform-d) (>20:1 *dr*)

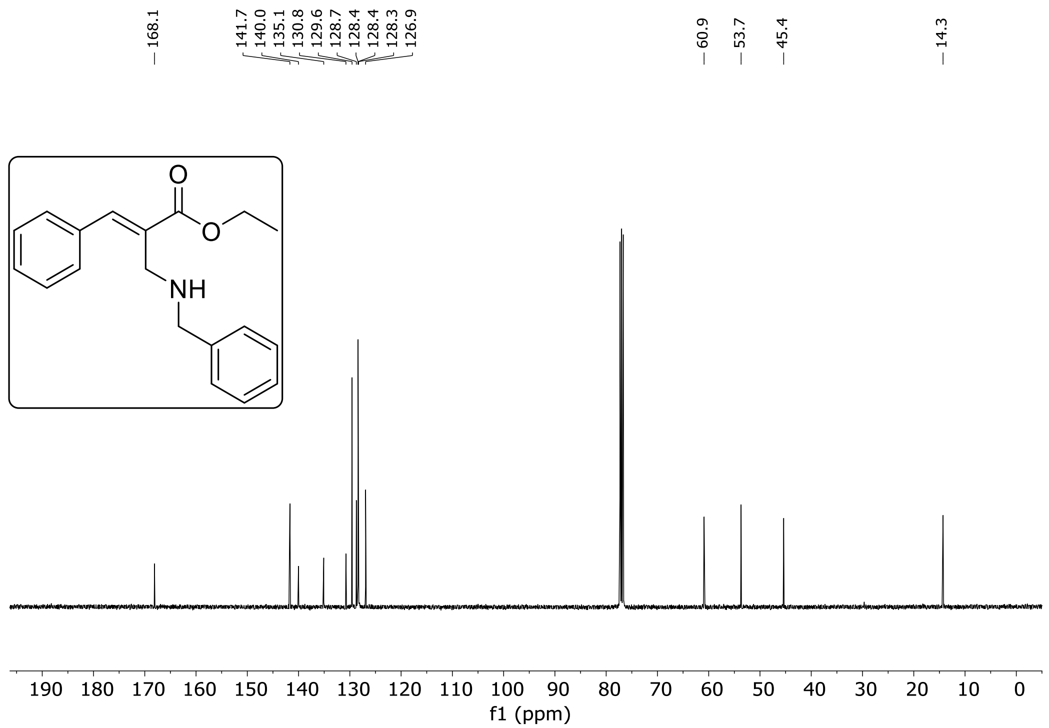

Figure S26. ^{1}H NMR spectrum of Ethyl (*E*)-3-phenyl-2-(((2,2,2-trifluoromethyl)amino)methyl) acrylate (**21**). (400 MHz, chloroform-d) (>20:1 *dr*)

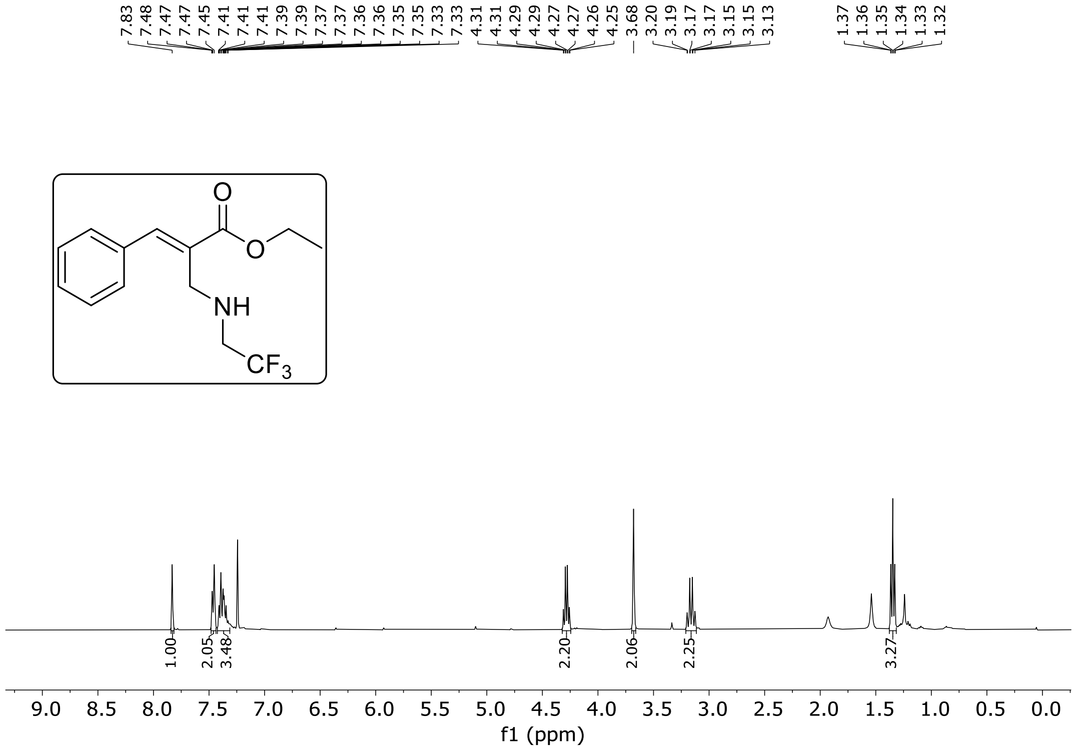

Figure S27. $^{13}C\{^{1}H\}$ NMR spectrum of Ethyl (*E*)-3-phenyl-2-(((2,2,2-trifluoromethyl)amino)methyl) acrylate (**21**). (100 MHz, chloroform-d) (>20:1 *dr*)

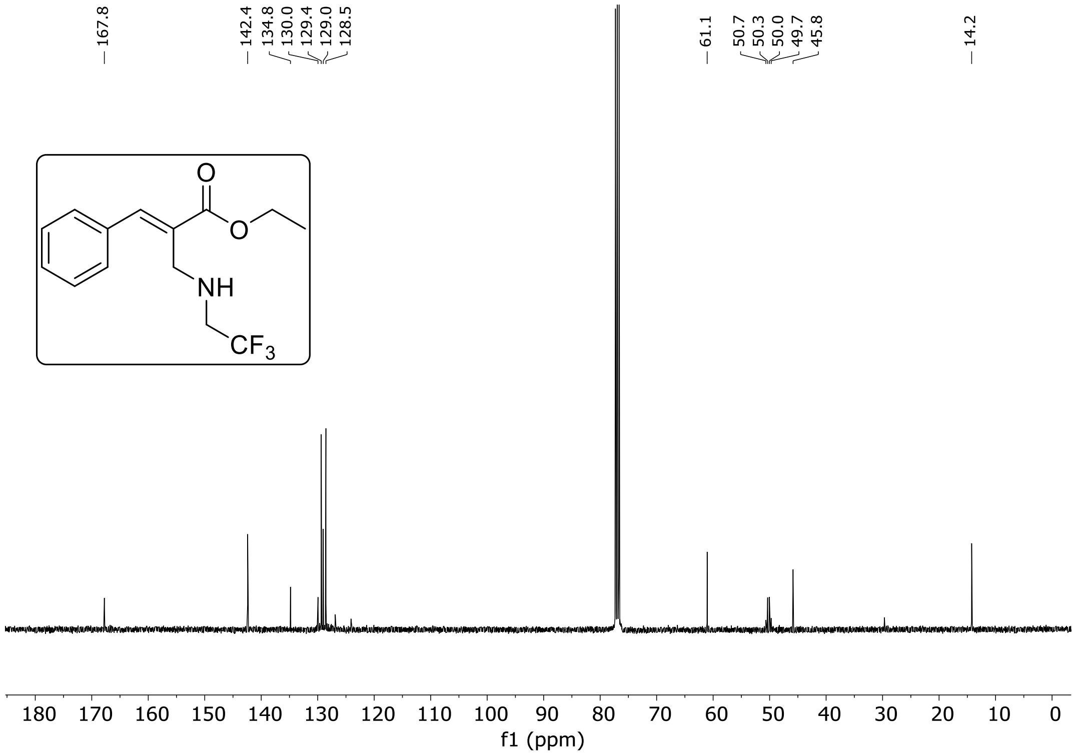

Figure S28. ^{19}F NMR spectrum of Ethyl (*E*)-3-phenyl-2-(((2,2,2-trifluoromethyl)amino)methyl) acrylate (**21**). (376 MHz, chloroform-d) (>20:1 *dr*)

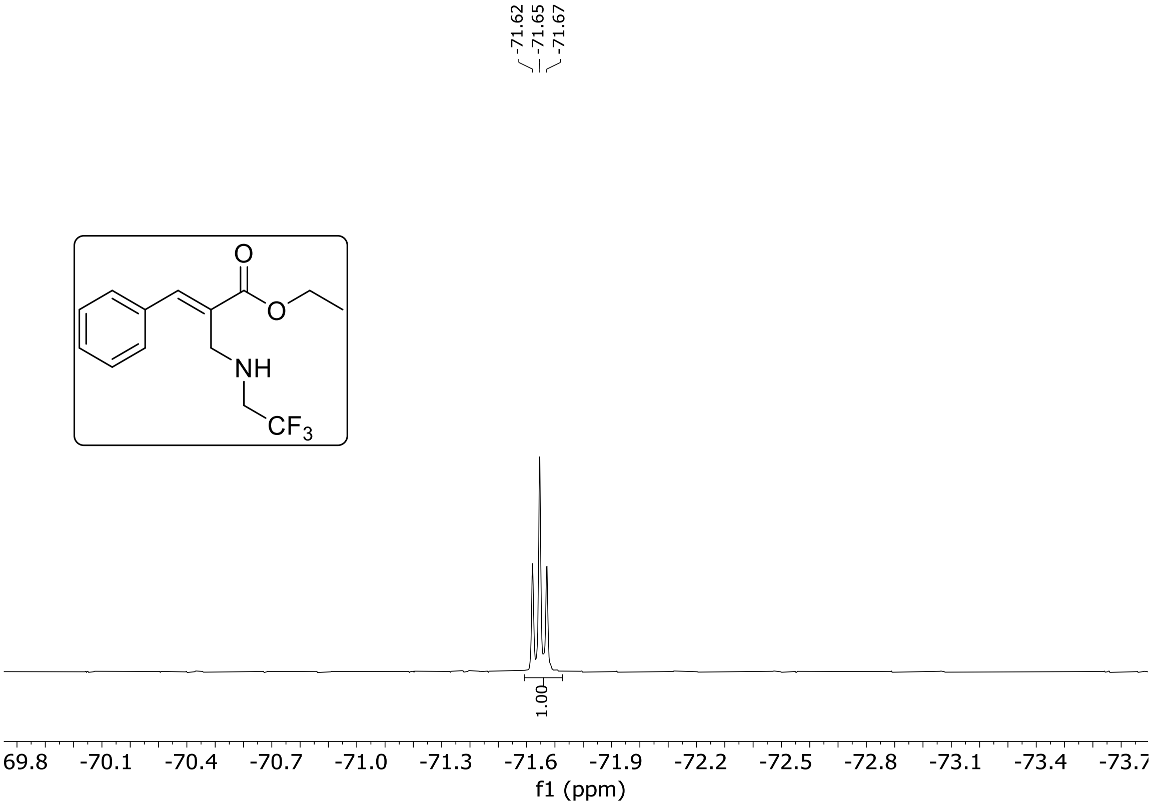

Figure S29. ^{1}H NMR spectrum of *tert*-Butyl (*E*)-2-((phenethylamino)methyl)-3-phenylacrylate (**22**). (400 MHz, chloroform-d) (>20:1 *dr*)

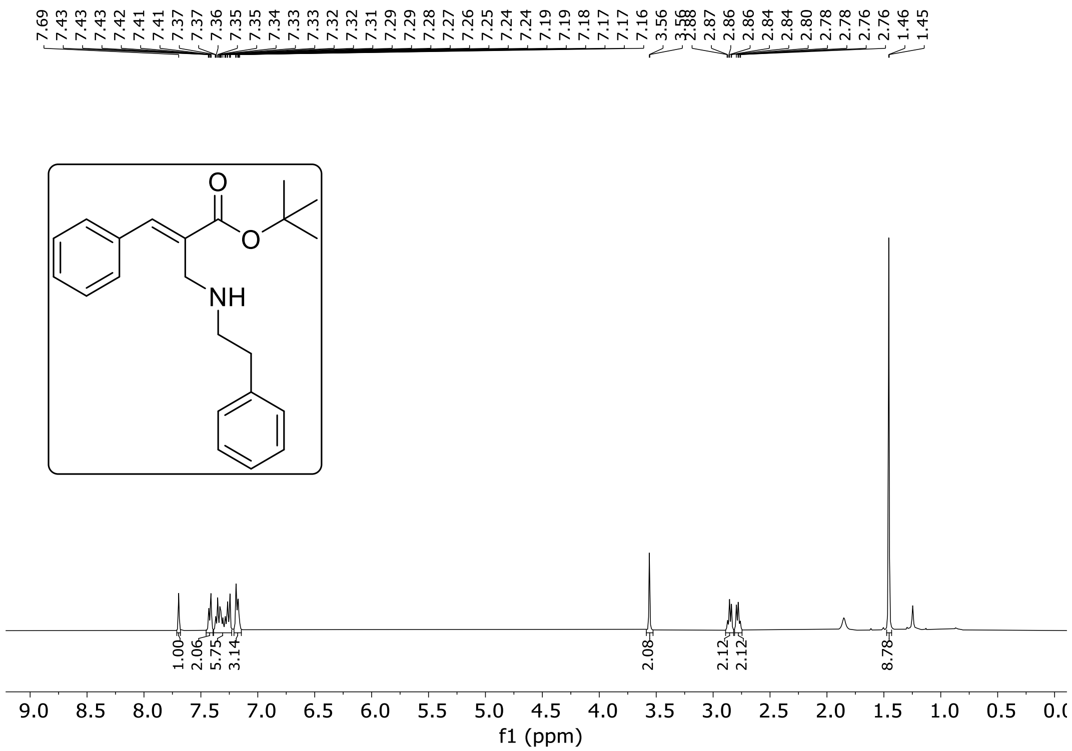

Figure S30. ^{13}C{^{1}H} NMR spectrum of *tert*-Butyl (*E*)-2-((phenethylamino)methyl)-3-phenylacrylate (**22**). (100 MHz, chloroform-d) (>20:1 *dr*)

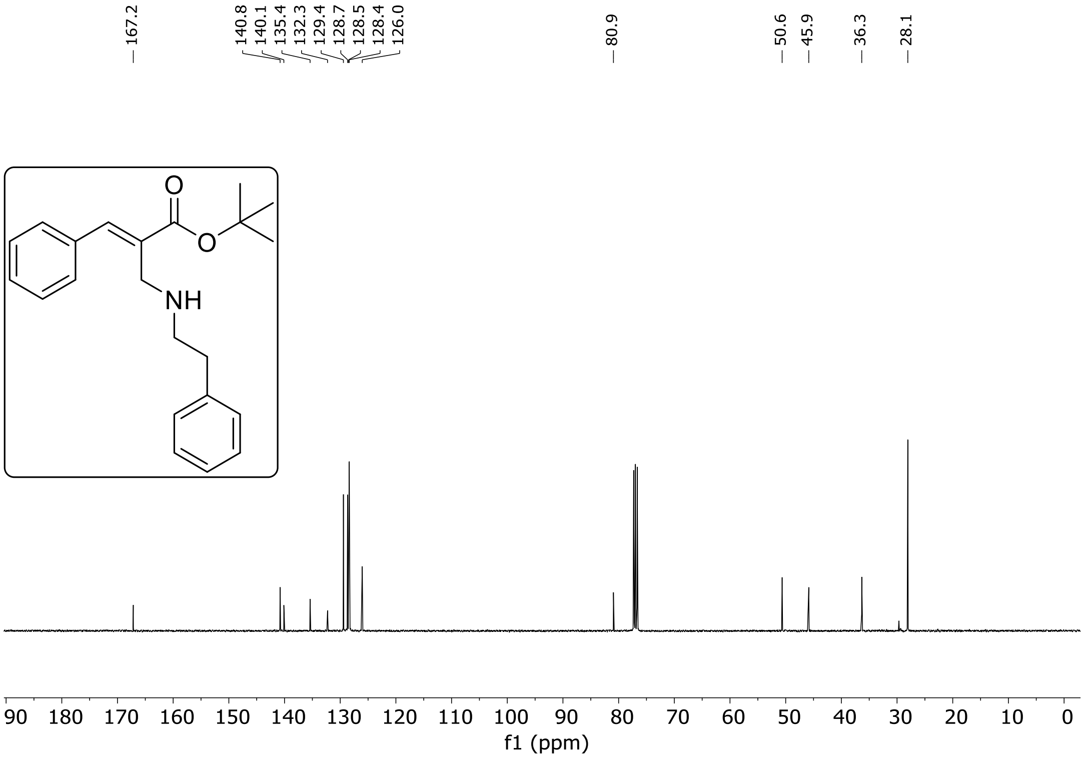

Figure S31. ^{1}H NMR spectrum of Ethyl (*E*)-2-((benzyl(methyl)amino)methyl)-3-phenylacrylate (**23**). (400 MHz, chloroform-d) (>20:1 *dr*)

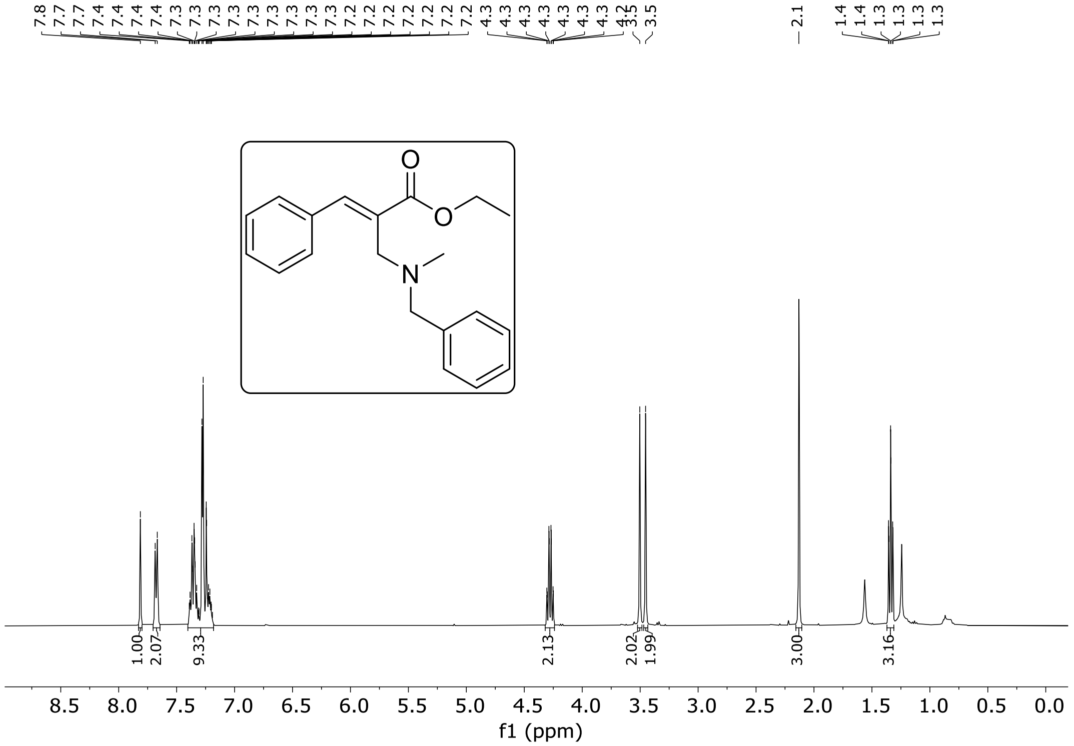

Figure S32. ^{13}C{^{1}H} NMR spectrum of Ethyl (*E*)-2-((benzyl(methyl)amino)methyl)-3-phenylacrylate (**23**). (100 MHz, chloroform-d) (>20:1 *dr*)

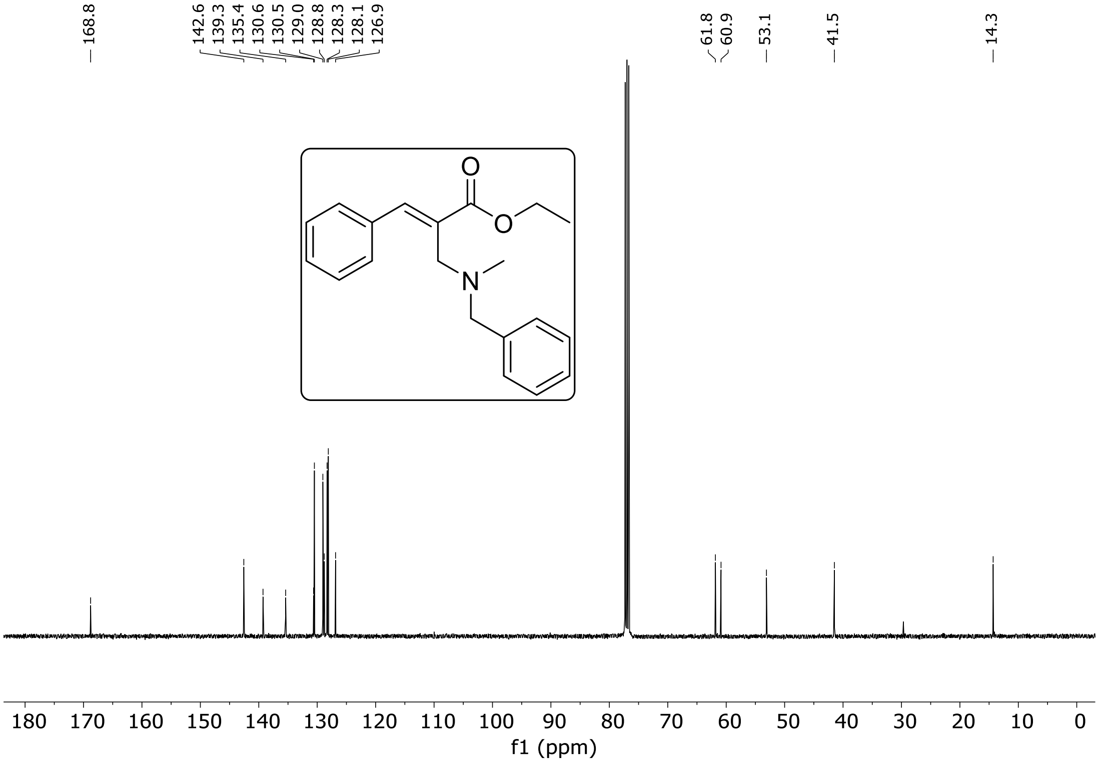

Figure S33. ^{1}H NMR spectrum of Ethyl (*E*)-2-(morpholinomethyl)-3-phenylacrylate (**24**). (400 MHz, chloroform-d) (>20:1 *dr*)

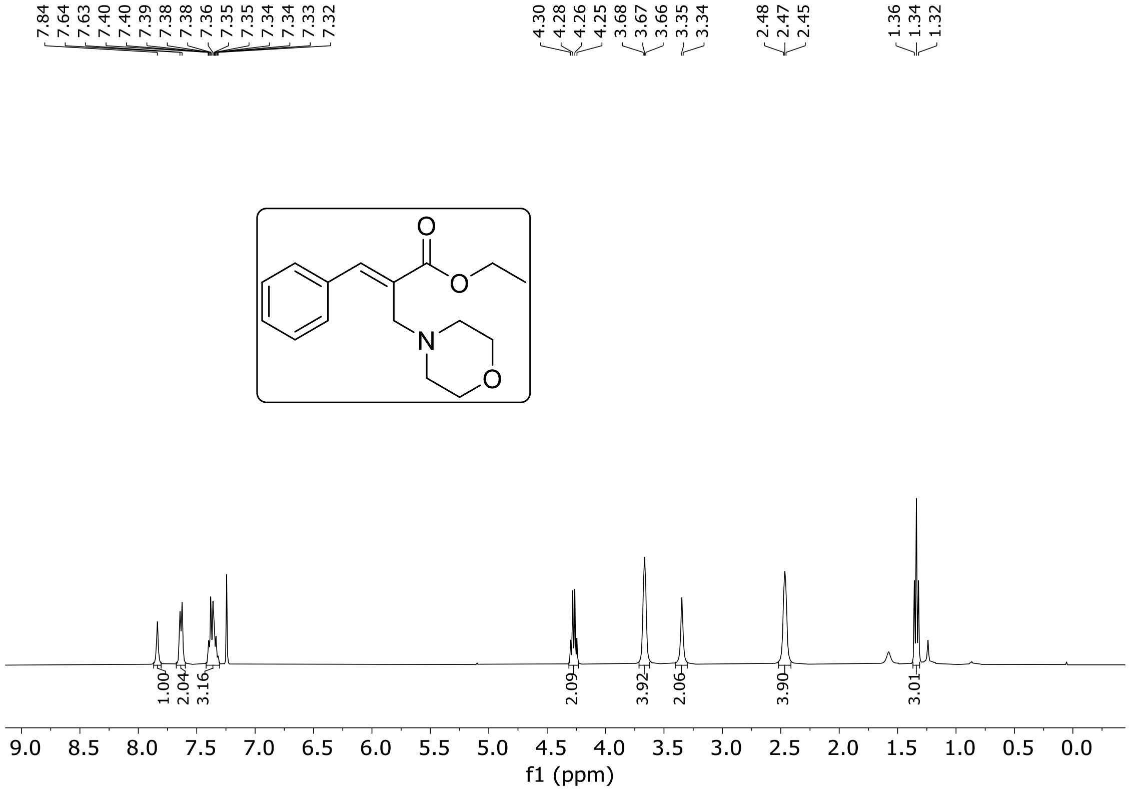

Figure S34. $^{13}C\{^1H\}$ NMR spectrum of Ethyl (*E*)-2-(morpholinomethyl)-3-phenylacrylate (**24**). (100 MHz, chloroform-d) (>20:1 *dr*)

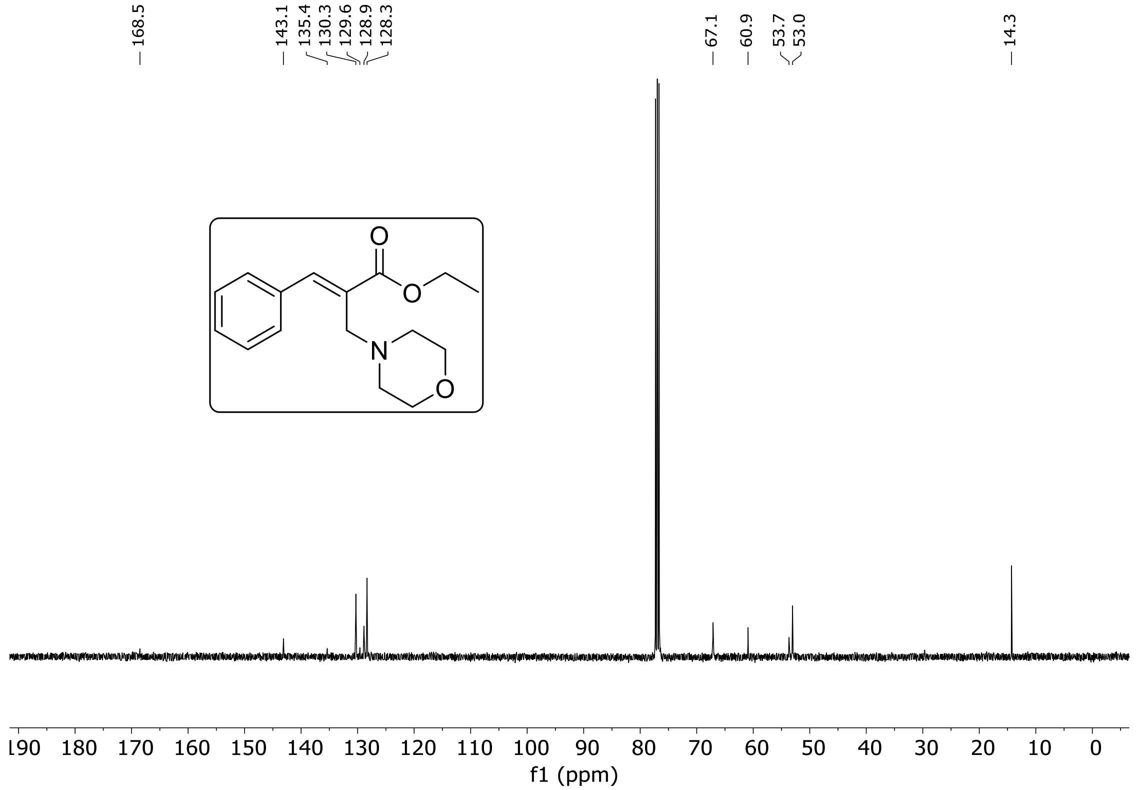

Figure S35. ^{1}H NMR spectrum of Ethyl (*E*)-2-((4,7-dihydrothieno[2,3-*c*]pyridine-6(*5H*)-yl)methyl)-3-phenylacrylate (**25**). (400 MHz, chloroform-d) (>20:1 *dr*)

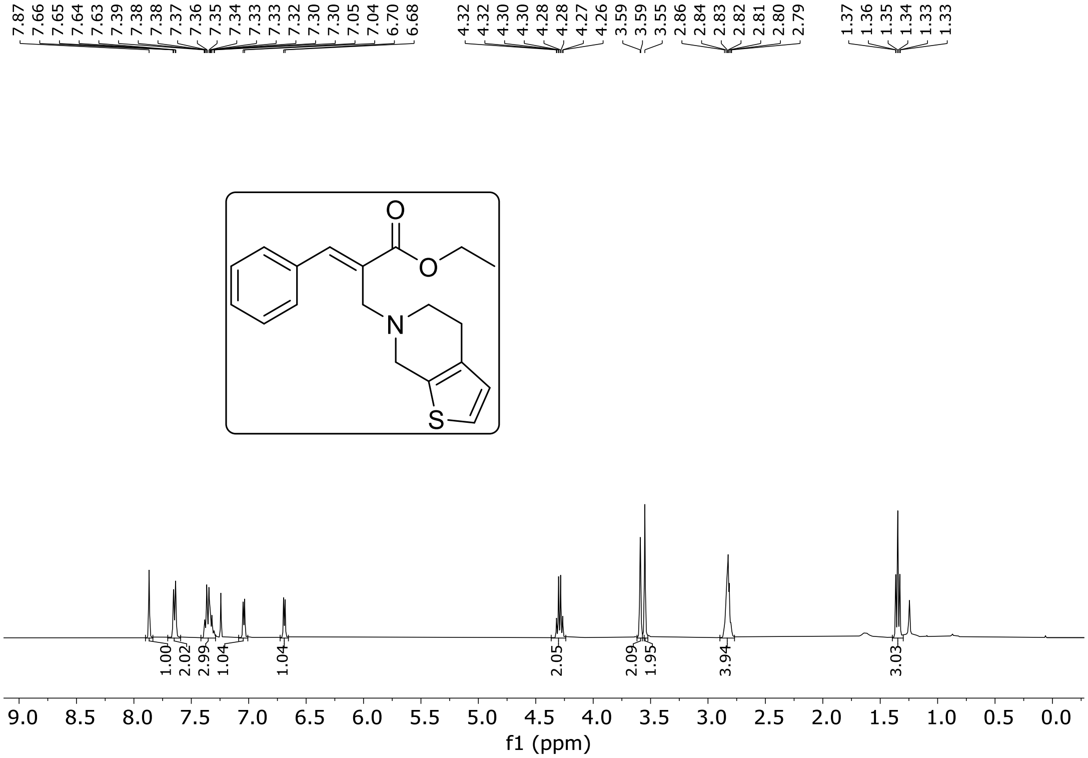

Figure S36. ^{13}C{^{1}H} NMR spectrum of Ethyl (*E*)-2-((4,7-dihydrothieno[2,3-*c*]pyridine-6(*5H*)-yl)methyl)-3-phenylacrylate (**25**). (100 MHz, chloroform-d) (>20:1 *dr*)

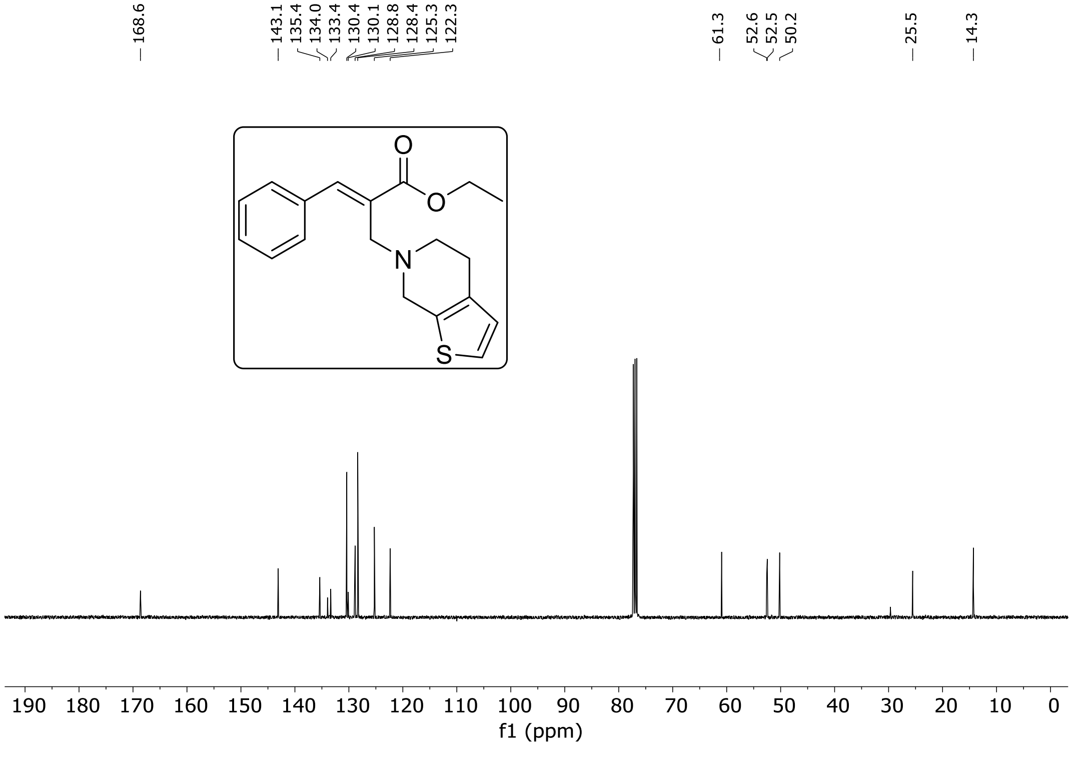

Figure S37. ^{1}H NMR spectrum of Ethyl (*Z*)-2-((phenethylthio)methyl)-3-phenylacrylate (**26**). (400 MHz, methanol-d$_4$) (10:1 *dr*)

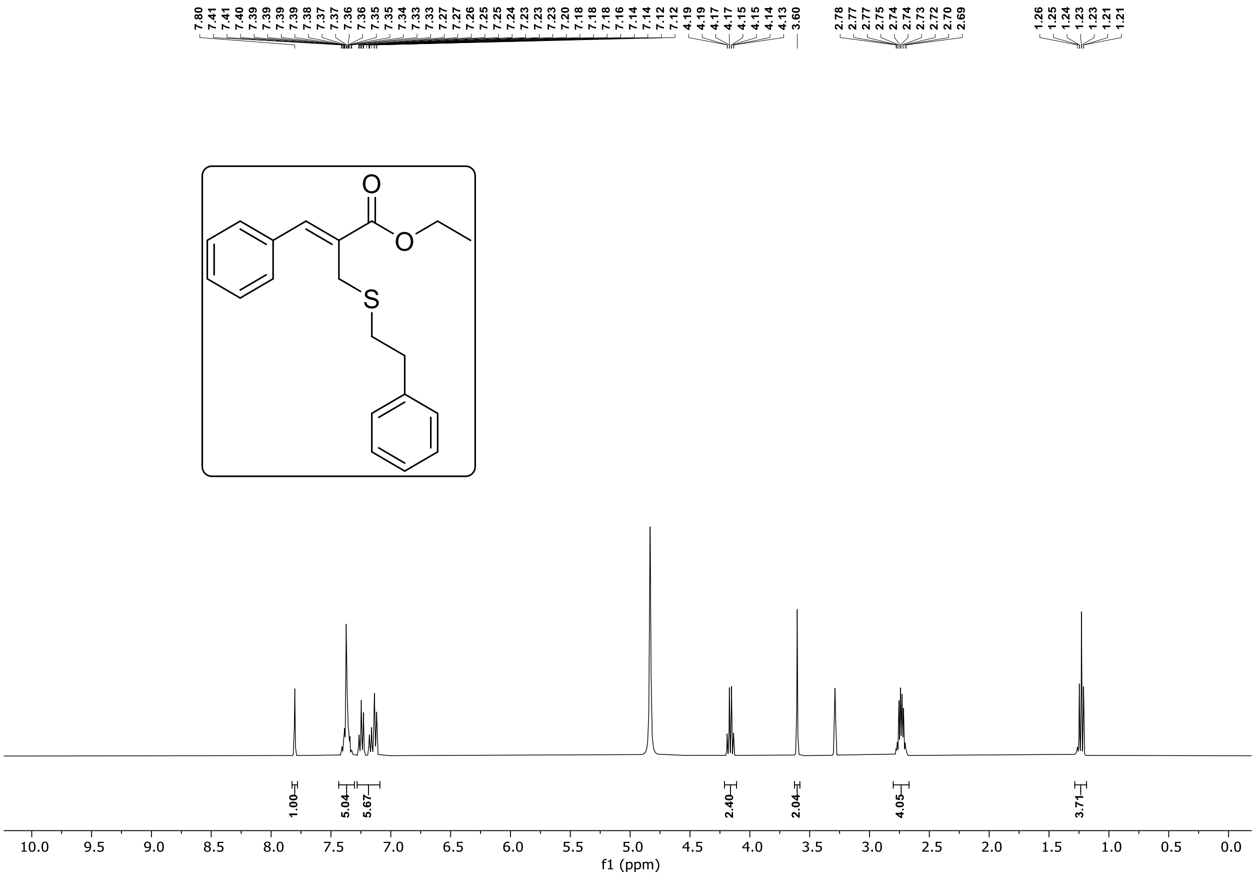

Figure S38. $^{13}C\{^{1}H\}$ NMR spectrum of Ethyl (*Z*)-2-((phenethylthio)methyl)-3-phenylacrylate (**26**). (100 MHz, methanol-d_4) (10:1 *dr*)

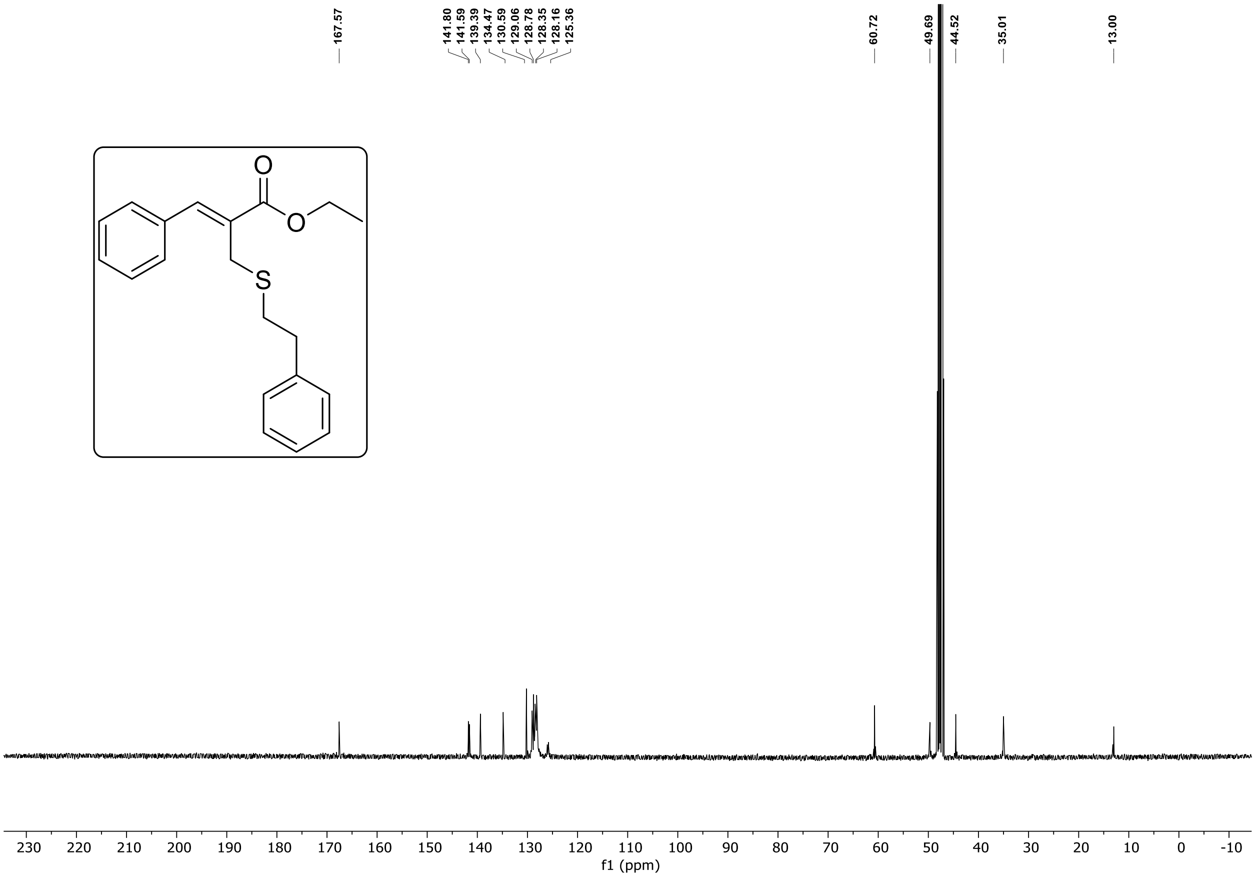

Figure S39. ^{1}H NMR spectrum of 3-Ethyl 1,1-dimethyl (*E*)-4-phenylbut-3-ene-1,1,3-tricarboxylate (**27**). (400 MHz, methanol-d$_4$) (4:1 *dr*)

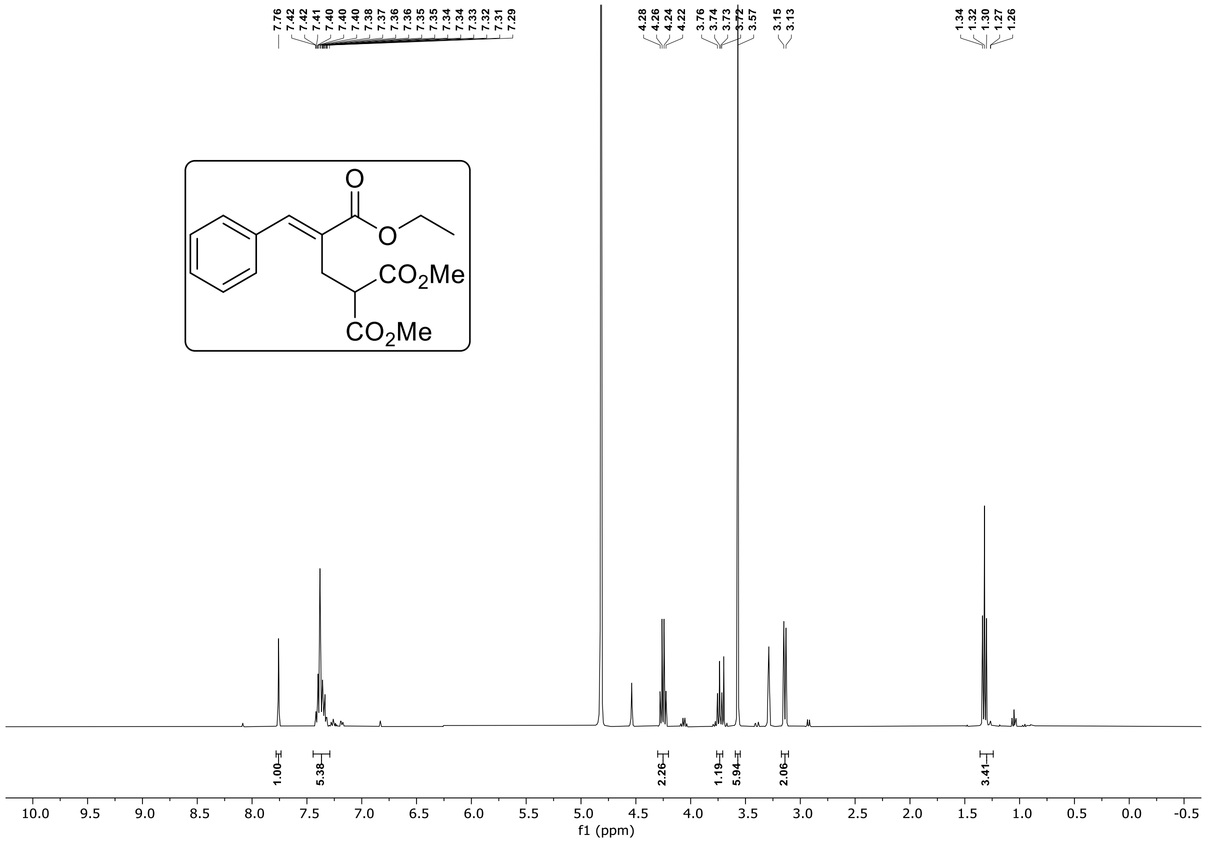

Figure S40. $^{13}C\{^{1}H\}$ NMR spectrum of 3-Ethyl 1,1-dimethyl (*E*)-4-phenylbut-3-ene-1,1,3-tricarboxylate (**27**). (100 MHz, methanol-d_4) (4:1 *dr*)

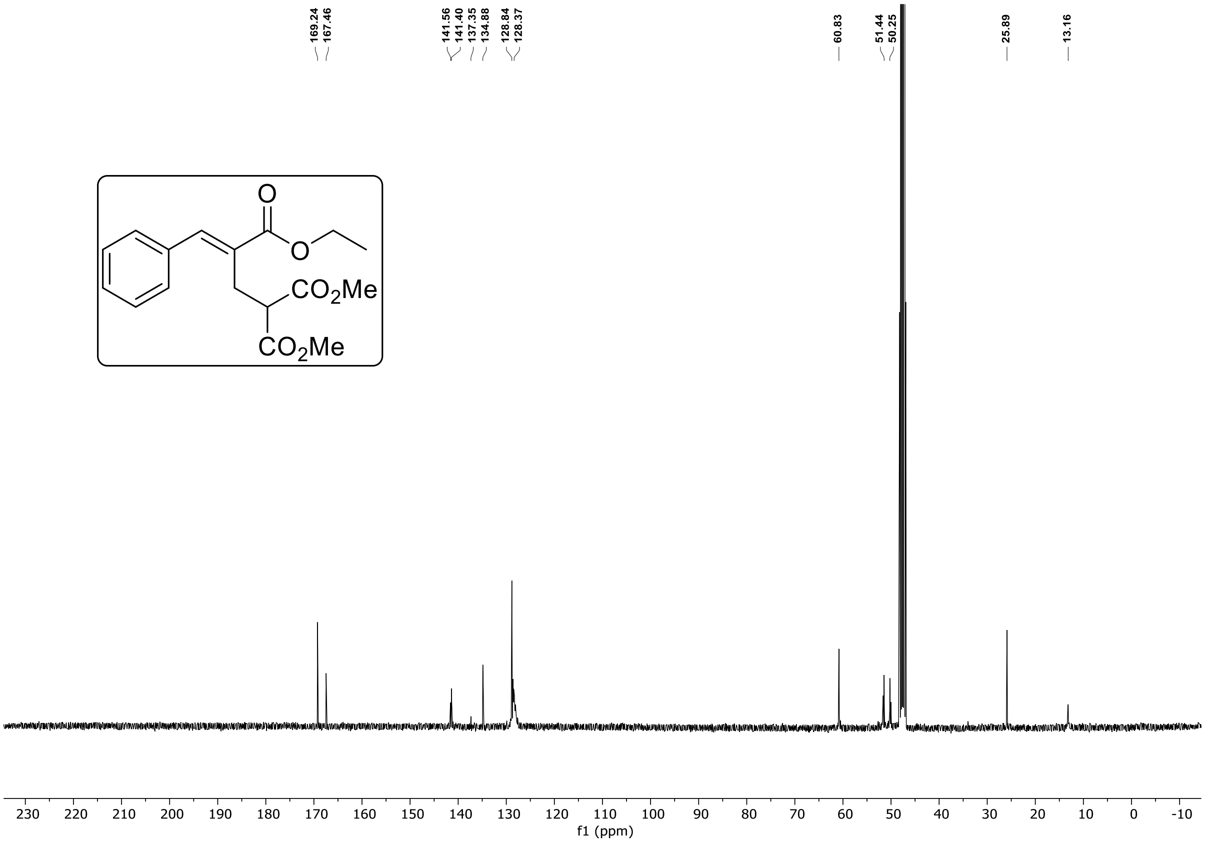

Figure S41. ^{1}H NMR spectrum of Ethyl (*E*)-2-((2-oxocyclohexyl)methyl)-3-phenylacrylate (**28**). (400 MHz, methanol-d$_4$) (10:1 *dr*)

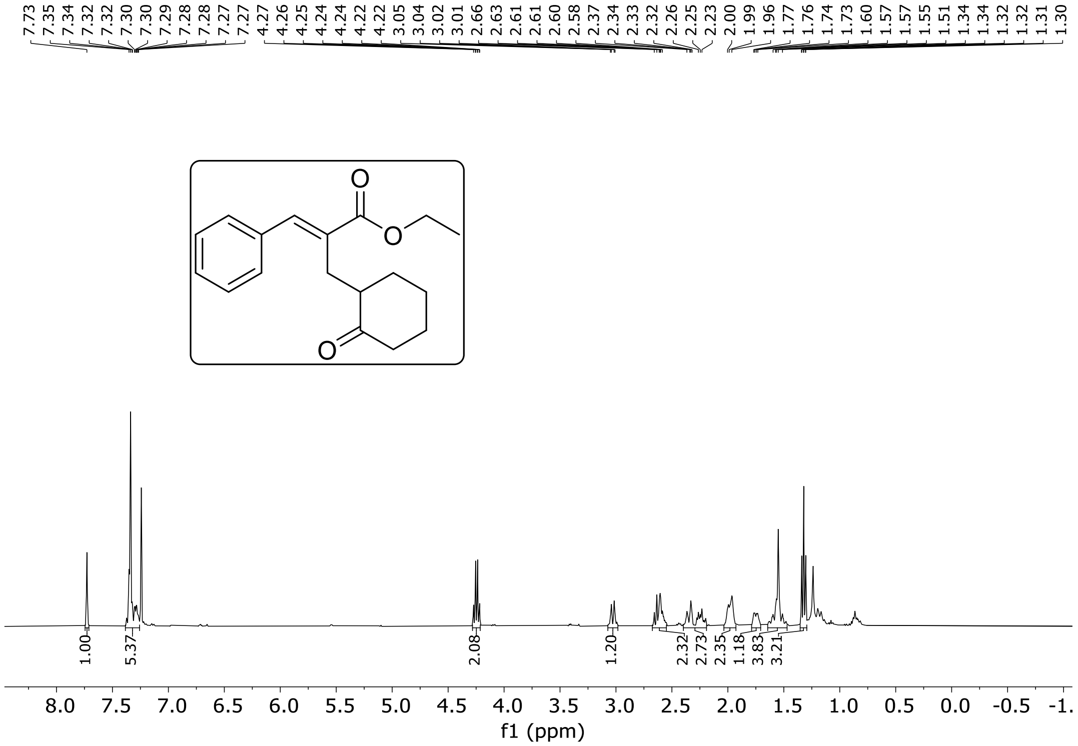

Figure S42. ^{13}C{^{1}H} NMR spectrum of Ethyl (*E*)-2-((2-oxocyclohexyl)methyl)-3-phenylacrylate (**28**). (100 MHz, chloroform-d) (10:1 *dr*)

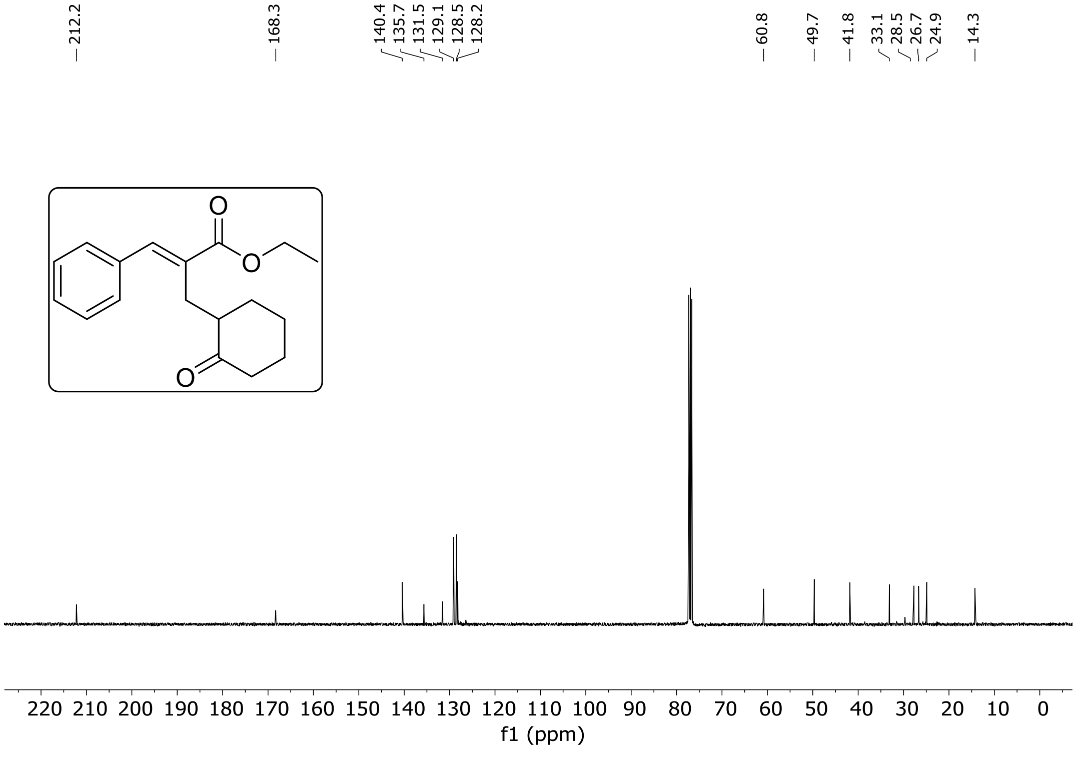

Figure S43. ^{1}H NMR spectrum of Ethyl (*E*)-1-(2-(ethoxycarbonyl)-3-phenylallyl)-2-oxocyclopentane-1-carboxylate ((*E*)-**29**). (400 MHz, methanol-d_4) (10:1 *dr*)

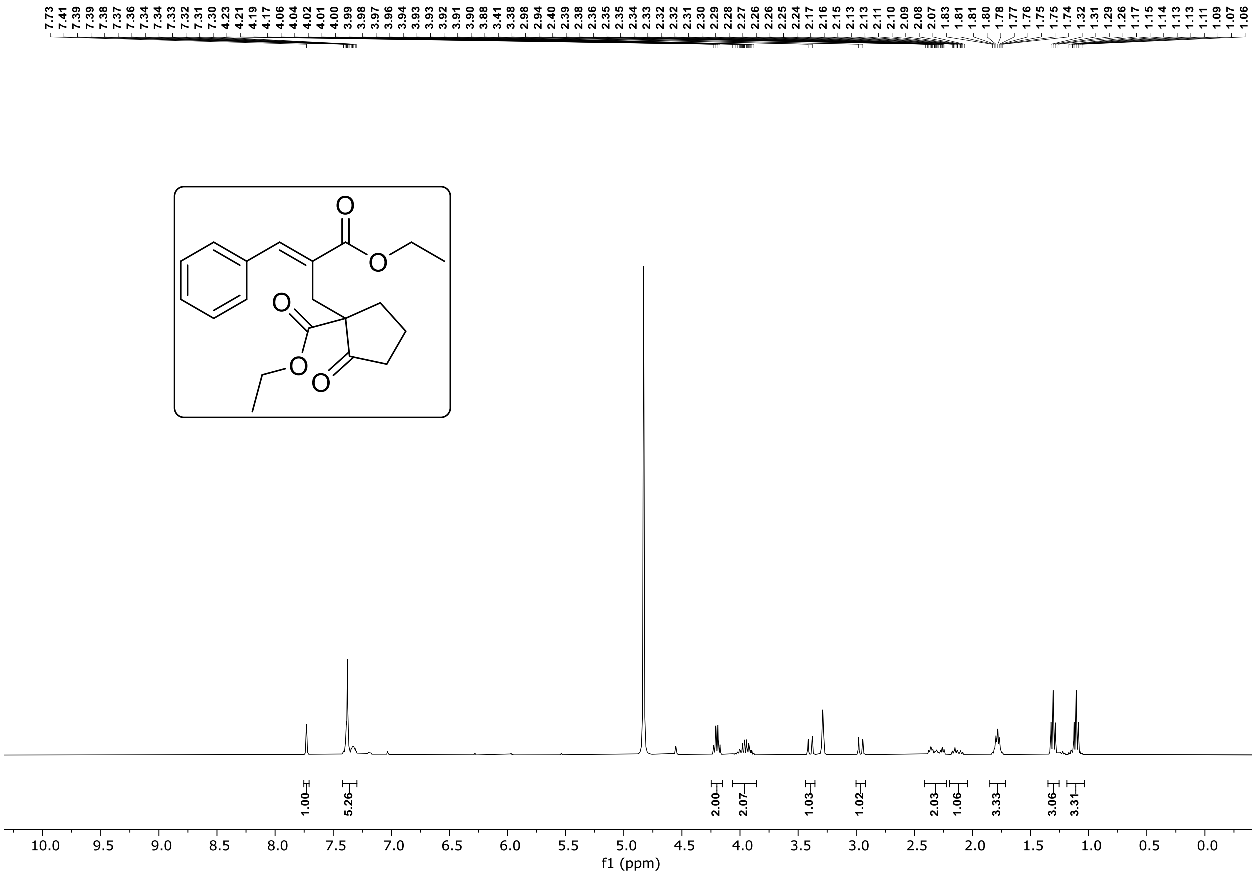

Figure S44. $^{13}C\{^{1}H\}$ NMR spectrum of Ethyl (*E*)-1-(2-(ethoxycarbonyl)-3-phenylallyl)-2-oxocyclopentane-1-carboxylate ((*E*)-**29**). (100 MHz, methanol-d$_4$) (10:1 *dr*)

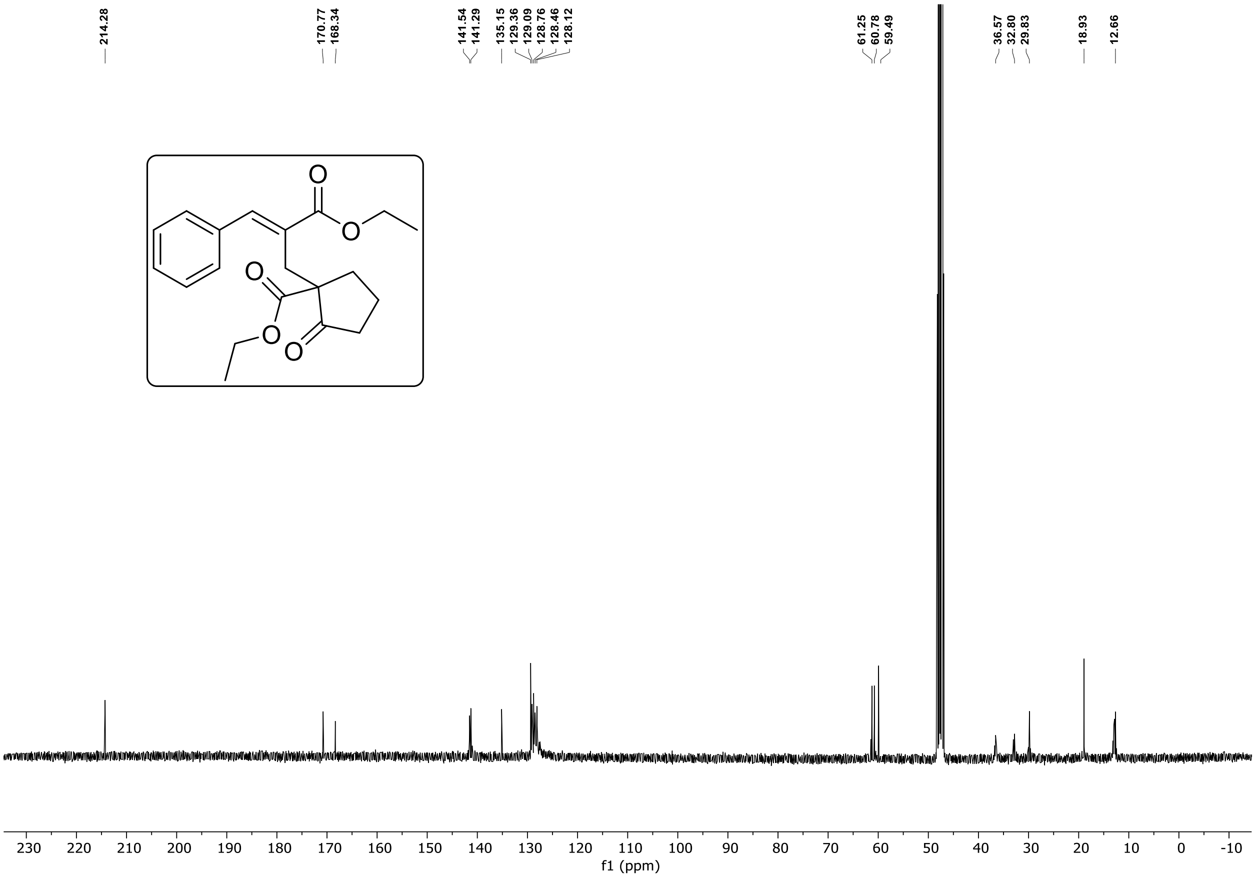

Figure S45. ^{1}H NMR spectrum of Ethyl 2-(fluoro(naphthalen-2-yl)methyl)acrylate (**1**). (400 MHz, chloroform-d)

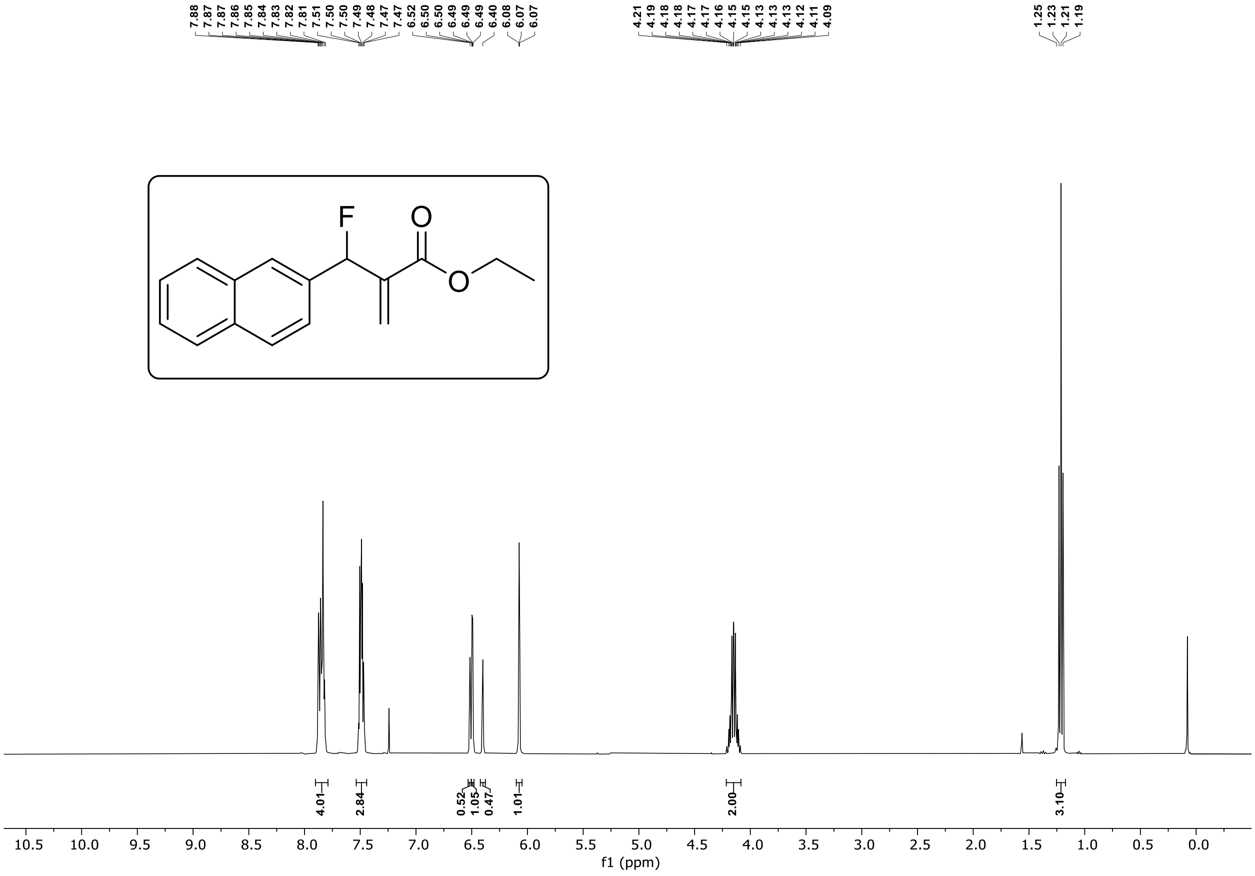

Figure S46. $^{13}C\{^{1}H\}$ NMR spectrum of Ethyl 2-(fluoro(naphthalen-2-yl)methyl)acrylate (**1**). (100 MHz, chloroform-d)

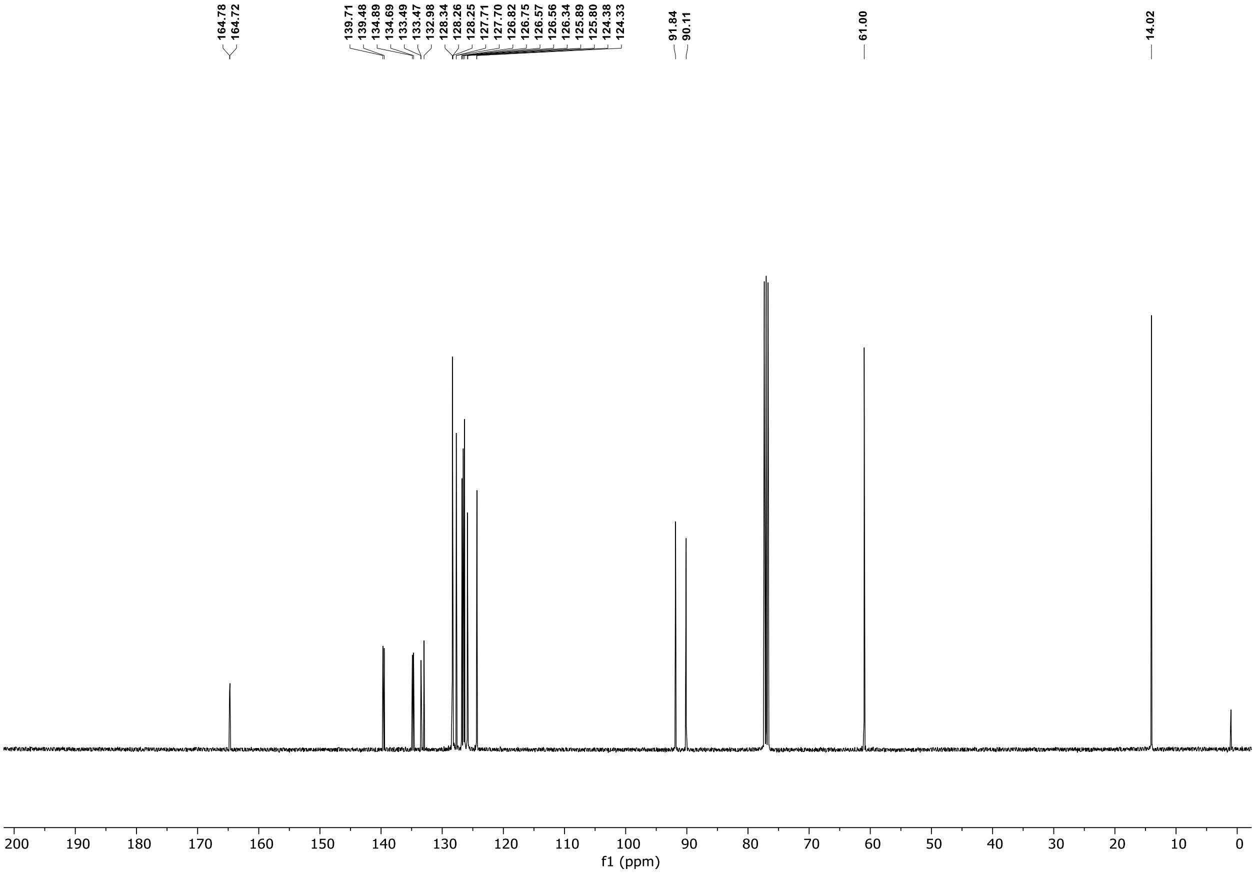

Figure S47. ^{19}F NMR spectrum of Ethyl 2-(fluoro(naphthalen-2-yl)methyl)acrylate (**1**). (376 MHz, chloroform-d)

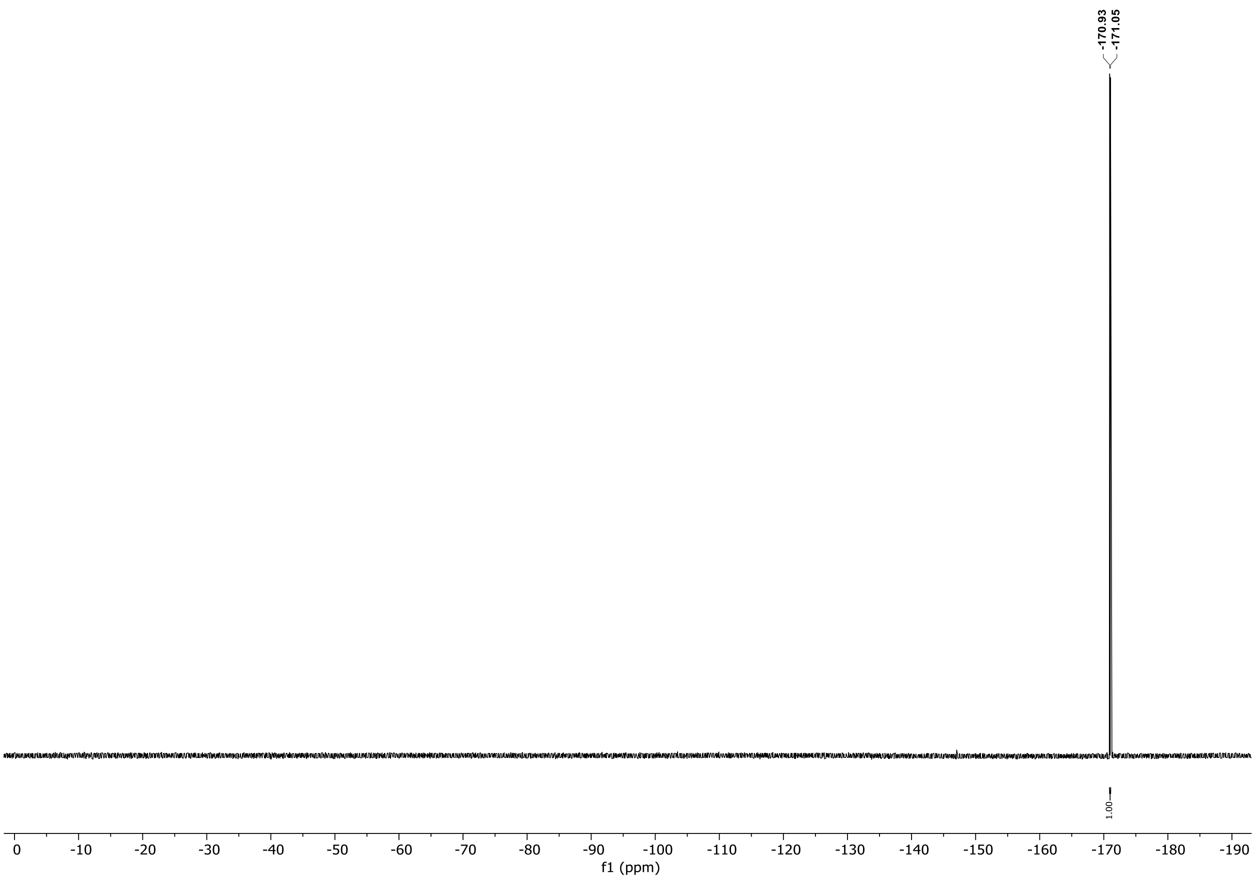

5. HPLC Chromatograms

Figure S48. HPLC Chromatogram of racemic ethyl (*E*)-1-(2-(ethoxycarbonyl)-3-phenylallyl)-2-oxocyclopentane-1-carboxylate ((*E*)-29). (Chiracel OJ-H, hexanes/*i*-PrOH 98:2, flow rate 1 mL/min, λ = 254 nm). The *dr* was determined as 7:1 by ^{1}H NMR analysis.

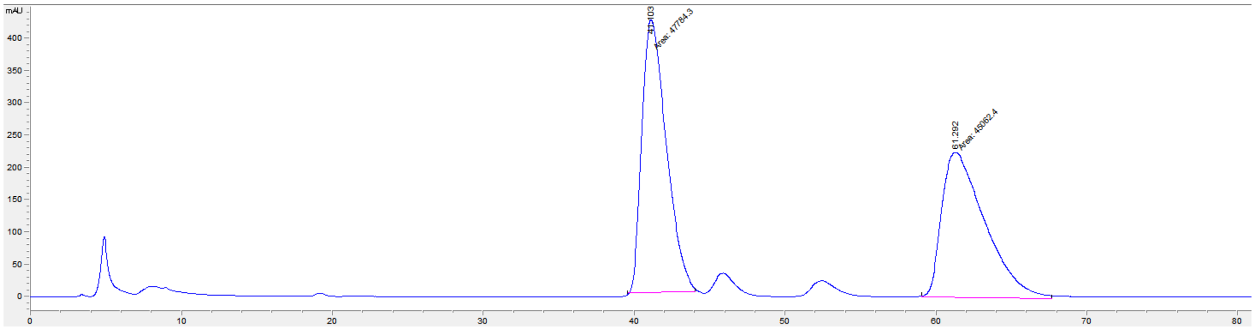

Figure S49. HPLC Chromatogram of non-racemic ethyl (*E*)-1-(2-(ethoxycarbonyl)-3-phenylallyl)-2-oxocyclopentane-1-carboxylate ((*E*)-29). (*ee* = 65%) (Chiracel OJ-H, hexanes/*i*-PrOH 98:2, flow rate 1 mL/min, λ = 254 nm). The *dr* was determined as 10:1 by ^{1}H NMR analysis.

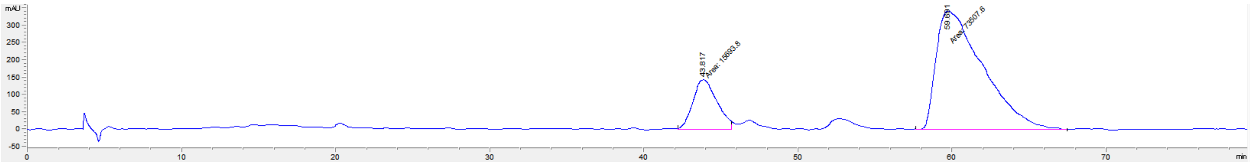

6. References

(1) a) Nishimine, T.; Fukushi, K.; Shibata, N.; Taira, H.; Tokunaga, E.; Yamano, A.; Shiro, M.; Shibata, N. Kinetic Resolution of Allyl Fluorides by Enantioselective Allylic Trifluoromethylation Based on Silicon-Assisted C-F Bond Cleavage. *Angew. Chem. Int. Ed.* **2014**, *53*, 517–520. b) Nishimine, T.; Taira, H.; Tokunaga, E.; Shiro, M.; Shibata, N. Enantioselective Trichloromethylation of MBH-Fluorides with Chloroform Based on Silicon-assisted C−F Activation and Carbanion Exchange Induced by a Ruppert–Prakash Reagent. *Angew. Chem. Int. Ed.* **2016**, *55*, 359–363. c) Okusu, S.; Okazaki, H.; Tokunaga, E.; Soloshonok, V. A.; Shibata, N. Organocatalytic Enantioselective Nucleophilic Alkynylation of Allyl Fluorides Affording Chiral Skipped Ene-ynes. *Angew. Chem. Int. Ed.* **2016**, *55,* 6744–6748.

(2) a) Zi, Y.; Lange, M.; Schultz, C.; Vilotijevic, I. Latent Nucleophiles in Lewis Base Catalyzed Enantioselective N-Allylations of N-Heterocycles. *Angew. Chem. Int. Ed*. **2019**, *58*, 10727–10731. b) Lange, M.; Zi, Y.; Vilotijevic, I. Enantioselective Synthesis of Pyrrolizin-1-ones via Lewis Base Catalyzed N-Allylation of N-Silyl Pyrrole Latent Nucleophiles. *J. Org. Chem.* **2020***, 85,* 1259–1269. c) Zi, Y.; Lange, M.; Vilotijevic, I. Enantioselective Lewis Base Catalyzed Phosphonyldifluoromethylation of Allylic Fluorides Using a C-silyl Latent Pronucleophile. *Chem. Commun.* **2020***, 56,* 5689–5692.

(3) L'Heureux, A.; Beaulieu, F.; Bennett, C.; Bill, D. R.; Clayton, S.; LaFlamme, F.; Mirmehrabi, M.; Tadayon, S.; Tovell, D.; Couturier, M. *J. Org. Chem.* **2010**, *75*, 3401–3411.

(4) Yang, W.; Chen, C.; Chan, K. S. Hydrodebromination of allylic and benzylic bromides with water catalyzed by a rhodium porphyrin complex. *Dalton Trans*. **2018**, *47*, 12879–12883.

(5) Balaraman, K.; Kyriazakos, S.; Palmer, R.; Thanzeel, F. Y.; Wolf, C. Selective Csp^3─F Bond Functionalization with Lithium Iodide. *Synthesis* **2022**, *54*, 4320-4328.

(6) Kumar, S.; Lange, M.; Zi, Y.; Görls, H.; Vilotijevic, I. Latent Pronucleophiles in Lewis Base Catalysis: Enantioselective Allylation of Silyl Enol Ethers with Allylic Fluorides. *Chem. Eur. J.* **2023**, *29*, e202300641.
